# Synthesis of some new distyrylbenzene derivatives using immobilized Pd on an NHC-functionalized MIL-101(Cr) catalyst: photophysical property evaluation, DFT and TD-DFT calculations†

**DOI:** 10.1039/d1ra00457c

**Published:** 2021-03-29

**Authors:** Esmaeil Niknam, Ali Mahmoodi, Farhad Panahi, Maryam Heydari Dokoohaki, Amin Reza Zolghadr, Ali Khalafi-Nezhad

**Affiliations:** Department of Chemistry, College of Sciences, Shiraz University Shiraz 71454 Iran panahi@shirazu.ac.ir panahichem@ymail.com; Department of Polymer Engineering and Color Technology, Amirkabir University of Technology Tehran Iran

## Abstract

In this study the catalytic application of a heterogeneous Pd-catalyst system based on metal organic framework [Pd–NHC–MIL-101(Cr)] was investigated in the synthesis of distyrylbenzene derivatives using the Heck reaction. The Pd–NHC–MIL-101(Cr) catalyst showed high efficiency in the synthesis of these π-conjugated materials and products were obtained in high yields with low Pd-contamination based on ICP analysis. The photophysical behaviors for some of the synthesized distyrylbenzene derivatives were evaluated. The DFT and TD-DFT methods were employed to determine the optimized molecular geometry, band gap energy, and the electronic absorption and emission wavelengths of the new synthesized donor–π–acceptor (D–π–A) molecules in the gas phase and in various solvents using the chemical model B3LYP/6-31+G(d,p) level of theory.

## Abstract 2

The Pd–NHC–MIL-101(Cr) catalyst was found to be an efficient heterogeneous catalyst in the synthesis of DSB and stilbene π-conjugated materials using Heck chemistry.

## Graphical abstract

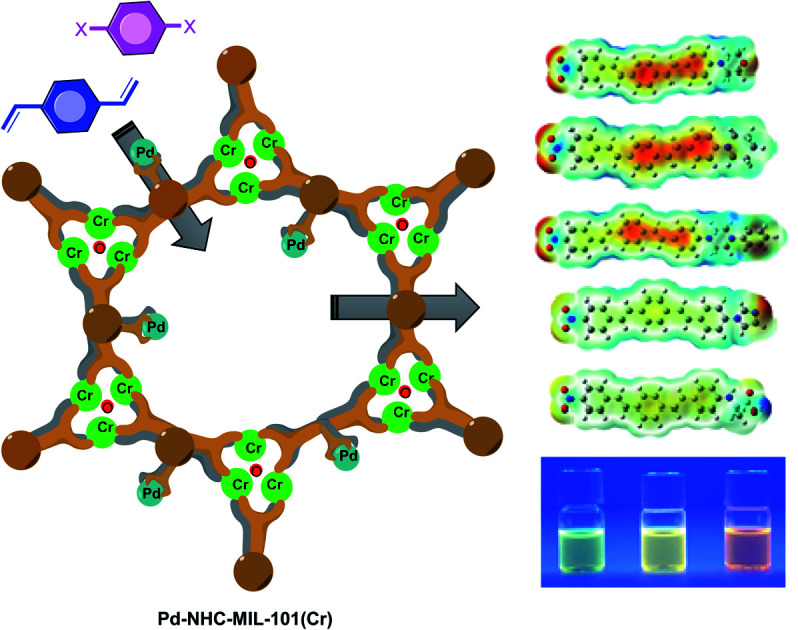

## Introduction

The synthesis of fluorescent compounds to be used in organic light emitting diodes (OLEDs),^1–5^ solar cells,^6,7^ organic field effect transistors (OFETs),^8,9^ sensing,^10–12^ and fluorescent probes^13–16^ is highly considered. Stilbene compounds are a significant class of fluorescent organic π-conjugated compounds, which are widely used in the above mentioned applications.^17–26^ Due to the systematic relationship between the fluorescence properties of the fluorescent materials and their chemical structures, stilbenes are an interesting class of compounds which permit us to simply fine tune the photophysical properties *via* available chemical modifications.^27–31^ To synthesize stilbenes, different organic methodologies such as Wittig reaction,^32–34^ Horner–Wadsworth–Emmons reaction,^35^ catalytic aldehyde olefinations,^36^ and Mizoroki–Heck reaction^37,38^ have been developed. Palladium-catalyzed coupling reactions are key tools in stilbene synthesis because they consist of a family of cross coupling reactions, allowing diversity oriented synthesize of stilbenes.^39–42^ Thus, Mizoroki–Heck reaction has been extensively used in the synthesis of stilbene compounds.^43,44^

**Scheme 1:**
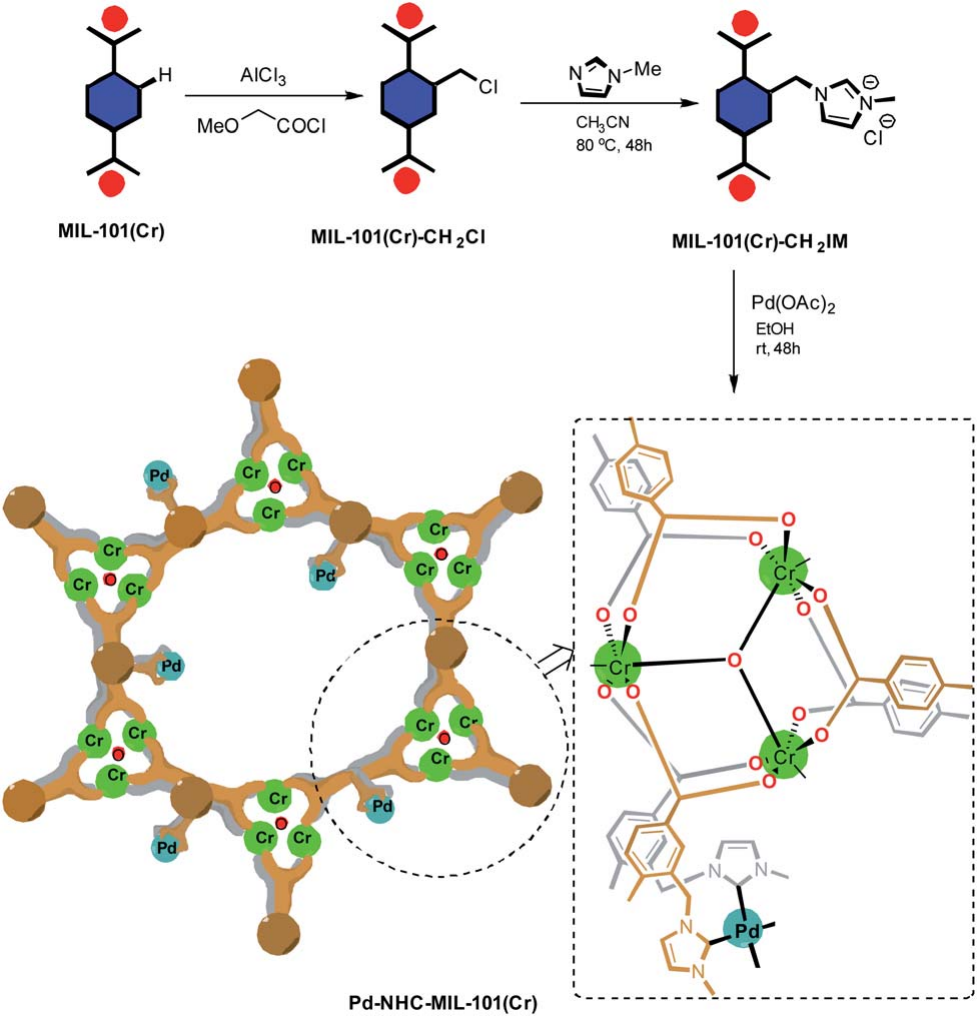
Synthetic rote to synthesize [Pd–NHC–MIL-101(Cr)] catalyst.

In this work, in continuation of our program on the synthesis of stilbene derivatives,^45–50^ a highly efficient heterogeneous catalyst system [Pd–NHC–MIL-101(Cr)]^51^ was introduced to be applied in the synthesis of stilbene derivatives using Mizoroki–Heck coupling reaction.^52–57^ The Pd–NHC–MIL-101(Cr) catalyst system showed remarkable catalytic activity in the Heck reaction^51^ and in order to further show its utility in organic synthesis we investigate its applicability in the synthesis of distyrylbenzenes (DSBs). The synthetic pathway toward synthesis of [Pd–NHC–MIL-101(Cr)] catalyst system is shown in Scheme 1.

## Results and discussion

### Catalytic activity evaluation of Pd–NHC–MIL-101(Cr) catalyst in the synthesis of distyrylbenzenes (DSBs)

In order to show the catalytic applicability of [Pd–NHC–MIL-101(Cr)] catalyst in the synthesis of DSBs and stilbenes, a model reaction was selected and different conditions were checked to obtain high yields of desired products (Table 1).

**Table 1:** Optimization of the Pd–NHC–MIL-101(Cr)-catalyzed Heck reaction between aryl halides and 1,4-distylbenzene^a^

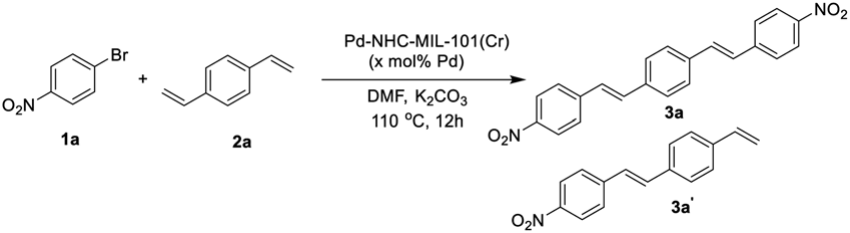				
Entry	*x*	1a : 2a ratio	Yield 3a^b^	Yield 3a′^b^
1	1.5	1 : 2	6	85 (79)
2	1.5	2 : 1	70	20
3	1.5	1 : 2.2	5	88 (81)
4	1.5	2.2 : 1	83 (77)	2
5	1.25	1 : 2.2	6	78 (71)
6	1.25	2.2 : 1	70	8
7	2.0	1 : 2.2	4	87 (78)
8	2.0	2.2 : 1	84	20
9	1.5	1 : 2.2	20	58^c^
10	1.5	2.2 : 1	71	18^c^

a Reaction conditions: 1a (1.0 mmol), 2a (based on the ratio), Pd–NHC–MIL-101(Cr) (*x* mol%), DMF (5 mL), K_2_CO_3_ (2.5 mmol), 110 °C, 12 h.

b NMR yield.

c The Pd/C was used as catalyst. The yields in parentheses related to isolated yields.

As shown in Table 1, using different ratios of starting materials in the presence of Pd–NHC–MIL-101(Cr) catalyst, it is possible to obtain both compounds 3a and 3a′ in high yields. In order to synthesize DSBs in high yield, the ratio of aryl halide to 1,4-distylbenzene was selected 2.2 to 1 (Table 1, entry 4). Also, the best yield for mono-substituted product was achieved using 1 : 2.2 ratios for 1a : 2a (Table 1, entry 3). No improvement in the reaction yield was observed by increasing the catalyst loading more than 1.5 mol% (Table 1, entries 5–8).^58^ Using Pd/C as a traditional catalyst,^59–62^3a′ was obtained in lower yield of 58% (same conditions and stoichiometry), demonstrating important role of MOF structure in homoselectivity^63^ to obtain 3a′ in high yield (Table 1, entries 9 & 10). Also, the ICP analysis of the product using Pd–NHC–MIL-101(Cr) catalyst showed less than 2 ppm of Pd while the amount of Pd-content for the product obtained using Pd/C catalyst was around 16 ppm. This experiment showed that the efficacy of this Pd MOF-based catalyst in the synthesis of this class of π-conjugated materials with low Pd-contamination which is very important in their applications.

Next we checked the synthesis of DSB derivatives using the reaction of 1,4-dibromobenzene and styrene (Table 2). The Pd–NHC–MIL-101(Cr) catalyst can effectively catalyze this coupling reaction and it is possible to control the reaction to obtain both 3a and 3a′′ in high yields. The synthesis of 3a′′ is important because it can be used for the synthesis of unsymmetrical DSB incorporating two different functional groups in the ends of pi-conjugated system.^50^ Using 1.5 mol% of Pd–NHC–MIL-101(Cr) catalyst and ratio of 1 : 2.2 for 4a : 5a, DSB 3a was obtained in 84% isolated yield (Table 2, entry 3). Employing the same catalyst loading and reveres ratio of 4a : 5a (2.2 : 1), compound 3a′′ was obtained in 86% (Table 2, entry 4). Again, in order to check the homoselectivity of the Pd–NHC–MIL-101(Cr) catalyst in mono-functionalization using Heck chemistry the reaction was checked using a Pd/C catalyst. Using this catalyst system compound 3a′′ was obtained in lower yield of 63% (same conditions and stoichiometry). This experiment also represents the key role of MOF structure in homoselectivity (Table 2, entries 5 & 6). The Pd content of the products in this reaction was also evaluated using ICP analysis and it was observed that the obtained product using Pd–NHC–MIL-101(Cr) catalyst has only 3.1 ppm of Pd, while for the product obtained in the presence of homogeneous is around 22 ppm. Accordingly, this heterogeneous Pd catalyst system based on MOF is efficient in the synthesis of DSBs with low Pd-contamination.

**Table 2:** Optimization of the Pd–NHC–MIL-101(Cr)-catalyzed Heck reaction between 1,4-dibromobenzene and stylbenzene^a^

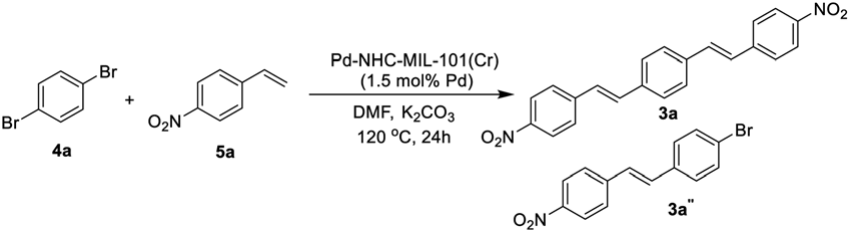			
Entry	4a : 5a ratio	Yield 3a^b^	Yield 3a′′^b^
1	1 : 2	82 (76)	12
2	2 : 1	4	80 (73)
3	1 : 2.2	84 (77)	8
4	2.2 : 1	2	86 (79)
5	1 : 2.2	69	19^c^
6	2.2 : 1	21	63^c^

a Reaction conditions: 1a (1.0 mmol), 2a (based on the ratio), Pd–NHC–MIL-101(Cr) (1.5 mol%), DMF (5 mL), K_2_CO_3_ (2.5 mmol), 110 °C, 12 h.

b NMR yield.

c The Pd/C was used as catalyst. The yields in parentheses related to isolated yields.

**Fig. 1:**
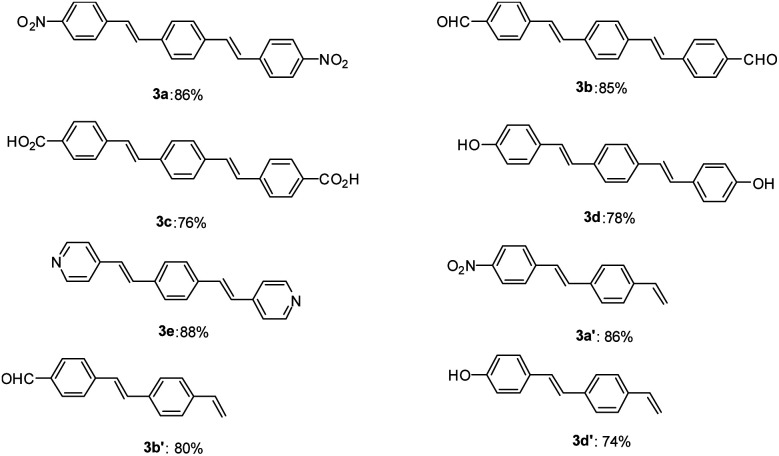
Synthesis of diverse symmetrical DSBs and vinyl-functionalized stilbenes using Pd–NHC–MIL-101(Cr) catalyst. Reaction conditions for compound (3a–e): 1,4-divinylbenzene (0.55 mmol), aryl halide (1.0 mmol), base (2.0 mmol), amount of catalyst 12.0 mg (1.5 mol%), solvent (5 mL), 12 h at 110 °C. Reaction conditions for compound (3a′, 3b′ and 3d′): 1,4-divinylbenzene (1.1 mmol), aryl halide (1.0 mmol), base (2.0 mmol), amount of catalyst 12.0 mg (1.5 mol%), solvent (5 mL), 12 h at 110 °C. All yields correspond to the isolated product.

After optimization of the reaction conditions, in order to show the applicability of this catalyst system in synthesis of stilbene and DSBs, some different derivatives were synthesized and results are depicted in Fig. 1.

As shown in Fig. 1, both electron-withdrawing and electron-donating groups on aryl rings worked well with this methodology. The synthesis of these DSBs is important. For example, compound 3b derivatives were used as an amine-sensitive dye for detection of proteins.^64^ These stilbene derivatives were also used for the preparation of polycyclic aromatic hydrocarbons (PAHs) and nanographene.^65^ Synthesis of hydroxylated stilbenes is important in biological application point of view and using this catalyst system, compounds 3d and 3d′ was successfully synthesized in high yields.^66^ Pyridine-based stilbenes are important in the preparation of porous coordination polymers.^67^

**Scheme 2:**
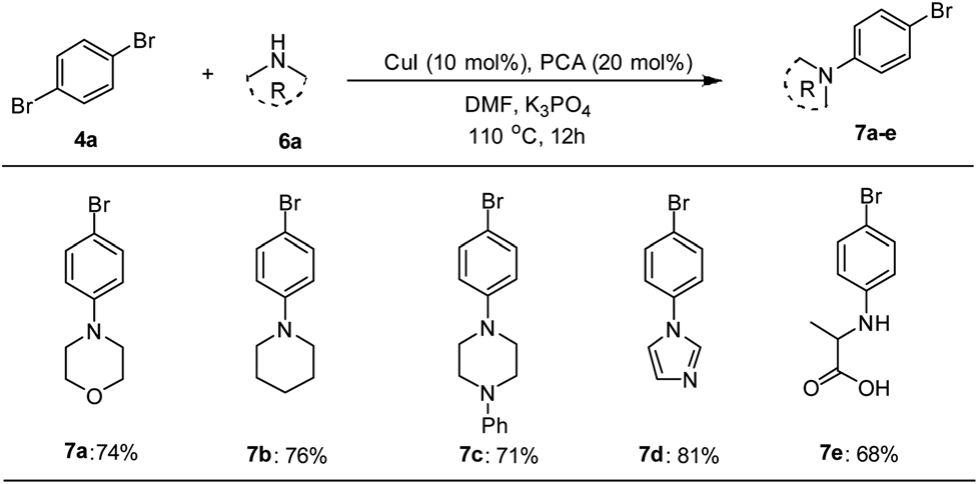
Synthesis of amine-functionalized aryl bromides using Cu-catalyzed *N*-arylation reaction.^*a*,*b a*^Reaction conditions: 1,4-dibromobenzene (1.0 mmol), amine (1.0 mmol), base (2.0 mmol), CuI catalyst (10.0 mol%), picolinic acid (PCA, 20 mol%), DMF (5 mL), 12 h at 110 °C. ^*b*^Isolated yield.

The catalytic applicability of Pd–NHC–MIL-101(Cr) catalyst system was also investigated in the synthesis of unsymmetrical DSBs under optimized conditions. First, some amine-functionalized aryl bromides were synthesized using a Cu-catalyzed *N*-arylation reaction based on a known procedure in the literature (Scheme 2).^68^

**Fig. 2:**
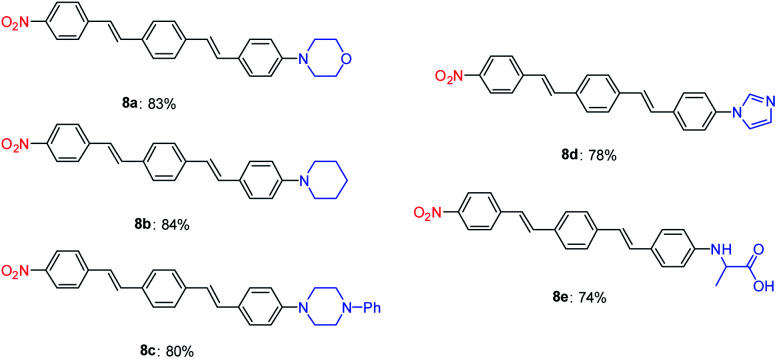
Synthesis of diverse D–π–A DSBs using Pd–NHC–MIL-101(Cr) catalyst.^*a*,*b a*^Reaction conditions: 3a′ (1.0 mmol), aryl halide 7a–e (1.0 mmol), base (2.0 mmol), Pd–NHC–MIL-101(Cr) catalyst (12.0 mg, 1.5 mol%), DMF (5 mL), 12 h at 110 °C. ^*b*^Isolated yield.

The Mizoroki–Heck coupling reaction between synthetic amine-functionalized aryl halides (7a–e) and compound 3b′ in the presence of Pd–NHC–MIL-101(Cr) catalyst afforded D–π–A systems in high isolated yields (Fig. 2).

### Photophysical properties investigation of compounds 8a–e

**Fig. 3:**
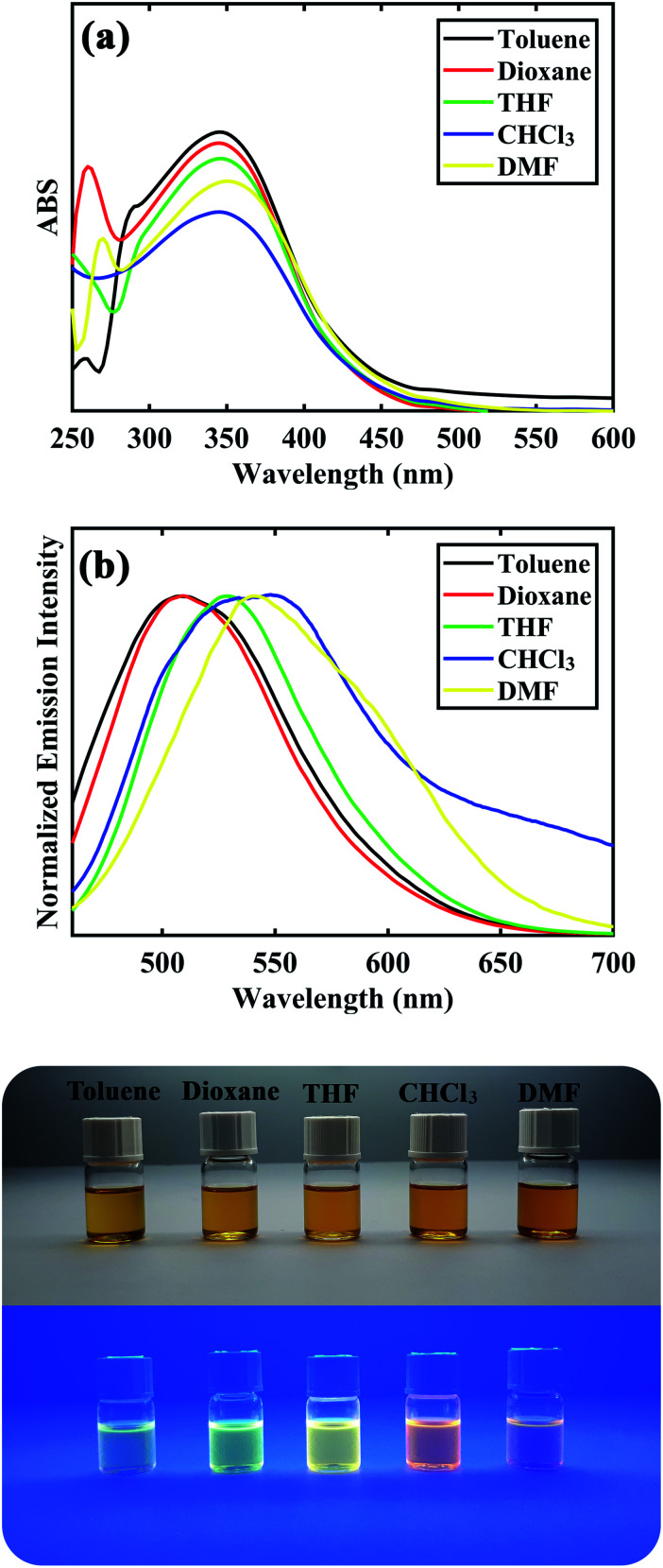
UV-Vis spectra (a) and emission spectra (b) of 8c at concentration of 10^−5^ M in different solvents. The photographs of the compound in different solutions [from left to right, toluene, dioxane, tetrahydrofuran (THF), chloroform (CHCl_3_), and dimethylformamide (DMF)] were taken under natural daylight simulator (D65) lamps (top image), and irradiation of A-Class UV lamps (bottom image).

After synthesizing and characterization of D–π–A DSBs, their photophysical properties were investigated and results are depicted in Table 3 and Fig. 3. All of the distyrylbenzene derivatives showed a broad absorption band between 328–355 nm corresponding to the intramolecular charge transfer (ICT) transfer between donor and acceptor moieties in the molecules. Solvent polarity had minimal effect on the absorption band of all compounds showing their low dipole moment at the ground state. The compounds were found to be fluorescence in all solvents with an emission maximum between 496–550 nm. The large stokes shifts with values between 8445–12 349 cm^−1^ for the samples suggest that the fluorescence could be due to intramolecular charge transfer (ICT). The emission spectra experienced a red shift from 507, 501, 508, 496, and 507 in toluene (least polarity) to 540, 532, 532, 533, and 541 in DMF (most polarity) for compounds 8a to 8e, respectively. As shown in Fig. 3, compound 8c showed a blue-green fluorescence under UV lamp in toluene and its fluorescence changed to green, yellow, and orange hue upon increasing solvent polarity. This trend was also observed for other distyrylbenzene derivatives suggesting a strong positive solvatochromic effect for the compounds (see ESI†).

**Table 3:** Photophysical data for DSB derivatives in different solvents

Solvent	*λ*_ab_ (nm)	*λ*_em_ (nm)	Stock shifts (cm^−1^)	*ε* (L mol^−1^ cm^−1^)	*E* (eV)
8a=M					
DMF	355	540	9650	96 836	3.49
CHCl_3_	355	552	10 053	73 757	3.49
THF	355	529	9265	94 671	3.49
Dioxane	355	509	8522	122904	3.49
Toluene	355	507	8445	88 301	3.49

8bP					
DMF	355	532	9372	60 756	3.49
CHCl_3_	345	535	10 293	53 302	3.59
THF	352	524	9325	67 162	3.52
Dioxane	352	506	8646	62 334	3.52
Toluene	350	501	8611	64 526	3.54

8cZ					
DMF	342	532	10 442	52 370	3.62
CHCl_3_	340	545	11 063	65 654	3.65
THF	340	527	10 436	69 341	3.65
Dioxane	340	511	9842	56 285	3.65
Toluene	340	508	9726	65 293	3.65

8dI					
DMF	330	533	11 541	97 190	3.75
CHCl_3_	330	557	12 349	80 833	3.75
THF	330	530	11 435	87 939	3.75
Dioxane	328	504	10 646	98 910	3.78
Toluene	330	496	10 141	85 460	3.75

8eA					
DMF	350	541	10 087	45 461	3.54
CHCl_3_	345	550	10 803	39 392	3.59
THF	345	529	10 081	49 873	3.59
Dioxane	345	509	9339	53 013	3.59
Toluene	345	507	9261	55 189	3.59

The stabilization of the excited state by more polar solvents was the reason for the observed solvatochromism. It should be noted that the more solvent dependency of emission spectra compared to that of absorption spectra for all compounds could be attributed to more ICT characteristic of the samples in their excited state than that of their ground states.^69–71^

The pH sensitivity of D–π–A DSBs were also evaluated and results are summarized in Table 4. As detailed in this table, upon decreasing the pH from 7 to 3, no meaningful change was observed in emission band of the samples. With further decreasing of the pH from 3 to 1, a weak blue shift with values between 15 to 25 nm was observed for the fluorescent compounds. The observed blue shift could be assigned to diminishing of intramolecular charge transfer (ICT) when the chromophores were protonated by TFA. Surprisingly, a strong red shift with values between 55–83 nm was detected for the compound 8c in strong acidic condition.

**Table 4:** The pH-sensitivity behavior of synthetic D–π–A DSBs 8a–e

Comp.	*λ*_em_ (nm) pH = 7	*λ*_em_ (nm) pH = 6	*λ*_em_ (nm) pH = 5	*λ*_em_ (nm) pH = 4	*λ*_em_ (nm) pH = 3	*λ*_em_ (nm) pH = 2	*λ*_em_ (nm) pH = 1
8a	552	552	552	552	552	552	552
					534	533	530
8b	535	535	535	535	535	535	535
					519	518	514
8c	545	545	545	545	545	545	545
					530	529	524
8d	557	557	557	557	557	557	557
					537	537	533
8e	550	550	550	550	550	528	526
					531	605	633

### DFT and TD-DFT calculations

**Fig. 4:**
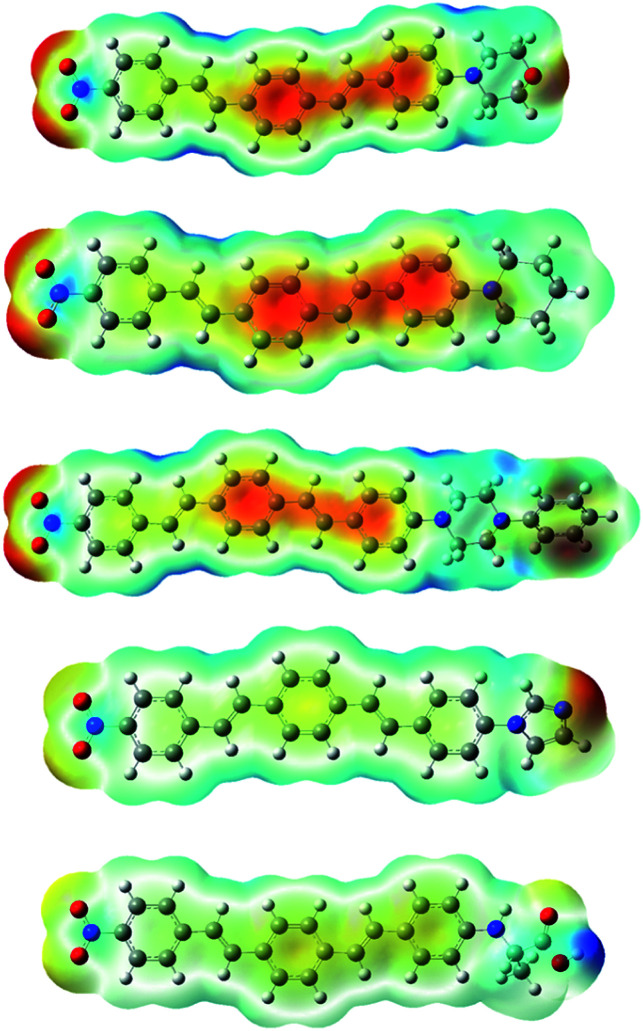
Optimized structures of 8a–e molecules at the B3LYP/6-31+G(d,p) level in the gas phase and electrostatic potential maps. ESP contours are color-coded from red (negative) to blue (positive).

In order to further clarify the experimental results, the optimized molecular structures of compounds 8a–e DSBs are illustrated in Fig. 4 using density functional theory (DFT) at the B3LYP level. In this work, the B3LYP-D3 and ωB97XD functional methods which include empirical dispersions were also employed in calculations and their computed maximum absorption wavelengths were found to be more deviated from experimental results.

These DSBs are D–π–A molecules consisting of the same electron withdrawing nitrobenzene moiety as well as different electron donating centers (morpholine, piperidine, piperazine, imidazole, and alanine), which are connected by π-conjugation in the middle. To illustrate the electronic distribution around molecular surface and also to probe the sites of electrophilic attack (negative potential) and nucleophilic reaction (positive potential) for investigated molecular systems, molecular electrostatic potential (MEP) surfaces were obtained. It is clearly seen in Fig. 4, in the MEP surface for the 8a–e derivatives, oxygen atoms of nitro groups and the center conjugated moieties through the π-bridge illustrate regions of negative electrostatic potential (electron-rich) while the hydrogen atoms carry the most positive potentials.

**Fig. 5:**
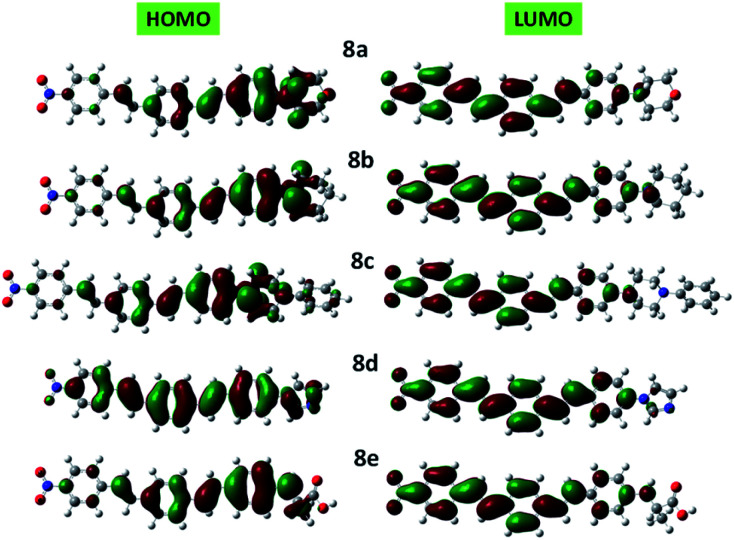
DFT computed HOMO and LUMO diagrams of 8a–e compounds at the B3LYP/6-31+G(d,p) level.

Clear elucidation of electron density distribution on the highest occupied molecular orbital (HOMO) and lowest unoccupied molecular orbital (LUMO) of the compounds 8a–e configurations were plotted in Fig. 5. The HOMO of the compounds 8a–e is mainly located to the donor segments whereas the LUMO is concentrated to the terminal nitro substituent which further verified that the charge distribution on such molecules is extremely influenced by NO_2_.

Table 5 signifies the difference between theoretical values of HOMO–LUMO band gap energy for 8a–e DSBs in gas phase and solvent media. The calculated electrochemical band gap energies of the 8a–e derivatives in gas phase are found in the range of 2.41–2.85 eV. The band gap energies were estimated to be in the order of 8b < 8e < 8a < 8c < 8d in gas phase. Overall, the band gap of DSBs decreases in selected solvents of varying polarities. As the electric permittivity of the solvents declines along the series DMF (*ε* = 37.22), THF (*ε* = 7.43), CHCl_3_ (*ε* = 4.71), toluene (2.37), and dioxane (*ε* = 2.21), the band gap energies of all DSBs increase, respectively. The results clearly reveal that the electron-donating ability of donor moieties in these compounds leads to the changing of band gap energy.

**Table 5:** The band gap energies (eV) obtained in gas phase and different solvents for 8a–e compounds

Compound	8a	8b	8c	8d	8e
Gas	2.48	2.41	2.46	2.85	2.42
DMF	2.11	2.05	2.11	2.52	2.06
CHCl_3_	2.20	2.13	2.20	2.62	2.15
THF	2.16	2.10	2.17	2.58	2.12
Dioxane	2.30	2.23	2.31	2.72	2.25
Toluene	2.30	2.22	2.30	2.70	2.23

The absorption (*λ*_ab_) and emission (*λ*_em_) wavelengths, the oscillator strength, and main assignments of 8a–e molecules in a variety of solvents were predicted from TD-DFT calculations and listed in Table 6. For instance, the electronic absorption band with the highest wavelength of 8c compound has been determined at 395.1 nm in DMF, 394.5 nm in CHCl_3_, 394.6 nm in THF, 392.3 nm in dioxane, and 393.3 nm in toluene solvent. In line with experimental UV-Vis spectra, the *λ*_ab_ of 8d compound is less than others. The electronic absorption of 8a–e derivatives essentially originates from HOMO − 1 → LUMO transition. As obtained for 8a–e series, the experimental and calculated maximum absorption values follow a similar trend while some deviations (∼9–11%) from the experimental values are observed. This deviation could be expected from the bulk solvent effects in experimental conditions while the calculated data are obtained by considering implicit solvent models.

**Table 6:** Theoretical electronic transition parameters, oscillator strengths and important contributions of the 8a–e compounds in the solvent media (TD-B3LYP/6-31+G(d,p))

Solvent	*λ*_ab_ (nm)	Osi. stren.	Major contributions	*λ*_em_ (nm)
8a				
DMF	397.6	0.957	H − 1 → LUMO (84%)	514.1
CHCl_3_	394.6	1.042	H − 1 → LUMO (78%)	486.8
THF	396.7	1.009	H − 1 → LUMO (81%)	502.1
Dioxane	393.5	1.134	H − 1 → LUMO (68%)	463.1
Toluene	394.5	1.118	H − 1 → LUMO (70%)	465.9

8b				
DMF	398.1	0.985	H − 1 → LUMO (85%)	502.7
CHCl_3_	396.3	1.066	H − 1 → LUMO (79%)	495.9
THF	396.4	1.036	H − 1 → LUMO (82%)	505.6
Dioxane	392.2	1.156	H − 1 → LUMO (71%)	470.8
Toluene	394.3	1.140	H − 1 → LUMO (72%)	474.0

8c				
DMF	395.1	0.505	H − 1 → LUMO (86%)	519.5
CHCl_3_	394.5	0.670	H − 1 → LUMO (75%)	490.5
THF	394.6	0.780	H − 1 → LUMO (85%)	501.5
Dioxane	392.3	0.519	H − 1 → LUMO (64%)	479.5
Toluene	393.3	0.628	H − 1 → LUMO (74%)	482.3

8d				
DMF	361.6	0.891	H − 1 → LUMO (55%)	511.3
CHCl_3_	359.6	0.506	H − 1 → LUMO (52%)	487.2
THF	360.2	0.521	H − 1 → LUMO (44%)	508.2
Dioxane	357.4	0.585	H − 1 → LUMO (69%)	442.6
Toluene	358.0	0.589	H − 1 → LUMO (70%)	473.9

8e				
DMF	396.2	1.098	H − 1 → LUMO (77%)	503.5
CHCl_3_	394.8	1.040	H − 1 → LUMO (81%)	491.2
THF	395.1	1.012	H − 1 → LUMO (85%)	501.3
Dioxane	391.9	1.030	H − 1 → LUMO (62%)	456.3
Toluene	393.0	1.029	H − 1 → LUMO (72%)	467.8

## Conclusions

In conclusion we have developed an efficient palladium catalyst system based on MOFs in the synthesis of a very important class of fluorescence compounds, DSBs, using Heck chemistry. Using this synthetic methodology it is possible to synthesize different DSB derivatives in good to excellent yields. It seems that the MOF structure is effectively facilitate the Heck reaction between bis-alkenes or aryl halides homoselectivity in order to have mono-functionalized products in good yields. Mono-functionalized products in the both forms of vinyl- and halogen-functionalized stilbenes are important in the synthesis of unsymmetrical DSB derivatives which open our hands to have D–π–A systems. Using Pd–NHC–MIL-101(Cr) catalyst it is possible to synthesis both symmetrical and unsymmetrical DSBs in high yields. Some new D–π–A DSBs which are containing different amino groups (D group) and nitro group (A group) were synthesized successfully using this new synthetic methodology in high isolated yields. The photophysical properties of these fluorescence compounds were investigated and DFT calculations were accomplished to investigate the optimized molecular geometry, band gap energy, and the electronic absorption and emission wavelengths.

## Conflicts of interest

There are no conflicts to declare.

## Supplementary Material

RA-011-D1RA00457C-s001

## References

[cit1] Wang Y., Liu W., Ye S., Zhang Q., Duan Y., Guo R., Wang L. (2020). J. Mater. Chem. C.

[cit2] Xu Y., Liang X., Liang Y., Guo X., Hanif M., Zhou J., Zhou X., Wang C., Yao J., Zhao R., Hu D., Qiao X., Ma D., Ma Y. (2019). ACS Appl. Mater. Interfaces.

[cit3] Lee C. H., Choi S. H., Oh S. J., Lee J. H., Shim J. W., Adachi C., Lee S. Y. (2020). RSC Adv..

[cit4] Pathak S. K., Xiang Y., Huang M., Huang T., Cao X., Liu H., Xie G., Yang C. (2020). RSC Adv..

[cit5] Kok C., Doyranli C., Canımkurbey B., Mucur S. P., Koyuncu S. (2020). RSC Adv..

[cit6] Yao C., Liu B., Zhu Y., Hong L., Miao J., Hou J., He F., Meng H. (2019). J. Mater. Chem. A.

[cit7] Du X., Yuan Y., Zhou L., Lin H., Zheng C., Luo J., Chen Z., Tao S., Liao L.-S. (2020). Adv. Funct. Mater..

[cit8] Tisovský P., Gáplovský A., Gmucová K., Novota M., Pavúk M., Weis M. (2019). Org. Electron..

[cit9] Koli M. R., Labiod A., Chakraborty S., Kumar M., Lévêque P., Ulrich G., Leclerc N., Jacquemin D., Mula S. (2020). ChemPhotoChem.

[cit10] De Acha N., Elosúa C., Corres J. M., Arregui F. J. (2019). Sensors.

[cit11] Ma J., Wang Y., Liu G., Xu N., Wang X. (2020). RSC Adv..

[cit12] Tsumura S., Ohira K., Imato K., Ooyama Y. (2020). RSC Adv..

[cit13] Aydin D. (2020). Talanta.

[cit14] Long L., Han Y., Yuan X., Cao S., Liu W., Chen Q., Wang K., Han Z. (2020). Food Chem..

[cit15] Kwon H.-Y., Liu X., Choi E. G., Lee J. Y., Choi S.-Y., Kim J.-Y., Wang L., Park S.-J., Kim B., Lee Y.-A., Kim J.-J., Kang N. Y., Chang Y.-T. (2019). Angew. Chem., Int. Ed..

[cit16] Chen W., Matsunaga T., Neill D. L., Yang C., Akaike T., Xian M. (2019). Angew. Chem..

[cit17] Mishra S., Awasthi P., Singh J., Gupta R. K., Singh V., Kant R., Jeet R., Goswami D., Goel A. (2018). J. Org. Chem..

[cit18] Chung H. Y., Oh J., Park J.-H., Cho I., Yoon W. S., Kwon J. E., Kim D., Park S. Y. (2020). J. Phys. Chem. C.

[cit19] Park I.-H., Chu L., Leng K., Choy Y. F., Liu W., Abdelwahab I., Zhu Z., Ma Z., Chen W., Xu Q.-H., Eda G., Loh K. P. (2019). Adv. Funct. Mater..

[cit20] Granados A., Vallribera A. (2019). Dyes Pigm..

[cit21] Gopinath A., Ramamurthy K., Subaraja M., Selvaraju C., Nasar A. S. (2018). New J. Chem..

[cit22] Zamani E., Yahyaei H., Khosravi A., Mohseni M., Shaki H. (2019). J. Macromol. Sci., Part B: Phys..

[cit23] Pilehkouhi M., Shaki H., Khosravi A., Khorasani M., Zamani E. (2018). J. Macromol. Sci., Part B: Phys..

[cit24] Zamani E., Shaki H., Rafizadeh M., Khosravi A., Pilehkouhi M. (2017). Fibers Polym..

[cit25] Li Z., Huang B., Wang Y., Yuan W., Wu Y., Yu R., Xing G., Zou T., Tao Y. (2020). RSC Adv..

[cit26] Pazin W. M., Almeida A. K. A., Manzoni V., Dias J. M. M., de Abreu A. C. F., Navarro M., Ito A. S., Ribeiro A. S., de Oliveira I. N. (2020). RSC Adv..

[cit27] Ji G., Wang N., Yin X., Chen P. (2020). Org. Lett..

[cit28] Gao F., Yang L., Yang L., Li H., Zhang S. (2010). J. Fluoresc..

[cit29] Mukherjee S., Pal P., Maity D., Baitalik S. (2019). J. Photochem. Photobiol., A.

[cit30] Łukasik B., Milczarek J., Pawlowska R., Żurawiński R., Chworos A. (2017). New J. Chem..

[cit31] Shi J., Izquierdo M. A., Oh S., Park S. Y., Milián-Medina B., Roca-Sanjuán D., Gierschner J. (2019). Org. Chem. Front..

[cit32] Harrowven D. C., Guy I. L., Howell M., Packham G. (2006). Synlett.

[cit33] Khan Z. A., Iqbal A., Shahzad S. A. (2017). Mol. Diversity.

[cit34] Meier H., Kim S., Oehlhof A. (2009). Synthesis.

[cit35] Szukalski A., Parafiniuk K., Haupa K., Goldeman W., Sahraoui B., Kajzar F., Mysliwiec J. (2017). Dyes Pigm..

[cit36] Tyagi V., Fasan R. (2016). Angew. Chem..

[cit37] Karbach A., Stemler T., Kopp C., Trommer W. E. (2014). Synthesis.

[cit38] Singh M., Argade N. P. (2012). Synthesis.

[cit39] Rau H. H., Werner N. S. (2018). Bioorg. Med. Chem. Lett..

[cit40] Rameau N., Russo B., Mangematin S., Pinel C., Djakovitch L. (2018). Appl. Catal., A.

[cit41] Traficante C. I., Fagundez C., Serra G. L., Mata E. G., Delpiccolo C. M. L. (2016). ACS Comb. Sci..

[cit42] Girase T. R., Kapdi A. R. (2019). Chem.–Asian J..

[cit43] Demidoff F. C., de Souza F. P., Netto C. D. (2017). Synthesis.

[cit44] Skhiri A., Salem R. B., Soulé J.-F., Doucet H. (2016). Synthesis.

[cit45] Mahmoodi A., Panahi F., Eshghi F., Kimiaei E. (2018). J. Lumin..

[cit46] Miri F. S., Gorji Kandi S., Panahi F. (2020). J. Fluoresc..

[cit47] Karimi-Alavijeh H., Panahi F., Gharavi A. (2014). J. Appl. Phys..

[cit48] Sharbati M. T., Panahi F., Nekoei A.-R., Emami F., Niknam K. (2014). J. Photonics Energy.

[cit49] Sharbati M. T., Panahi F., Gharavi A. (2010). IEEE Photonics Technol. Lett..

[cit50] Panahi F., Mahmoodi A., Ghodrati S., Eshghi F. (2020). RSC Adv..

[cit51] Niknam E., Panahi F., Khalafi-Nezhad A. (2020). Appl. Organomet. Chem..

[cit52] Balinge K. R., Bhagat P. R. (2017). C. R. Chim..

[cit53] Tang Y.-Q., Lu J.-M., Shao L.-X. (2011). J. Organomet. Chem..

[cit54] Astakhov A. V., Khazipov O. V., Chernenko A. Y., Pasyukov D. V., Kashin A. S., Gordeev E. G., Khrustalev V. N., Chernyshev V. M., Ananikov V. P. (2017). Organometallics.

[cit55] Sreenivasulu M., Kumar K. S., Kumar P. R., Chandrasekhar K. B., Pal M. (2012). Org. Biomol. Chem..

[cit56] Gottumukkal A. L., de Vries J. G., Minnaard A. J. (2011). Chem.–Eur. J..

[cit57] Taige M. A., Zeller A., Ahrens S., Goutal S., Herdtweck E., Strassner T. (2007). J. Organomet. Chem..

[cit58] The ICP analysis of Pd–NHC–MIL-101(Cr) catalyst shows that it contains 1.3 mmol g^−1^ of Pd

[cit59] Bhavania S., Ashfaq M. A., Rambabu D., Rao M. V. B., Pal M. (2019). Arabian J. Chem..

[cit60] Perosa A., Tundo P., Selva M., Zinovyeva S., Testa A. (2004). Org. Biomol. Chem..

[cit61] Khler K., Heidenreich R. G., Krauter J. G. E., Pietsch J. (2002). Chem.–Eur. J..

[cit62] Zhou X.-Y., Chen X., Wang L.-G. (2017). Synthesis.

[cit63] Zolfigol M. A., Amani K., Ghorbani-Choghamarani A., Hajjami M., Ayazi-Nasrabadi R., Jafari S. (2008). Catal. Commun..

[cit64] Kumpf J., Freudenberga J., Bunz U. H. F. (2015). Analyst.

[cit65] Kasun Z. A., Sato H., Nie J., Mori Y., Bender J. A., Roberts S. T., Krische M. J. (2018). Chem. Sci..

[cit66] Zhang J., Konsmo A., Sandberg A., Wu X., Nyström S., Obermüller U., Wegenast-Braun B. M., Konradsson P., Lindgren M., Hammarström P. (2019). J. Med. Chem..

[cit67] Park I.-H., Sasaki K., Quah H. S., Lee E., Ohba M., Lee S. S., Vittal J. J. (2019). Cryst. Growth Des..

[cit68] Suen M., Hang L., Lee W., Chan A. S. C., Kwong F. Y. (2008). Tetrahedron Lett..

[cit69] Paul A., Biswas A., Sinha S., Shah S. S., Bera M., Mandal M., Singh N. D. P. (2019). Org. Lett..

[cit70] Gopinath A., Ramamurthy K., Subaraja M., Selvaraju C., Nasar A. S. (2018). New J. Chem..

[cit71] Mishra S., Awasthi P., Singh J., Gupta R. K., Singh V., Kant R., Jeet R., Goswami D., Goel A. (2018). J. Org. Chem..

