# Supplementary material for: Synthesis of some new distyrylbenzene derivatives using immobilized Pd on an NHC-functionalized MIL-101(Cr) catalyst: photophysical property evaluation, DFT and TD-DFT calculations
Source: RSC Adv. 2021 Mar 29;11(20):12374–80. doi: 10.1039/d1ra00457c (PMC8696979; doi:10.1039/d1ra00457c)
Supplement: RA-011-D1RA00457C-s001 [file RA-011-D1RA00457C-s001.pdf]

Electronic Supplementary Material (ESI) for RSC Advances.
This journal is © The Royal Society of Chemistry 2021

Synthesis of Some New Distyrylbenzene Derivatives Using Immobilized Pd on a NHC-Functionalized MIL-101(Cr) Catalyst: Photophysical Properties Evaluation, DFT and TD-DFT Calculations

Esmaeil Niknam,[a] Ali Mahmmodi, [b] Farhad Panahi,*[a] Maryam Heydari Dokoohaki,[a] Aminreza Zolghadr[a] and Ali Khalafi-Nezhad*[a]

[a] Department of Chemistry, College of Sciences, Shiraz University, Shiraz 71454, Iran.

[b] Department of Polymer Engineering and Color Technology, Amirkabir University of Technology, Tehran, Iran.

* indicates the main/corresponding author.

panahi@shirazu.ac.ir; khalafi@chem.susc.ac.ir

Outline

1. Experimental and Spectral data S2

2. Fluorescence data S12

3. Copy of ^{1}HNMR and ^{13}CNMR of synthesized compounds S17

1. Experimental

1.1. General

Chemicals were purchased from Fluka, Merck and Aldrich companies and used without further purification. ^{1}H (400 MHz) and ^{13}C NMR (100 MHz) spectra were recorded on a Brucker AVANCE DRX in deutrated solvents. FT-IR spectroscopy (Shimadzu FT-IR 8300 spectrophotometer) was employed for characterization of the products. Melting points were determined in open capillary tubes in a Buchi melting point B-545.The photoluminescence (PL) spectra were obtained by excitation at the absorption maxima and recorded with a Varian Cary Eclipse Agilent Spectrofluorometer. The UV–Vis spectra were obtained with a UV-1280 Shimadzu Spectrophotometer. The reaction monitoring was accomplished by TLC on silica gel PolyGram SILG/UV254 plates. Column chromatography was carried out on columns of silica gel 60 (70−230 mesh) meshes.

The DFT and TD-DFT calculations were performed using Gaussian 09 quantum chemical program package.[REF:M. J. Frisch, G. W. Trucks, H. B. Schlegel, G. E. Scuseria, M. A. Robb, J. R. Cheeseman, G. Scalmani, V. Barone, B. Mennucci and G. A. Petersson, et al. Gaussian 09 Gaussian Inc.: Wallingford, CT, USA, 2009.] The ground state configurations of 8a-8e DSBs compounds were fully optimized using hybrid functional B3LYP with basis set 6-31+G(d,p) for all atoms in the gas phase. The geometries of the DBSs in solvents of different polarities were also optimized using the same functional in combination with polarizable continuum model (PCM) as implemented in Gaussian 09.[1] The solvents used were toluene, tetrahydrofuran (THF), 1,4-dioxane (dioxane), chloroform ($CHCl_3$), and *N,N*-dimethyl formamide (DMF). The vibrational analysis was computed using the same method to verify the local minima on the energy surface of studied structures. The same optimized geometries of compounds in solvent environments subjected to evaluate the electronic spectra (absorption and emission spectra) and their corresponding oscillator strengths using TD-DFT computations at the same level of theory.

1. J. Tomasi, B. Mennucci, R. Cammi, *Chem. Rev.*, **2005**, *105*, 2999.

All of the solutions containing compounds (**8a-8e**) were prepared at concentration of 10^{-5} M in THF, Chloroform, Toluene, Dioxane and DMF solvents. Emission and absorption spectra were taken by Varian Cary Eclipse Agilent Spectrofluorometer and UV-1280 Shimadzu Spectrophotometer under natural daylight simulator (D65) lamps (top image), and irradiation of A-Class UV lamps (bottom image). Digital photographs of the solutions were taken by digital camera.

1.2. Synthesis of MIL-101- NHC-Pd catalyst

1.2.1. General procedure for the Synthesis of MIL-101(Cr)

A mixture of Cr $(NO_3)_3.9H_2O$(4.5 g, 11 mmol), of terephthalic acid (10 mmol, 1.66 g), deionized water (45 mL), and hydrofluoric acid (0.6 mL of5M solution, 10 mmol) was charged in to the 75 mL capacity Teflon lined stainless steel autoclave. The mixture was sonicated 10 min, and placed in a preheated oven at 220˚C for 8 h. Afterward autoclave was allowed to cool down to room temperature filtered and washed with distilled water and dried in an oven at 80 ˚C for overnight. The crude product consists of green powder of MIL-101(Cr) along with white sharp needle-type crystals of unreacted terephthalic acid. The unreacted terephthalic acid was removed in two steps. In the first step the crude product was added to a 100 mL double-necked round bottom. Flask attached with a reflux condenser placed on a magnetic stirrer followed by addition of 50 mL of DMF. The mixture was refluxed at 130 ˚C for 12 h, filtered while hot, washed with hot DMF (2 x 25 mL) and dried. This process was repeated two times and finally the product was dried at 70˚C for 12 h. In the second step, the resulting green powder was added to 50 mL hot ethanol and refluxed overnight followed by hot filtration. Finally, the material was heated at 80 ˚C in an oven for 12 h to complete the activation.

1.2.2. General procedure for the Synthesis of MIL-101(Cr)-CH_2Cl

A mixture of MIL-101(Cr) (1.0 g), aluminum chloride hexahydrate (8 mmol, 1.9 g), methoxyacetyl chloride (4 mmol, 0.4 g) and nitromethane (80 mL) was charged in to the 100 mL three necked flask with a condenser. The reaction is carried out at 100 ºC with continuous stirring for 5 h. Then any solid product of chloromethylation was washed overnight with boiling, distilled water. Followed it was washed with boiling THF for 3 hours. The resulting MIL-101(Cr)-CH_2Cl product was dried in a furnace at 100˚C for 12 h as a light green solid (0.7 g).

1.2.3. General procedure for the Synthesis of MIL-101(Cr)-CH_2-IM

In this step the resultingMIL-101(Cr)-CH_2Cl was modified with *N*-methyl imidazole. In a typical procedure, *N*-methyl imidazole (0.4g, 5 mmol) and NaI (0.005g, 0.03 mmol) were added to a mixture of CM-MIL-101 (0.5 g) in CH_3CN (10 mL) and refluxed for 48 h at 80 ºC. Then, the solid was filtered off, washed with CH_3CN (3 x 5 mL), K_2CO_3/H_2O (5 mL, 1M), methanol (5 mL, diethylether (5 mL) and dried at 80˚C in vacuum for 12 h. The prepared MIL-101(Cr)-CH_2-IMwas obtained as a light gray solid (0.5 g).

1.2.4. General procedure for the Synthesis of MIL-101- NHC-Pd catalyst

The prepared MIL-101(Cr)-CH_2-IM was used as support for immobilization of Pd. The final catalyst was obtained by adding $Pd(OAc)_2$ (100 mg, 0.45 mmol) to a dispersed mixture of MIL-101(Cr)-CH_2-IM(250 mg) in toluene (20 mL) and keeping it under nitrogen atmosphere at 100 ˚C for 12 h. The resulting dark gray complex was collected by filtration and washed with ethanol (2× 10 mL) to remove the unreacted starting materials followed by drying under air.

1,4-bis((E)-4-nitrostyryl)benzene (3a).

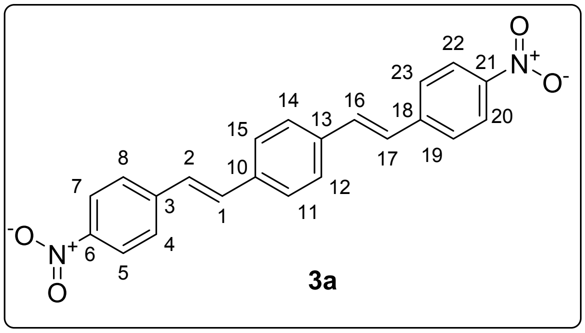

Into a conical flask (10 mL) a mixture of 4-bromo nitrobenzene (1 mmol, 0.20 g), divenylbenzene (0.55 mmol, 0.07 g), K_2CO_3 (2 mmol, 0.28 g), Pd-NHC-MIL101 (Cr) catalyst (12.0 mg, 1.5 mol %) and DMF (5 mL) were stirred at 110 °C. The reaction was monitored by TLC. The reaction mixture was stirred for 12 h. After completion of the reaction, the mixture was filtered and cooled down to room temperature and then added 25 mL water. The organic compound was precipitated, then washed with water and dried at 75-80 °C. The crude product was purified by column chromatography, eluting with *n*-hexane/ EtOAc 20: 2 (v/v), to afford the title compound.

Yield: 86%.

Yellow solid (M.p.185 °C).

^{1}H NMR (250 MHz, CDCl$_3$): δ (ppm) = 8.40 (d, *J* = 5.0 Hz, 4 H, C_5, C_7, C_{20}, C_{22}-H), 8.28(d, *J* = 5.0 Hz, 2 H, C_1, C_{16}-H), 7.83(d, *J* = 5.0 Hz, 4 H, C_4, C_8, C_{19}, C_{23}-H), 7.70 (d, *J* = 5.0 Hz, 2H, C_2, C_7 -H), 7.32-7.27(m, 4H, C_{11}, $C_{12,}$ $C_{14,}$ C_{15} -H).
^{13}C NMR (100 MHz, CDCl$_3$): δ (ppm) = 148.0 (C_6 or C_{21}), 146.9 (C_3 or C_{18}), 145.0 (C_6 or C_{21}), 143.5 (C_3 or C_{18}), 136.9 (C_{10} or C_{13}), 132.7 (C_{10} or C_{13}), 129.4, 128.3, 127.5, 127.2, 127.0, 126.9, 125.7, 124.4, 124.2 (other aromatic carbons and vinylic carbons).
Anal. Calc. for $C_{22}H_{16}N_2O_4$: C, 70.96; H, 4.33; N, 7.52; O, 17.19.Found: C 70.87; H, 4.24; N, 7.58.

(E)-1-nitro-4-(4-vinylstyryl)benzene (3a').

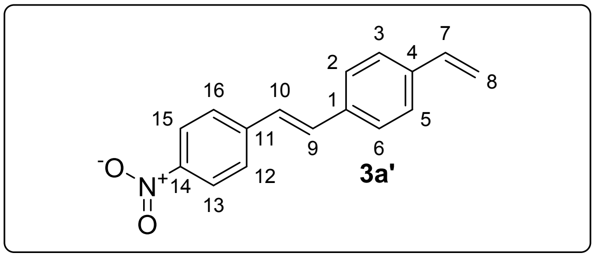

Into a conical flask (10 mL) a mixture of 4-bromonitrobenzene (1.0 mmol, 0.20 g), 1,4-divenylbenzene (1.1 mmol, 0.14 g), K_2CO_3 (2.0 mmol, 0.28 g), Pd-NHC-MIL101(Cr) catalyst (12.0 mg, 1.5 mol %) and DMF (10 mL) heated in an oil bath at 110 °C for 12 h. The reaction was followed by TLC. After completion of the reaction the mixture was cooled down to room temperature, and then water (10 mL) was added. The organic layer was extracted with ethyl acetate (3 x 10 mL) from the aqueous layer and dried over anhydrous Na_2SO_4, filtered, and concentrated in vacuum. The crude product was purified by column chromatography, eluting with *n*-hexane/EtOAc 20:1 (v/v), to afford the title compound.
Yield: 86%.
Orange solid (M.p.146-148 °C).
IR (KBr) ν: 3023, 1591, 1506, 1336, 1184, 1109, 968, 832, 797, 691 cm^{-1}.
^{1}H NMR (250 MHz, CDCl$_3$): δ(ppm) = 8.20 (d, *J* = 8.75 Hz, 2H, C_{15}, C_{13}-H), 7.60 (d, *J* = 8.75 Hz, 2H, C_{12}, C_{16}-H), 7.47 (dd, *J* = 18.5 Hz, *J* = 8.5 Hz, 4H, $C_{2,}$ $C_{3,}$ $C_{5,}$ C_6-H), 7.17 (dd, *J* = 16.25 Hz, *J* = 16.25 Hz, 2H, C_9,C_{10}-H), 6.74 (dd, *J* = 17.5 Hz, *J* = 10.75 Hz, 1H, C_7-H), 5.81 (dd, *J* = 17.5, *J* = 0.7 Hz, 1H, C_8-H *trans*), 5.32 (d, *J*= 10.75 Hz, 1H, C_9-H *cis*).

^{13}C-NMR (100 MHz, $CDCl_3$): δ (ppm) = 146.7 (C_{14}), 143.8 (C_{11}), 138.1(C_4), 136.2 (C_1), 135.7, 133.3, 132.9, 128.4, 127.3, 126.8, 126.7, 126.1, 125.3 (aromatic carbons and vinylic carbons), 124.2 (C_9, C_{10}), 114.6 (C_8).

Anal. Calcd for $C_{16}H_{13}NO_2$ (251.29): C, 76.48; H, 5.21; N, 5.57; O, 12.73. Found: C, 76.41; H, 5.14; N, 5.63.

4,4'-((1E,1'E)-1,4-phenylenebis(ethene-2,1-diyl))dibenzaldehyde (3b).

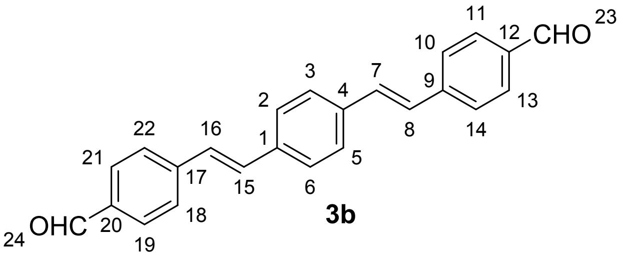

3b

Into a conical flask (10 mL) a mixture of 4-bromobenzaldehyde (1 mmol, 0.18 g), divenylbenzene (0.55 mmol, 0.07 g), K_2CO_3 (2 mmol,0.28 g), Pd-NHC-MIL101 (Cr) catalyst(12.0 mg, 1.5 mol %) and DMF (5 mL) were stirred at 110 °C. The reaction was monitored by TLC. The reaction mixture was stirred for 12 h. After completion of the reaction, the mixture was filtered and cooled down to room temperature and then added 25mL water. The organic compound was precipitated, then washed with water and dried at 75-80°C. The crude product was purified by column chromatography, eluting with *n*-hexane/EtOAc 20:5(v/v), to afford the title compound.

Yield: 85%.

Yellow solid (M.p.149-151°C).

IR (KBr) ν: 3025, 2836, 1688, 1597, 1400, 1304, 1211, 1166, 1107, 969, 860, 817 cm^{-1}.

^{1}H NMR (250 MHz, $CDCl_3$): δ (ppm) = 10.01 (s, 2 H, C_{23}, C_{24}-H), 7.90 (d, *J* = 7.5 Hz, 4 H, C_{11}, C_{13}, C_{19}, C_{21}-H), 7.70 (d, *J* = 7.5 Hz, 4 H, C_{10}, C_{14}, C_{18}, C_{22}-H), 7.19-7.58 (m, 8 H, C_2, C_3, C_5, C_6, C_7, C_8, C_{15}, C_{16}-H).

^{13}C NMR (100 MHz, $CDCl_3$): δ (ppm) = 191.7, 191.6 (C_{23}, C_{24}), 143.2, 137.1 (C_9, C_{17}), 135.9, 135.4 (C_1, C_4), 131.7, 130.4, 130.3, 129.3, 128.0, 127.9, 127.0, 126.8, 125.6 (other aromatic and vinilic carbons).

Anal. Calcd for $C_{24}H_{18}O_2$: C, 85.18; H, 5.36; O, 9.46.Found: C 85.21; H, 5.31.

(E)-4-(4-vinylstyryl)benzaldehyde(3b').

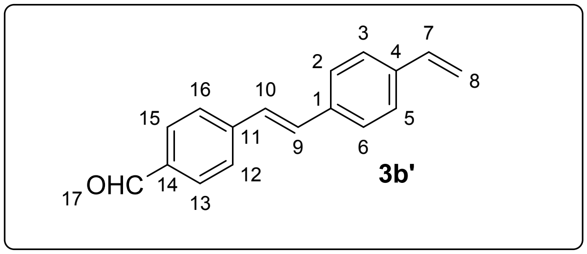

Into a conical flask (10 mL) a mixture of 4-bromo benzaldehyde (1 mmol, 0.18 g), 1,4-divenylbenzene(1.1mmol, 0.14 g), K_2CO_3 (2 mmol, 0.28 g), Pd-NHC-MIL101(Cr) catalyst (12.0 mg, 1.5 mol %) and DMF (5 mL) were stirred at 110 °C. The reaction was monitored by TLC. The reaction mixture was stirred for 12 h. After completion of the reaction, the mixture was filtered and cooled down to room temperature and then added water. The organic compound was extracted with ethyl acetate (3 x 5 mL) from the aqueous layer and dried over anhydrous Na_2SO_4, then filtered and concentrated in vacuum. The crude product was purified by column chromatography, eluting with *n*-hexane/EtOAc 20:3(v/v), to afford the title compound.

Yield: 80 %.

Yellow solid (M.p.97-96°C).

IR (KBr) ν: 3024, 2965, 2931, 2825, 2735, 1697, 1595, 1422, 1304, 1211, 1166, 971, 831, 819, 695cm^{-1}.

^{1}H NMR (250 MHz, $CDCl_3$): δ (ppm) = 9.99 (s, 1 H, C_{17}-H), 7.86(d, *J* = 7.5 Hz, 2 H, C_{13}, C_{15}-H), 7.64(d, *J* = 7.5 Hz, 2 H, C_{12}, C_{16}-H), 7.47 (d, *J* = 7.5 Hz, 2 H, C_2,C_6-H or C_3, C_5-H), 7.29-7.06 (m, 7 H, other aromatic and vinilic carbons).

^{13}C NMR (100 MHz, $CDCl_3$): δ (ppm) = 191.7 (C_{17}), 145.0 (C_{11}), 143.7 (C_{14}), 135.1, 134.0, 132.2, 130.2, 130.0, 128.4, 126.9, 126.9, 126.8, 126.4 (other aromatic carbons and vinylic carbons).

Anal. Calcd for $C_{16}H_{14}O$: C, 82.94; H, 6.89; N, 3.45; O, 7.98. Found: C82.83; H, 6.78; N, 3.36.

4,4'-((1E,1'E)-1,4-phenylenebis(ethene-2,1-diyl))dibenzoic acid(3c).

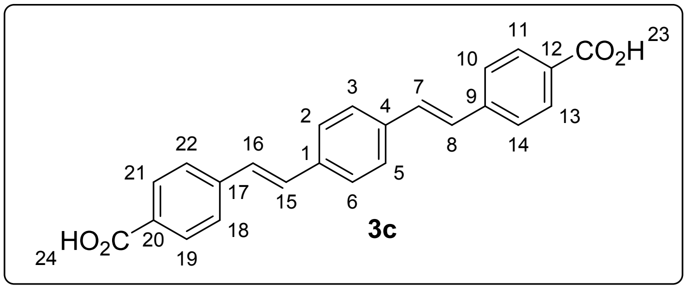

Into a conical flask (10 mL) a mixture of 4-bromo benzoic acid (1 mmol, 0.20 g), 1,4-divenylbenzene (0.55 mmol, 0.07 g), triethylamine (2 mmol, 0.20 g), Pd-NHC-MIL101(Cr) catalyst (12.0 mg, 1.5 mol %) and DMF (5 mL) were stirred at 110 °C. The reaction was monitored by TLC. The reaction mixture was stirred for 12 h. After completion of the reaction, the mixture cooled down to room temperature and acidified with about 0.5 mL of HCl (20 %) then added 25 mL water. The organic compound was precipitated, washed with hot DMF (3 x 5 mL) and water then dried at 100 °C.

Yield: 76 %.

Yellow solid (M.p.175-178 °C).

IR (KBr) ν: 2400-3400, 1674, 1604, 1419,1288,1180,1103, 948, 848, 779, 694 cm^{-1}.

Anal. Calcd for $C_{24}H_{18}O_4$: C, 77.82; H, 4.90; O, 17.28. Found: C 77.69; H, 4.93.

4,4'-((1E,1'E)-1,4-phenylenebis(ethene-2,1-diyl))diphenol(3d).

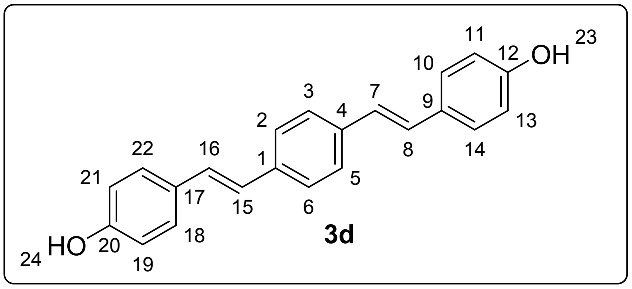

Into a conical flask (10 mL) a mixture of 4-bromo phenol (1 mmol, 0.17 g), 1,4-divenylbenzene (0.55 mmol, 0.07 g), K_2CO_3 (2 mmol, 0.28 g), Pd-NHC-MIL101(Cr) catalyst (12.0 mg, 1.5 mol %) and DMF (5 mL) were stirred at 110 °C. The reaction was monitored by TLC. The reaction mixture was stirred for 12 h. After completion of the reaction, the mixture was filtered and cooled down to room temperature and then added 25 mL water. The organic compound was precipitated, then washed with water and dried at 75-80 °C. The crude product was purified by column chromatography, eluting with *n*-hexane/EtOAc/MeOH 20:3:2 (v/v), to afford the title compound.

Yield: 78 %.

Yellow solid (M.p.218-220°C).

IR (KBr) ν:3301.9, 3016, 2923, 1589, 1512, 1458, 1380, 1242, 1172, 1103, 964, 817, 686.6cm^{-1}.

^{1}H NMR (250 MHz, Acetone-d6): δ (ppm) = 9.57 (s, 2 H, OH), 7.51-7.22 (m, 8H, other aromatic or vinylic protons), 7.15-7.08 (m, 2H, other aromatic or vinylic protons), 6.99 (d, d, *J* = 17.5 Hz, 2H, C_7-H or C_8-H or C_{15}-H or C-$_{16}$-H), 6-74-6.72 (m, 4H, other aromatic or vinylic protons).

^{13}C NMR (100 MHz, DMSO-d6): δ (ppm) = 157.8 (C_{12}, C_{20}), 138.3 (C_1, C_4), 129.4, 129.1, 128.2, 125.5, 124.0 (C_3, C_5, C6-C_{10}, C_{15}-C_{18}, C_{22}, C_{23}), 116.0 (C_{11}, C_{13}, C_{19}, C_{21}).

Anal. Calcd for $C_{22}H_{18}O_2$: C, 84.05; H, 5.77; O, 10.77.Found: C 83.94; H, 5.68.

4-(4-vinylstyryl) phenol (3d‘).

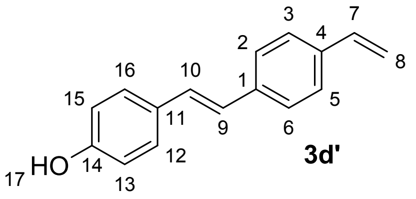

Into a conical flask (10 mL) a mixture of 4-bromophenol (1 mmol, 0.17 g), 1, 4-divenylbenzene (1.1 mmol, 0.14 g), K_2CO_3 (2 mmol, 0.28 g), Pd-NHC-MIL101 (Cr) catalyst (12.0 mg, 1.5 mol %) and DMF (5 mL) were stirred at 110 °C. The reaction was monitored by TLC. The reaction mixture was stirred for 12 h. After completion of the reaction, the mixture was filtered and cooled down to room temperature and then added water. The organic compound was extracted with ethyl acetate (3 x 5 mL) from the aqueous layer and dried over anhydrous Na_2SO_4, then filtered and concentrated in vacuum. The crude product was purified by column chromatography, eluting with *n*-hexane/EtOAc/MeOH 20:2:1 (v/v), to afford the title compound.

Yield: 74%.

Yellow solid (M.p.161-164°C).

IR (KBr) ν: 3278, 3024, 2962, 1596, 1512, 1458, 1388, 1249, 1103, 964, 833, 694 cm^{-1}.

^{1}H NMR (250 MHz, CDCl$_3$): δ (ppm) = 9.51 (s, 1 H, OH), 7.40-7.33 (m, 6 H, C_{12}, C_{16}, C_2, C_3-H), 6.88-7.13 (m, 5 H, C_7, C_8, C_9, C_{10}-H), 6.74-6.71 (d, J = 7.5 Hz, 2 H, C_{13}, C_{15}-H).

^{13}C NMR (100 MHz, CDCl$_3$): δ (ppm) = 157.8 (C_{14}), 157.6 (C_{14}), 144.4, 143.1, 138.0, 135.5 (C_1, C_4, C_{11}), 129.0, 128.7, 128.6, 128.5, 128.4, 128.3, 128.2, 127.9, 127.0, 126.5, 125.9, 125.7, 125.5, 124.0 (other aromatic and vinylic carbons), 116.0 (C_{13} or C_{15} or C_8).

.

Anal. Calcd for $C_{16}H_{14}O$: C, 86.45; H, 6.35; O, 7.20.Found: C 86.40; H, 6.27.

1,4-bis((E)-2-(pyridin-4-yl)vinyl)benzene (3e).

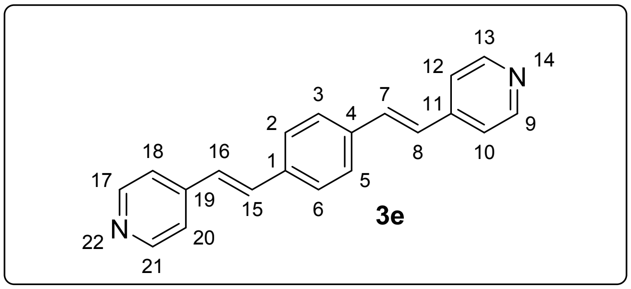

Into a conical flask (10 mL) a mixture of 1,4-dibromobenzene (1 mmol, 0.23 g), 4-vinyl pyridine (2 mmol, 0.21 g), K_2CO_3 (2 mmol, 0.28 g), Pd-NHC-MIL101(Cr) catalyst (12.0 mg, 1.5 mol%) and DMF (5 mL) were stirred at 110 °C. The reaction was monitored by TLC. The reaction mixture was stirred for 108 h. After completion of the reaction, the mixture was filtered and cooled down to room temperature and then added 25 mL water. The organic compound was precipitated, then washed with water and dried at 75-80 °C. The crude product was purified by column chromatography, eluting with *n*-hexane/EtOAc 20:8 (v/v), to afford the title compound.

Yield: 88 %.

Yellow solid (M.p. 202-205 °C).

IR (KBr) ν: 3024, 1635, 1589, 1488, 1411, 1072, 972, 825, 540 cm^{-1}.

^{1}H NMR (250 MHz, CDCl$_3$): δ (ppm) = 8.57 (d, J = 2.5 Hz, 4 H, C_9, C_{13}, C_{17}, C_{21}-H), 7.55-7.48 (m, 4H, C_{10}, C_{12}, C_{18}, C_{20}-H),7.40-7.32(m, 4 H, C_2, C_3, C_5, C_6-H), 7.26-7.18 (m, 2 H, C_8, C_{15}-H), 7.07- 6.94 (m, 2 H, C_7, C_{16}-H).

^{13}C NMR (75 MHz, CDCl$_3$): δ (ppm) = 150.27(C_9, C_{13}, C_{17}, C_{21}), 144.40, 144.22, 136.54, 135.08, 133.14, 132.42, 132.01, 131.86, 128.46, 127.52, 126.69, 126.49 (other aromatic and vinylic carbons), 122.69(C_7, C_{16}), 120.88(C_{18}, C_{20}, C_{10}, C_{12}).

Anal. Calcdfor $C_{20}H_{16}N_2$: C, 84.48; H, 5.67; N, 9.85. Found: C 84.37; H, 5.61; N, 10.01

4-(4-bromophenyl)morpholine (7a).

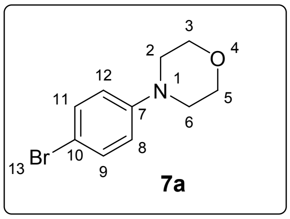

7a

Into a conical flask (10 mL) a mixture of 1, 4-dibromobenzene (1.0 mmol, 0.24 g), morpholine (1.0 mmol, 0.09 g), K_3PO_4 (2.0 mmol, 0.42 g), CuI (0.1 mmol, 0.02 g), PCA (0.2 mmol, 0.025 g), and DMF (5.0 mL) were stirred at 110 °C. The reaction was monitored by TLC. The reaction mixture was stirred for 12 h. After completion of the reaction, the mixture was cooled down to room temperature, and then 10 mL water was added. The organic compound was extracted with ethyl acetate (3 x 5 mL) from the aqueous layer and dried over anhydrous Na_2SO_4, filtered, and concentrated in vacuum. The crude product was purified by column chromatography, eluting with n-hexane/EtOAc 20:2 (v/v), to afford the title compound.

Yield: 74 %.

Pale yellow solid (M.p.115 °C).

IR (KBr) ν: 2963, 2856, 2826, 1589, 1491, 1332, 1233, 1117, 922, 818, 638 cm^{-1}.

^{1}H NMR (250 MHz, $CDCl_3$): δ (ppm) = 7.35 (d, J = 9 Hz, 2H, C_9, C_{11}-H), 6.77 (d, J = 9 Hz, 2H, C_8, C_{12}-H), 3.85 (d, J = 4.8 Hz, 2H, C_3, C_5-H), 3.12 (d, J = 4.8 Hz, 2H, C_2, C_6-H).

^{13}C NMR (62.5 MHz, $CDCl_3$): δ (ppm) = 150.3 (C_7), 132.0 (C_9, C_{11}), 117.3 (C_8, C_{12}), 112.2 (C_{10}), 66.8 (C_3, C_5), 49.1 (C_2, C_6).

Anal. Calcd for $C_{10}H_{12}BrNO$ (242.12): C, 49.61; H, 5.00; Br, 33.00; N, 5.79; O, 6.61. Found: C, 49.55; H, 5.08; N, 5.70.

1-(4-bromophenyl)piperidine (7b).

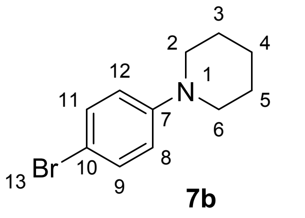

Into a conical flask (10 mL) a mixture of 1,4-dibromobenzene (1.0 mmol, 0.23 g), pipiridine (1.0 mmol, 0.085 g), K_3PO_4 (2 mmol, 0.42 g), CuI (0.1 mmol, 0.02 g), PCA (0.2 mmol, 0.025 g), and DMF (5 mL) were stirred at 110 °C. The reaction was monitored by TLC. The reaction mixture was stirred for 12 h. The workup process was same to compound **7a**. The crude product was purified by column chromatography, eluting with *n*-hexane/EtOAc 20:1 (v/v), to afford the title compound.

Yield: 76 %.

White solid (M.p.74 °C).

IR (KBr) ν: 3055, 2929, 2817, 1581, 1494, 1442, 1339, 1243, 1127, 915, 807 cm^{-1}.

^{1}H NMR (250 MHz, $CDCl_3$): δ (ppm) = 7.15 (d, *J* = 7.5 Hz, 2H, C_9, C_{11}-H), 6.61 (d, *J* = 7.5 Hz, 2H, C_8, C_{12}-H), 2.95 (t, *J* = 5.0 Hz, 4H, C_2, C_6-H), 1.56-1.42 (m, 6H, C_3, C_4, C_5-H).

^{13}C NMR (100 MHz, $CDCl_3$): δ (ppm) = 151.2 (C_7), 131.8 (C_9, C_{11}), 118.0 (C_8, C_{12}), 111.1 (C_{10}), 40.4 (C_2, C_6), 25.7 (C_3, C_5), 24.2 (C_4).

Anal. Calcd for $C_{11}H_{14}BrN$ (240.14): C, 55.02; H, 5.88; Br, 33.27; N, 5.83. Found: C, 54.93; H, 5.80; N, 5.86.

1-(4-bromophenyl)-4-phenylpiperazine (7c).

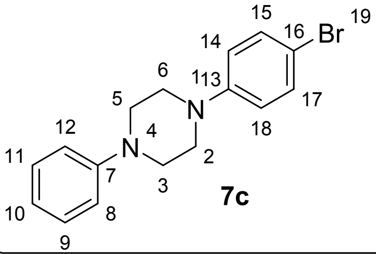

Into a conical flask (25 mL) a mixture of 1, 4-dibromobenzene (1.0 mmol, 0.23 g), *N*-phenylpiperazine (1.0 mmol, 0.16 g), K_3PO_4 (2.0 mmol, 0.42 g), CuI (0.1 mmol, 0.02 g), PCA (0.2 mmol, 0.025 g), and DMF (5 mL) were stirred at 110 °C. The reaction was monitored by TLC. The reaction mixture was stirred for 24 h. The workup process was same to compound **7a**. The crude product was purified by column chromatography, eluting with *n*-hexane/EtOAc 20:2 (v/v), to afford the title compound.

Yield: 71 %.

White solid (M.p. 160 °C).

IR (KBr) ν: 3036, 2959, 2880, 2829, 1587, 1491, 1448, 1326, 1226, 1154, 942, 814, 761, 693 cm^{-1}.

^{1}H NMR (400 MHz, $CDCl_3$): δ (ppm) = 7.37 (d, J = 9.2 Hz, 2H, C_9, C_{11}-H), 7.33-7.29 (m, 2H, C_{15}, C_{17}-H), 6.98 (d, J = 8.0 Hz, 2H,C_{15}, C_{17}-H), 6.93-6.89 (m, J= 8.0 Hz, 1H, C_{10}-H), 6.85 (d, J = 9.2 Hz, 2H, C_8, C_{12}-H), 3.32 (d, J = 3.2 Hz, 8H, C_2, C_3, C_5, C_6H).

^{13}C NMR (100 MHz, $CDCl_3$): δ (ppm) = 151.1(C_7), 150.2 (C_{13}), 131.9 (C_8, C_{12}), 129.2 (C_{15}, C_{17}), 120.2, 117.9, 116.4, 112.1(other aromatic carbons), 49.3(C_3, C_5 or C_2, C_6), 49.2(C_3, C_5 or C_2, C_6).

Anal. Calcd for $C_{16}H_{17}BrN_2$ (317.23): C, 60.58; H, 5.40; Br, 25.19; N, 8.83. Found: C, 60.61; H, 5.32; N, 8.88.

1-(4-bromophenyl)-*1H*-imidazole (7d).

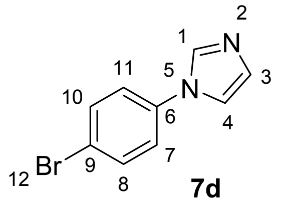

7d

Into a conical flask (10 mL) a mixture of 1, 4-dibromobenzene (1.0 mmol, 0.23 g), imidazole (1.0 mmol, 0.07 g), K_3PO_4 (2.0 mmol, 0.42 g), CuI (0.1 mmol, 0.02 g), PCA (0.2 mmol, 0.025 g), and DMF (5 mL) were stirred at 110 °C. The reaction was monitored by TLC. The reaction mixture was stirred for 12h.The workup process was same to compound **7a**.The crude product was purified by column chromatography, eluting with *n*-hexane:EtOAc:MeOH 20:2:1 (v/v), to afford the title compound.

Yield: 81 %.

White solid (M.p. 120 °C).

IR (KBr) ν: 3127, 3108, 1504, 1304, 1241, 1102, 1057, 959, 827, 786, 767, 661 cm^{-1}.

^{1}H NMR (250 MHz, $CDCl_3$): δ (ppm) = 7.81 (s, 1 H, C_1-H), 7.59-7.55 (m, 2H, C_8, C_{10}-H), 7.27- 7.19 (m, 4H, C_3, C_4, C_7, C_{11}-H).

^{13}C NMR (100 MHz, CDCl$_3$): δ (ppm) = 136.3 (C_6), 135.4 (C_9), 132.9 ($C_{8,}$ C_{10}), 130.8 (C_1), 122.9 (C_7, C_{11}), 120.8(C_3 or C_4), 1118.1(C_3 or C_4).

Anal. Calcd for $C_9H_7BrN_2$ (223.07): C, 48.46; H, 3.16; Br, 35.82; N, 12.56. Found: C, 48.53; H, 3.04; N, 12.66.

(4-bromophenyl)-L-alanine (7e).

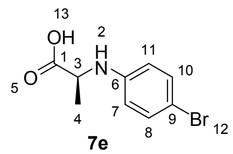

7e

Into a conical flask (10 mL) a mixture of 1,4-dibromobenzene (1.0mmol, 0.23 g), alanine (1.0 mmol, 0.09 g), K_3PO_4 (2.0 mmol, 0.42 g), CuI (0.1 mmol, 0.02 g), PCA (0.2 mmol, 0.025 g), and DMF (10.0 mL) were stirred at 110 °C. The reaction was monitored by TLC. The reaction mixture was stirred for 24 h. After completion of the reaction, the mixture was cooled down to room temperature, then water (10 mL) and ethyl acetate (10mL)were added. Under cooling with ice/water the solution of HCl (20%) was added to adjust the pH to 3. The organic layer was separated and the aqueous layer was extracted with ethyl acetate (3 x 5 mL). The organic separated layer was dried over anhydrous Na_2SO_4, filtered, and concentrated in vacuum. The crude product was purified by column chromatography, eluting with *n*-hexane/EtOAc 20:2 then *n*-hexane/EtOAc/MeOH 20:2:1 (v/v), to afford the title compound.

Yield: 68 %.

White solid (M.p.146-148°C).

IR (KBr) ν: 3425, 2989, 2974, 2881, 2794, 1583, 1490, 1387, 1363, 1112, 1089, 1073, 961, 850, 803, 682, 553 cm^{-1}.

^{1}H NMR (250 MHz, CDCl$_3$): δ (ppm) = 7.13 (d, *J*= 8.4 Hz, 2H, C_9, C_{10}-H), 6.40 (d, *J* = 8.4 Hz, 2H, C_7, C_{11}-H), 3.92 (q, *J* = 6.8 Hz, 1 H, C_3-H), 1.38 (d, *J* = 6.8 Hz, 3 H, C_4-H).

^{13}C NMR (100 MHz, CDCl$_3$): δ (ppm) = 176.1 (C=O), 145.9 (C_9), 131.8 (C_8, C_{10}), 114.8 (C_7, C_{11}), 109.2 (C_9), 51.6 (C_3), 18.6 (C_4).

Anal. Calcd for $C_9H_{10}BrNO_2$ (244.09): C, 44.29; H, 4.13; Br, 32.74; N, 5.74; O, 13.11. Found: C, 44.19; H, 4.26; N, 5.77.

4-(4-((E)-4-((E)-4-nitrostyryl)styryl)phenyl)morpholine (8a).

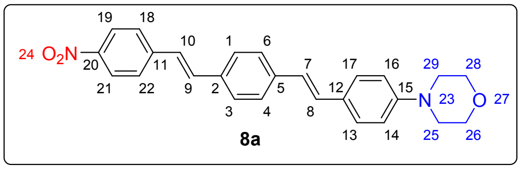

Into a conical flask (10 mL) a mixture of 4-(4-bromophenyl) morpholine (1.0 mmol, 0.24 g), (E)-1-nitro-4-(4-vinylstyryl) benzene (1.0 mmol, 0.25 g), K_2CO_3 (2.0 mmol, 0.28 g), NHC-Pd catalyst (12.0 mg, 1.5 mol %) and DMF (5 mL) heated in an oil bath at 110 °C for 12 h. The reaction was followed by TLC. After completion of the reaction the mixture was cooled down to room temperature, and then water (10 mL) was added. The organic layer was extracted with ethyl acetate (3 x 10 mL) from the aqueous layer and dried over anhydrous Na_2SO_4, filtered, and concentrated in vacuum. The crude product was purified by column chromatography, eluting with *n*-hexane/EtOAc 20:2→ 20:3 (v/v), to afford the title compound.

Yield: 83%.

Orange solid (M.p.155-158 °C).

^{1}H NMR (250 MHz, CDCl$_3$): δ (ppm) = 8.14 (d, J = 8.5 Hz, 2H, $C_{19,}$ C_{21}-H), 7.57-7.56 (m, 4H,C_{13}, C_{17}, C_{18}, C_{22}-H), 7.46-7.08 (m, 6 H, aromatic and vinylic protons), 6.96 d, J = 17.5 Hz, 2H, C_9, C_{10}-H), (6.83, d, J = 8.25 Hz, 2H, C_7, C_8-H), 3.79 (t, J = 4.25 Hz, 4H, C_{26}, C_{28}-H), 3.14 (t, J = 4.25 Hz, 4H, C_{25}, C_{29}-H).

^{13}C-NMR (100 MHz, CDCl$_3$): δ (ppm) = 150.9 (C_{20}), 146.8 (C_{15}), 143.9(C_{11}), 138.5(C_2), 136.5(C_5), 133.3, 129.1, 128.8, 127.7, 126.9, 126.6, 125.7, 125.4, 124.9 (other aromatic and vinylic carbons), 124.2 (C_{12}), 115.5(C_{14}, C_{16}), 66.9 (C_{26}, C_{28}), 48.9(C_{25}, C_{29}).

IR (KBr) ν: 3016, 2923, 2854, 1596, 1512, 1334, 1234, 1118, 964, 925, 817, 694 cm^{-1}.

Anal. Calcd for $C_{26}H_{24}N_2O_3$ (412.49): C, 75.71; H, 5.86; N, 6.79; O, 11.64. Found: C, 75.66; H, 5.87; N, 6.88.

1-(4-((E)-4-((E)-4-nitrostyryl)styryl)phenyl)piperidine (8b).

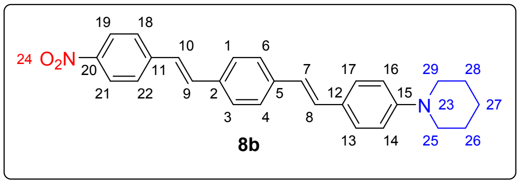

Into a conical flask (25 mL) a mixture of 1-(4-bromophenyl)piperidine (1.0 mmol, 0.24 g), (E)-1-nitro-4-(4-vinylstyryl)benzene(1.0 mmol, 0.25 g), K_2CO_3 (2.0 mmol, 0.28 g), NHC-Pd catalyst (12.0 mg, 1.5 mol %) and DMF (5 mL) heated in an oil bath at 110 °C for 12 h. The reaction was followed by TLC. The workup process was same to compound **8a**.The crude product was purified by column chromatography, eluting with *n*-hexane/EtOAc 20:1→ 20:2 (v/v), to afford the title compound.

Yield: 84%.

Reddish yellow to orange solid (M.p.156-160 °C).

IR (KBr) ν: 3010, 2931, 2854, 1596, 1512, 1334, 1234, 1110, 964, 810, 694 cm^{-1}.

^{1}H NMR (250 MHz, $CDCl_3$): δ (ppm) = 8.14 (d, *J* = 8.75 Hz, 2H, C_{19}, C_{21}-H), 7.46-6.82 (m, 14H, other aromatic and vinylic-H), 3.14 (t, *J* = 5.5 Hz, 4H), 1.63-1.42 (m, 6H).

^{13}C-NMR (100 MHz, $CDCl_3$): δ (ppm) = 151.8 (C_{15}), 146.8 (C_{20}), 143.9 (C_{11}), 138.7(C_2, C_5), 136.6, 136.5, 133.4, 129.4, 129.2, 127.6, 126.9, 126.4, 125.5, 124.9, 124.7(other aromatic and vinylic carbons), 124.2(C_{12}), 116.0 (C_{14}, C_{16}), 50.1 (C_{25}, C_{29}), 25.7(C_{26}, C_{28}), 24.4(C_{27}).

Anal. Calcd for $C_{27}H_{26}N_2O_2$ (410.52): C, 79.00; H, 6.38; N, 6.82; O, 7.79. Found: C, 78.94; H, 6.46; N, 6.83.

1-(4-((E)-4-((E)-4-nitrostyryl)styryl)phenyl)-4-phenylpiperazine (8c).

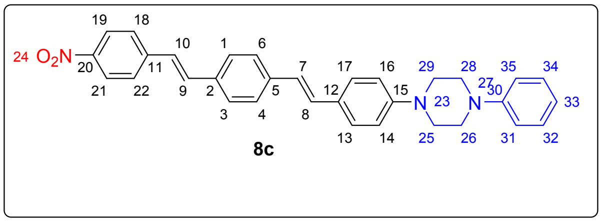

Into a conical flask (25 mL) a mixture of 1-(4-bromophenyl)-4-phenylpiperazine (1.0mmol, 0.32 g), (E)-1-nitro-4-(4-vinylstyryl) benzene(1.0 mmol, 0.25 g), K_2CO_3 (2.0 mmol, 0.28 g), NHC-Pd catalyst (12.0 mg, 1.5mol %) and DMF (5 mL) heated in an oil bath at 120 °C for 12 h. The reaction was followed by TLC. The workup process was same to compound **8a**.The crude product was purified by column

chromatography, eluting with *n*-hexane/ EtOAc 20:2→ 20:3 (v/v), to afford the title compound.

Yield: 80%.

Orange solid (M.p.134°C).

IR (KBr) ν: 3042, 2831, 1674, 1596, 1512, 1342, 1226, 1110, 964, 756, 694 cm^{-1}.

^{1}H NMR (400 MHz, $CDCl_3$): δ(ppm)= 8.14 (d, J = 8.8 Hz, 2H, C_{19}, C_{21}-H), 7.56 (d, J = 8.8 Hz, 2H, C_{18}, C_{22}-H), 7.46-7.29 (m, 7H, aromatic and vinylic-H), 7.22- 7.17 (m, 3H, aromatic and vinylic-H), 7.12- 7.02 (m, 3H, aromatic and vinylic-H), 6.94- 6.82 (m, 4H, C_{14}, C_{16}, C_{31}, C_{36}-H), 3.35-3.24(m, 8H, C_{25}, C_{26}, C_{28}, C_{29}-H).

^{13}C-NMR (100 MHz, $CDCl_3$): δ (ppm) = 155.8(C_{20}), 155.59(C_{30}), 148.5 (C_{15}), 143.8 (C_{11}), 136.9, 132.0 (C_{12}, C_{15}), 129.2, 128.7, 127.6, 127.0, 126.9, 124.9, 124.2, 120.2(other aromatic and vinylic carbons), 116.3, 116.0(C_{14}, C_{16}), 114.4, 114.3(C_{31}, C_{36}), 110.2(C_{33}), 49.3 (C_{25}, C_{29}), 49.1(C_{26}, C_{28}).

Anal. Calcd for $C_{32}H_{29}N_3O_2$ (487.60): C, 78.82; H, 6.00; N, 8.62; O, 6.56. Found: C, 78.72; H, 5.97; N, 6.68.

1-(4-((E)-4-((E)-4-nitrostyryl)styryl)phenyl)-*1H*-imidazole (8d).

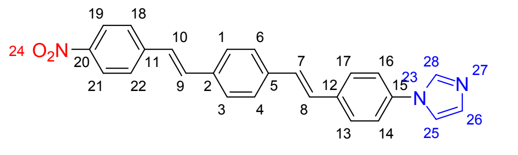

8d

Into a conical flask (25 mL) a mixture of 1-(4-bromophenyl)-*1H*-imidazole (5.0 mmol, 0.22 g), (E)-1-nitro-4-(4-vinylstyryl)benzene(1.0 mmol, 0.25 g), K_2CO_3 (2.0 mmol, 0.28 g), NHC-Pd catalyst (12.0mg, 1.5mol %) and DMF (5 mL) heated in an oil bath at 120 °C for 12 h. The reaction was followed by TLC. The workup process was same to compound **8a**. The crude product was purified by column chromatography, eluting with *n*-hexane/EtOAc/MeOH 20:2:1→20:4:1 (v/v), to afford the title compound.

Yield: 78%.

Yellow solid (M.p.87-93 °C).

IR (KBr) ν: 3031, 2923, 2846, 1689, 1596, 1512, 1342, 1172, 1110, 964, 817, 694 cm^{-1}.

^{1}H NMR (250 MHz, $CDCl_3$): δ (ppm) = 8.22 (d, J = 8.75 Hz, 2H, C_{19}, C_{21}-H) 7.90(s, 1H, C_{28}-H), 7.66- 7.61 (m, 5H, aromatic and vinylic-H), 7.55- 7.37 (m, 6H, aromatic and vinylic-H), 7.30- 7.16 (m, 5H, aromatic and vinylic-H).

^{13}C-NMR (100 MHz, CDCl$_3$): δ (ppm) = 146.8 (C_{20}), 143.7 (C_{11}), 139.7 (C_{16}), 137.0(C_2), 136.8(C_6), 132.8, 132.2, 132.1, 131.2, 129.4, 128.9, 128.8, 128.4, 127.0, 125.6, 124.2, 122.6, 121.6 (other aromatic and vinylic carbons), 120.5(C_{25}).

Anal. Calcd for $C_{25}H_{19}N_3O_2$ (393.45): C, 76.32; H, 4.87; N, 10.68; O, 8.13. Found: C, 76.22; H, 4.90; N, 10.49.

(4-((E)-4-((E)-4-nitrostyryl)styryl)phenyl)alanine(8e).

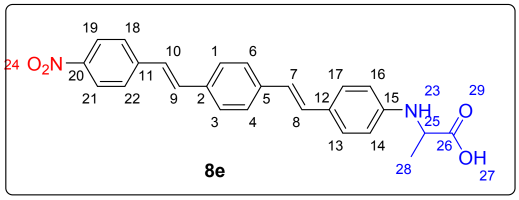

8e

Into a conical flask (10 mL) a mixture of (4-bromophenyl)alanine (**7e**) (1.0 mmol, 0.24 g), (E)-1-nitro-4-(4-vinylstyryl)benzene(1.0 mmol, 0.25 g), K_2CO_3 (2.0 mmol, 0.28 g), NHC-Pd catalyst (12.0mg, 1.5mol %) and DMF (5 mL) heated in an oil bath at 110 °C for 12 h. The reaction was followed by TLC. The workup process was same to compound **7e**. The crude product was purified by column chromatography, eluting with *n*-hexane/EtOAc/MeOH 20:2:1→ 20:4:2 (v/v), to afford the title compound.

Yield: 74%.

Yellow solid (M.p.107-109 °C).

IR (KBr) ν: 3371, 3039, 2923, 2854, 1689, 1589, 1512, 1334, 1180, 1110, 964, 833, 694 cm^{-1}.

^{1}H NMR (250 MHz, CDCl$_3$): δ (ppm) = 8.19 (d, *J* = 10.0 Hz, 2H, C_{19}, C_{20}-H), 7.89-7.81 (m, 2H,), 754- 7.51 (d, *J* = 11.60 Hz, 2H, aromatic and vinylic-H), 7.64-6.93 (m, 12 H, aromatic and vinylic-H), 6.64 (d, *J* = 4.3 Hz, 2H, aromatic and vinylic-H), 5.28 (s, 2H, OH,NH), 2.67 (q, *J* = 7.5 Hz, 1H, C_{25}-H), 1.25 (d, *J* = 7.5 Hz, 3H, C_{28}-H).

^{13}C-NMR (100 MHz, CDCl$_3$): δ (ppm) = 176.2 (C=O), 146.7(C_{20}), 143.7 (C_{15}), 136.6(C_{11}), 132.2 (C_2), 132.1(C_5), 131.2 (C_{13}), 129.9(C_{17}), 128.9, 128.7, 127.9, 126.9, 124.2, 123.5(other aromatic and vinylic carbons), 116.8(C_{12}), 113.9(C_{14}, C_{16}), 52.1(C_{25}), 18.9(C_{28}).

Anal. Calcd for $C_{25}H_{22}N_2O_4$ (414.46): C, 72.45; H, 5.35; N, 6.76; O, 15.44. Found: C, 72.51; H, 5.26; N, 6.70.

3. Fluorescence data

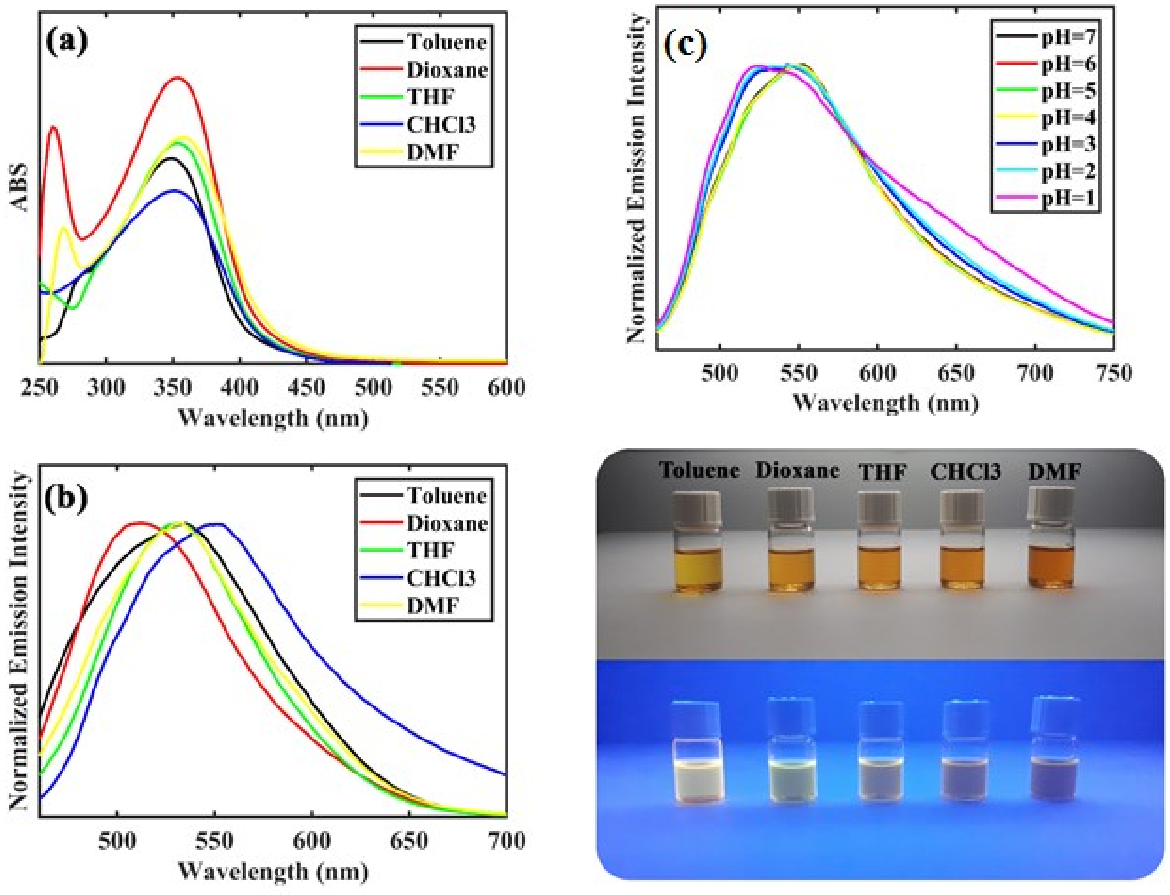

Figure 1S. Uv–Vis spectra (a),emission spectra (b) and pH sensitivity(c) of 8a at concentration of 10^{-5} M in different solvents. The photographs of the compound in different solutions [From leftto right, Toluene, Dioxane, Tetrahydrofuran (THF), Chloroform (CHCl3), and dimethyl formamide (DMF)] were taken under natural daylight simulator (D65) lamps (top image), and irradiation of A-Class UV lamps (bottom image).

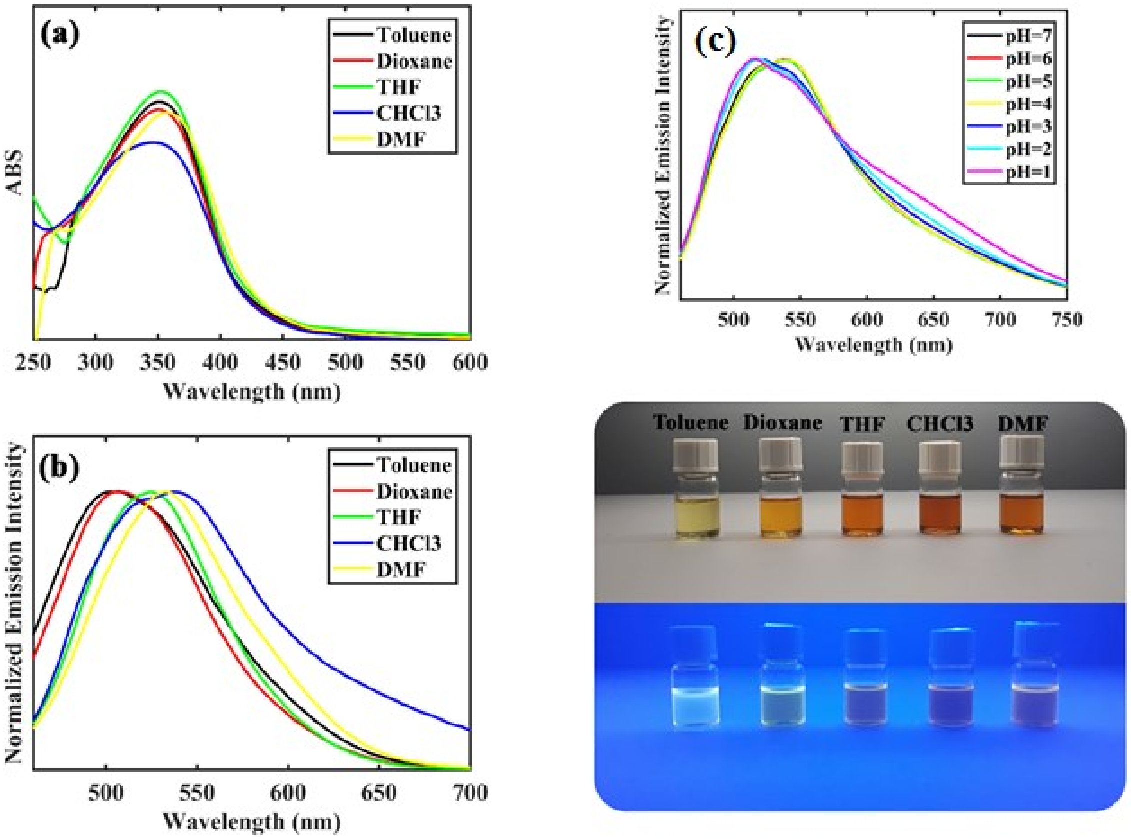

Figure 2S. Uv–Vis spectra (a),emission spectra (b) and pH sensitivity(c) of **8b** at concentration of 10^{-5} M in different solvents. The photographs of the compound in different solutions [From left to right, Toluene, Dioxane, Tetrahydrofuran (THF), Chloroform (CHCl3), and dimethyl formamide (DMF)] were taken under natural daylight simulator (D65) lamps (top image), and irradiation of A-Class UV lamps (bottom image).

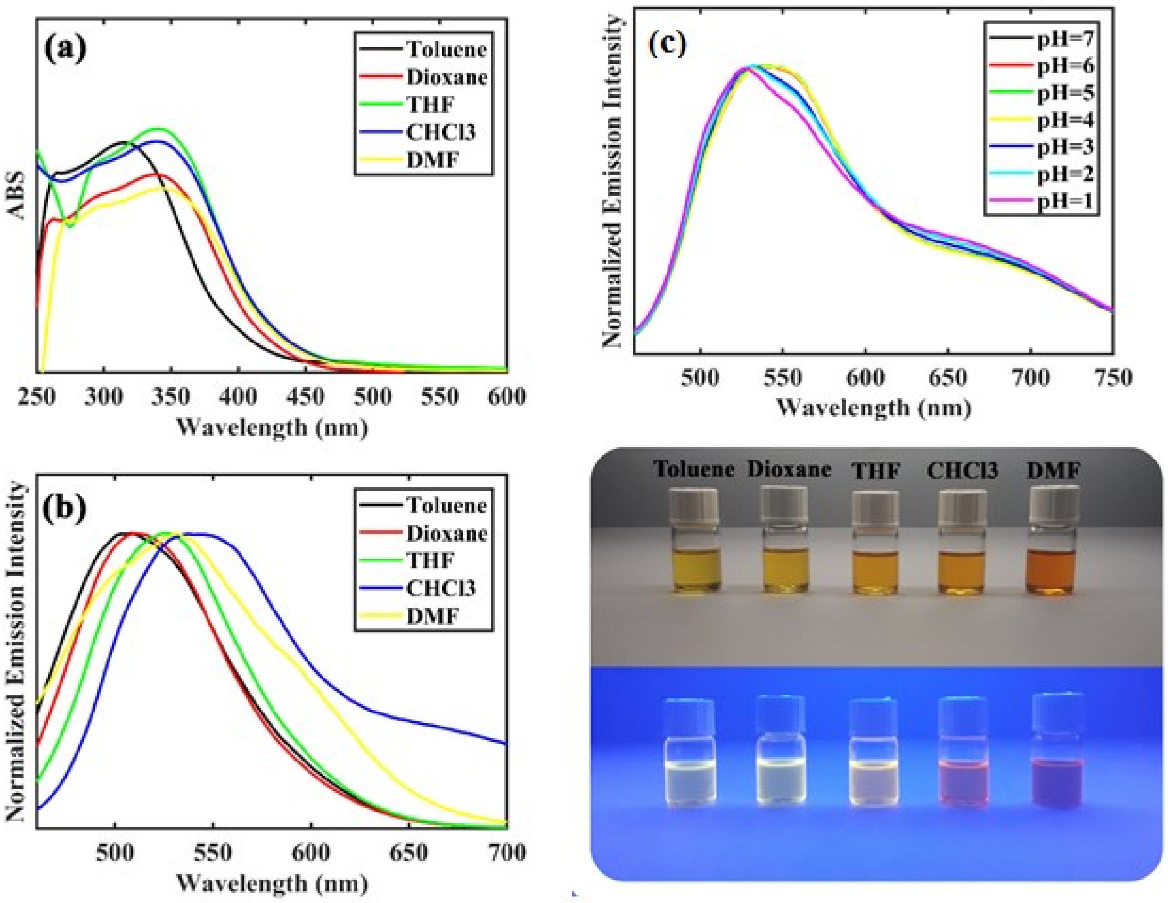

Figure 3S. Uv–Vis spectra (a),emission spectra (b) and pH sensitivity(c) of 8c at concentration of 10^{-5} M in different solvents. The photographs of the compound in different solutions [From left to right, Toluene, Dioxane, Tetrahydrofuran (THF), Chloroform (CHCl3), and dimethyl formamide (DMF)] were taken under natural daylight simulator (D65) lamps (top image), and irradiation of A-Class UV lamps (bottom image).

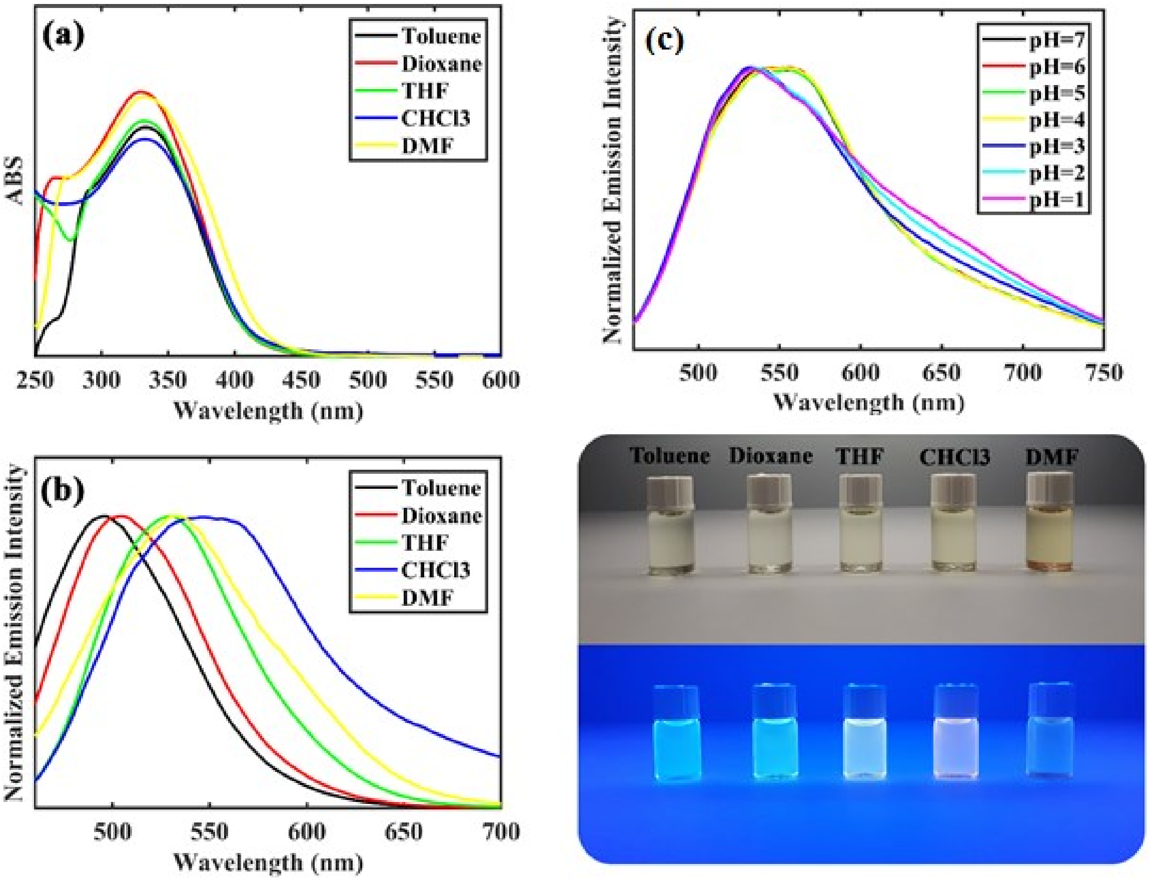

Figure 4S. Uv–Vis spectra (a),emission spectra (b) and pH sensitivity(c) of 8d at concentration of 10^{-5} M in different solvents. The photographs of the compound in different solutions [From left to right, Toluene, Dioxane, Tetrahydrofuran (THF), Chloroform (CHCl3), and dimethyl formamide (DMF)] were taken under natural daylight simulator (D65) lamps (top image), and irradiation of A-Class UV lamps (bottom image).

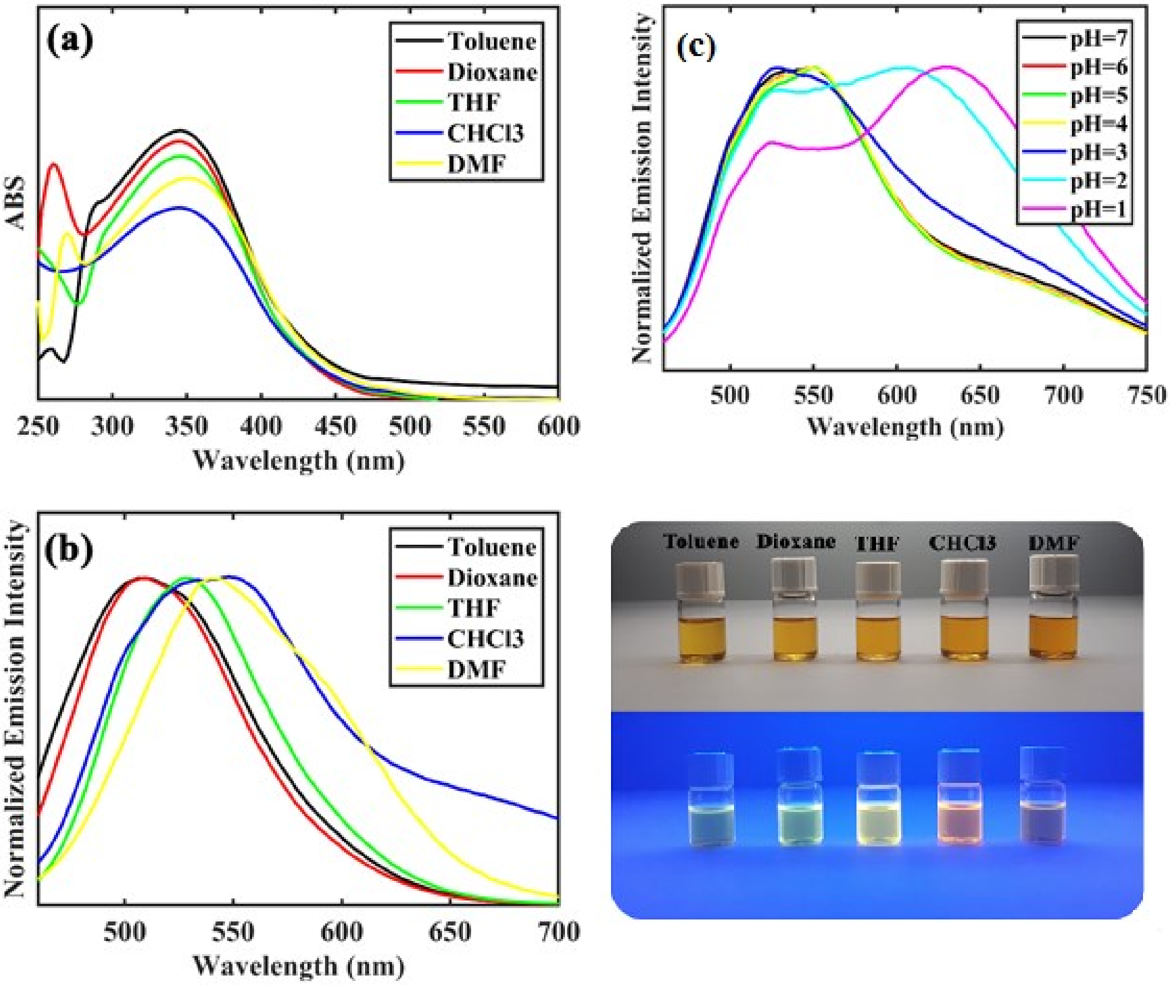

Figure 5S. Uv–Vis spectra (a), emission spectra (b) and pH sensitivity(c) of **8e** at concentration of 10^{-5} M in different solvents. The photographs of the compound in different solutions [From left to right, Toluene, Dioxane, Tetrahydrofuran (THF), Chloroform (CHCl3), and dimethyl formamide (DMF)] were taken under natural daylight simulator (D65) lamps (top image), and irradiation of A-Class UV lamps (bottom image).

2. Copy of ^{1}HNMR and ^{13}CNMR of synthesized compounds

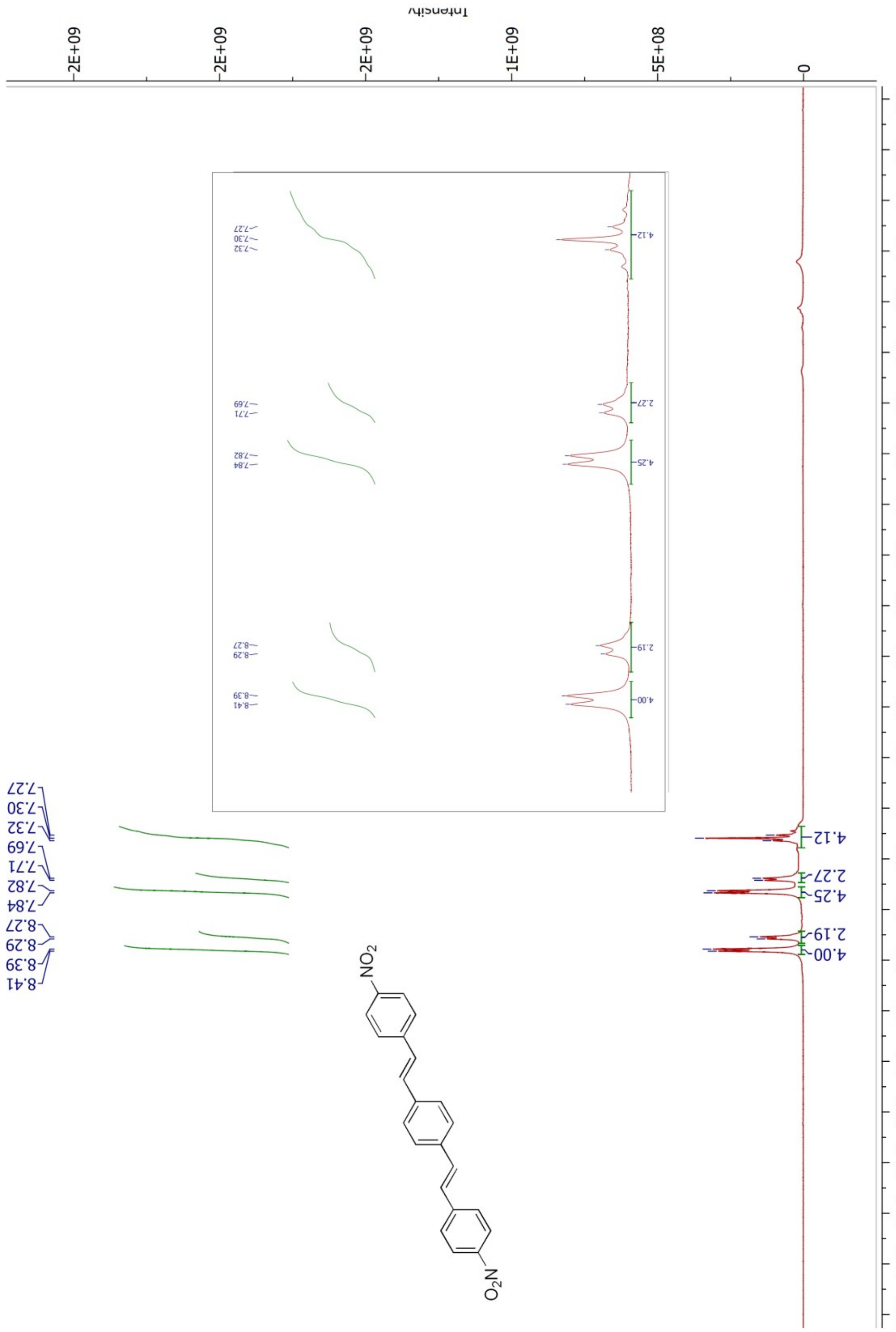

Figure 6S. ^{1}H NMR spectrum of **3a** (250 MHz in $CDCl_3$)

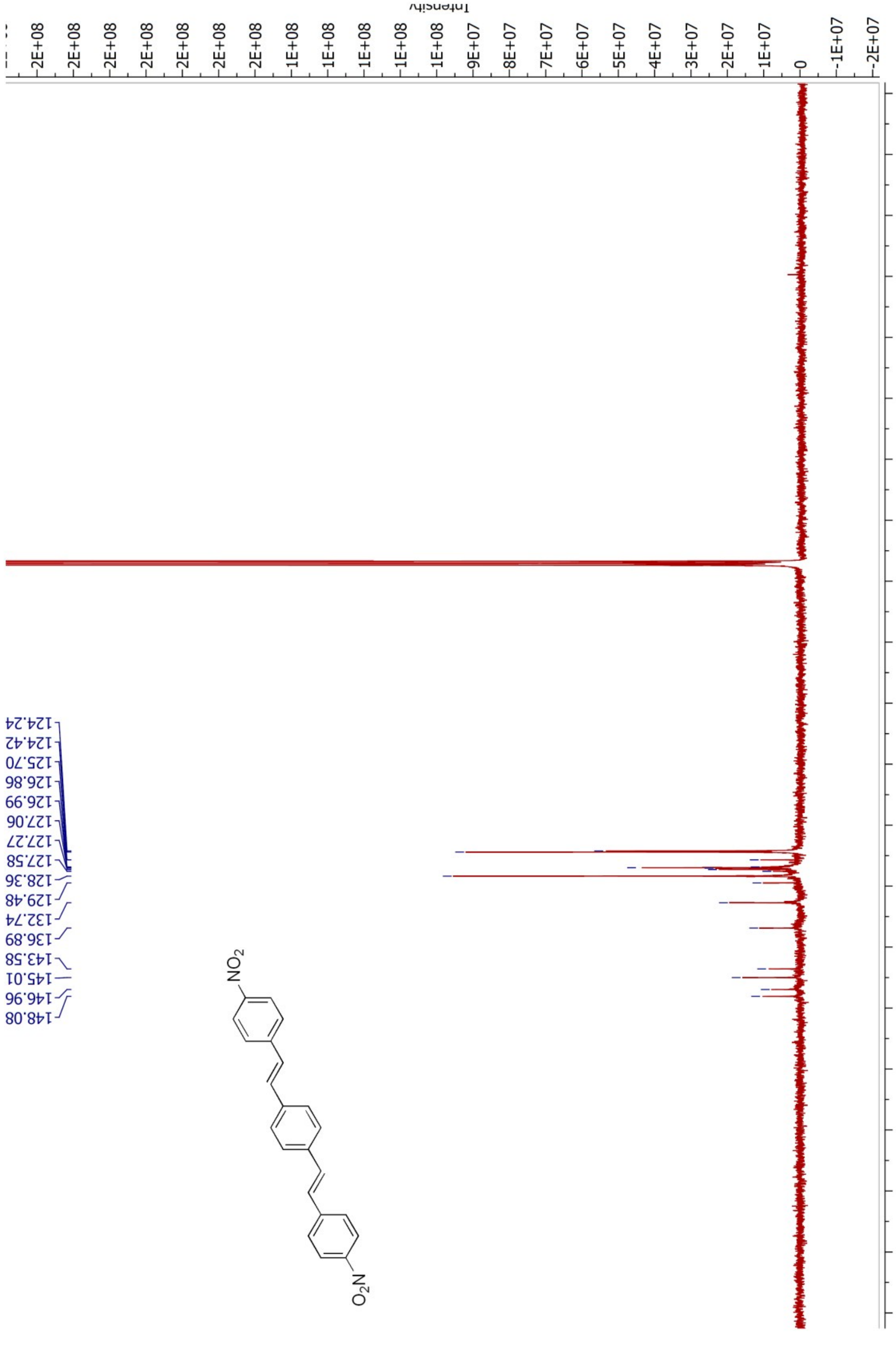

Figure 7S. ^{13}C NMR spectrum of **3a** (100 MHz in $CDCl_3$)

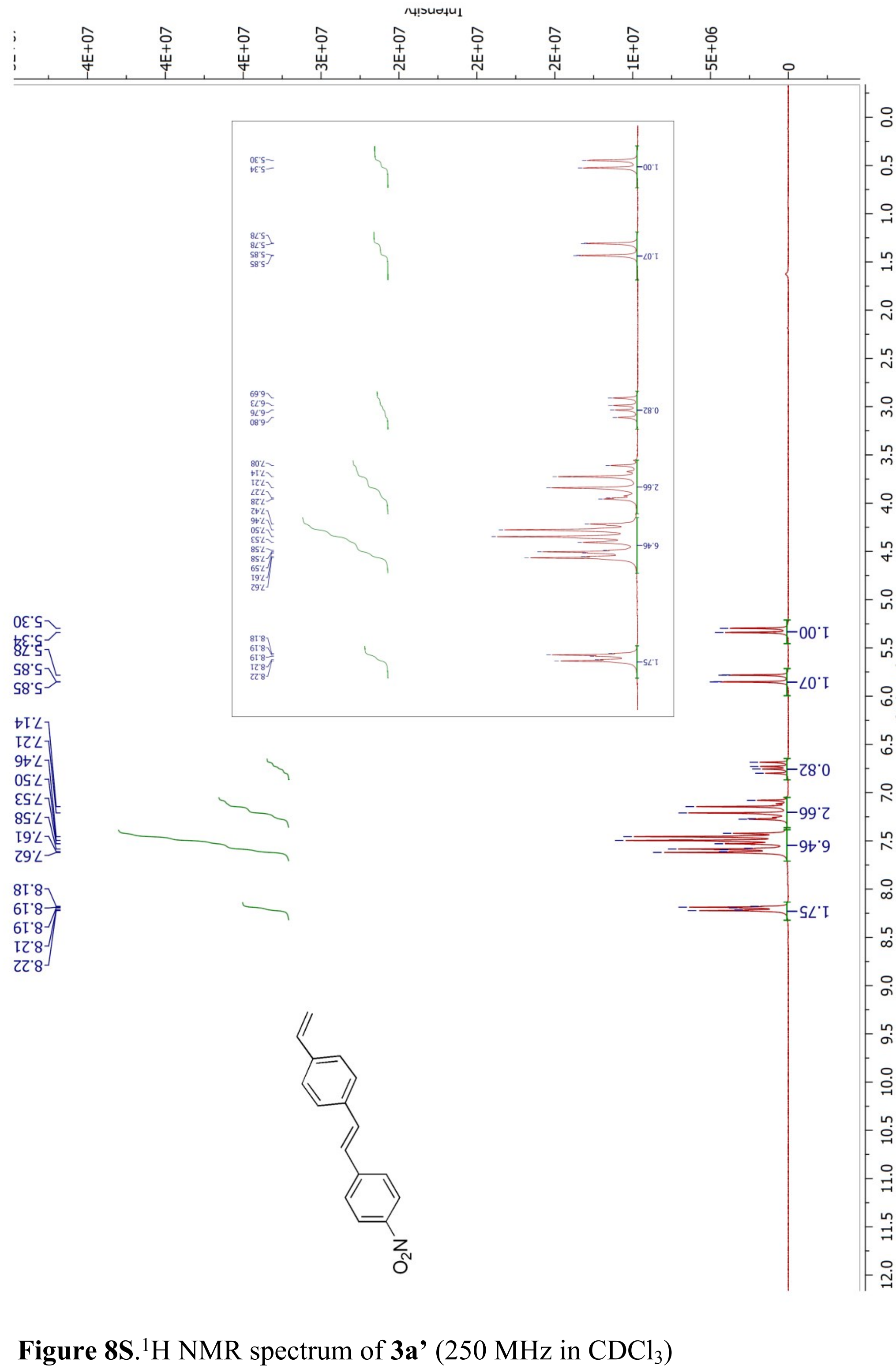

Figure 8S.^{1}H NMR spectrum of **3a’** (250 MHz in $CDCl_3$)

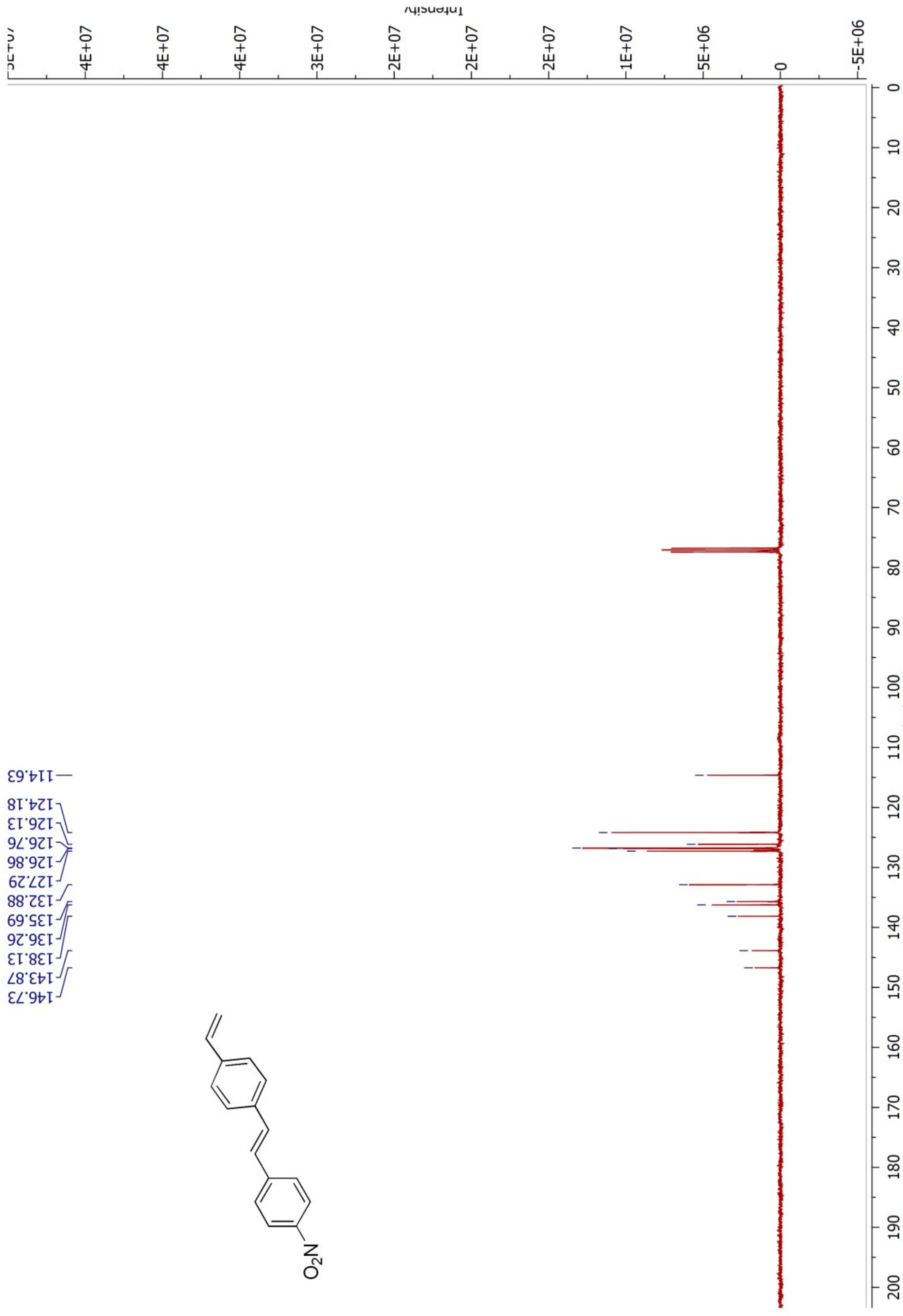

Figure 9S. ^{13}C NMR spectrum of **3a‘** (100 MHz in $CDCl_3$)

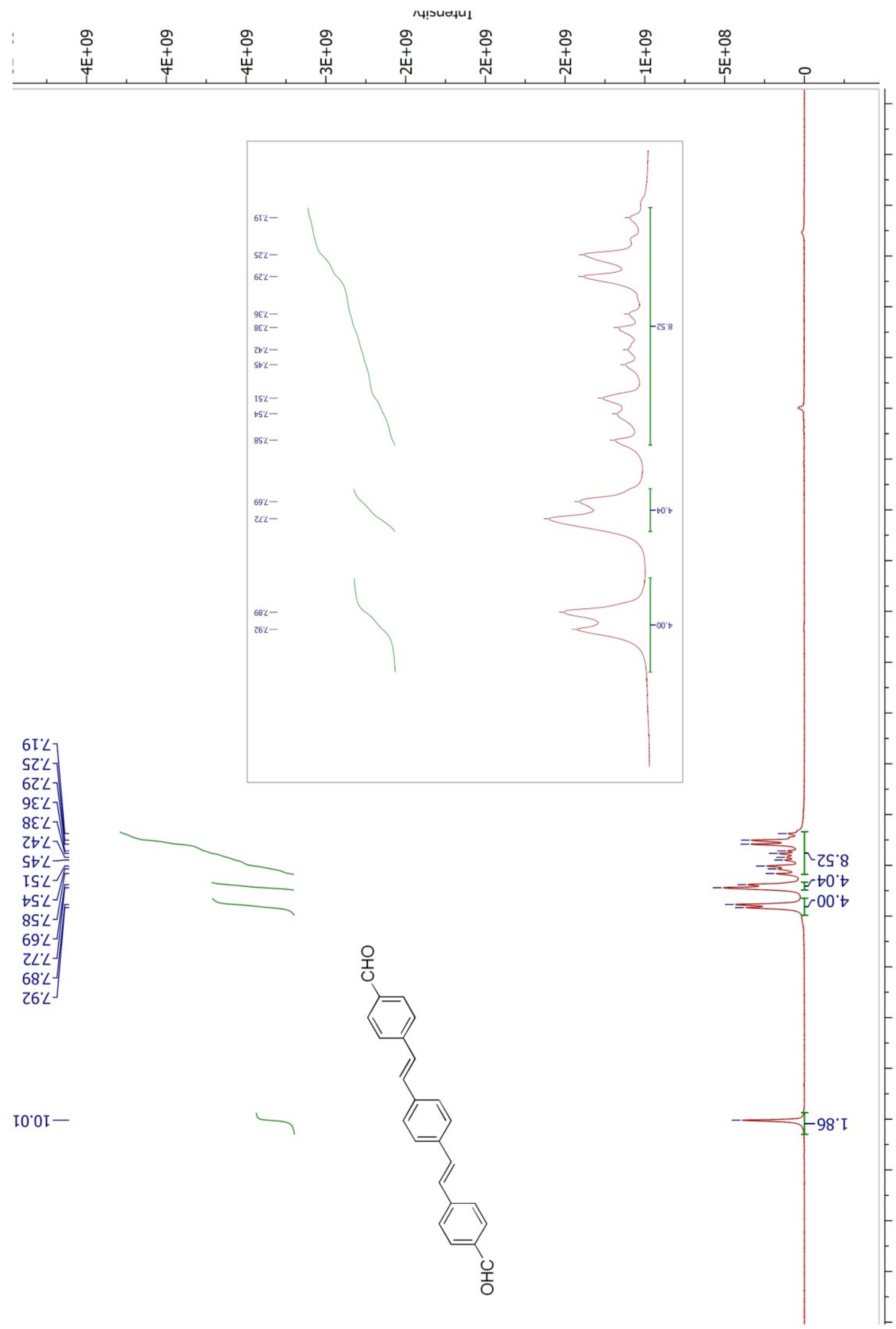
Intensity
f1 (ppm)
10.01
7.92
7.89
7.72
7.69
7.58
7.54
7.51
7.45
7.42
7.38
7.36
7.29
7.25
7.19
8.52
4.04
4.00
1.86
CHO
OHC

Figure 10S. ^{1}H NMR spectrum of **3b** (250 MHz in $CDCl_3$)

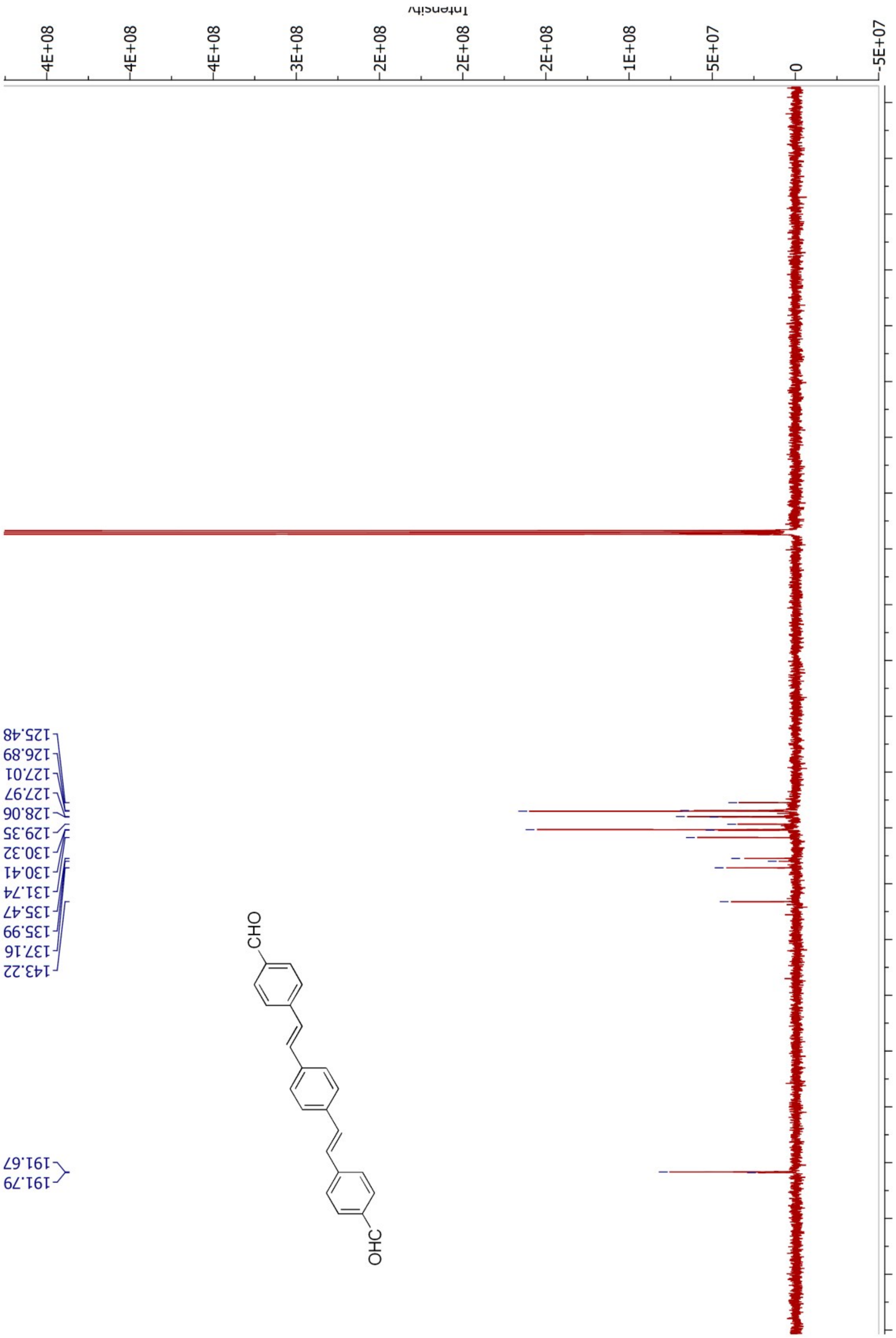

Figure 11S. ^{13}C NMR spectrum of **3b** (100 MHz in $CDCl_3$)

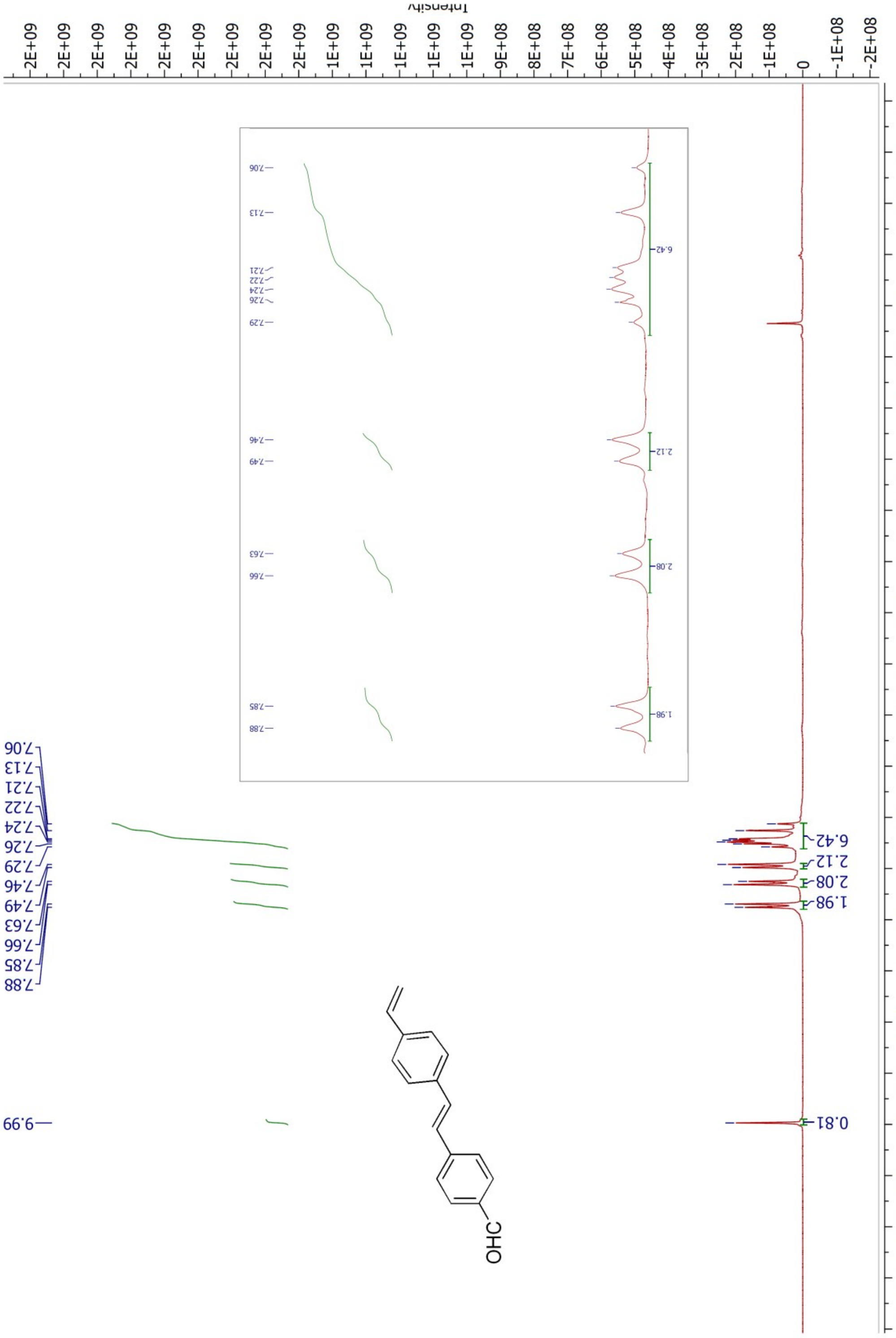

Figure 12S. ^{13}C NMR spectrum of **3b'** (250 MHz in $CDCl_3$)

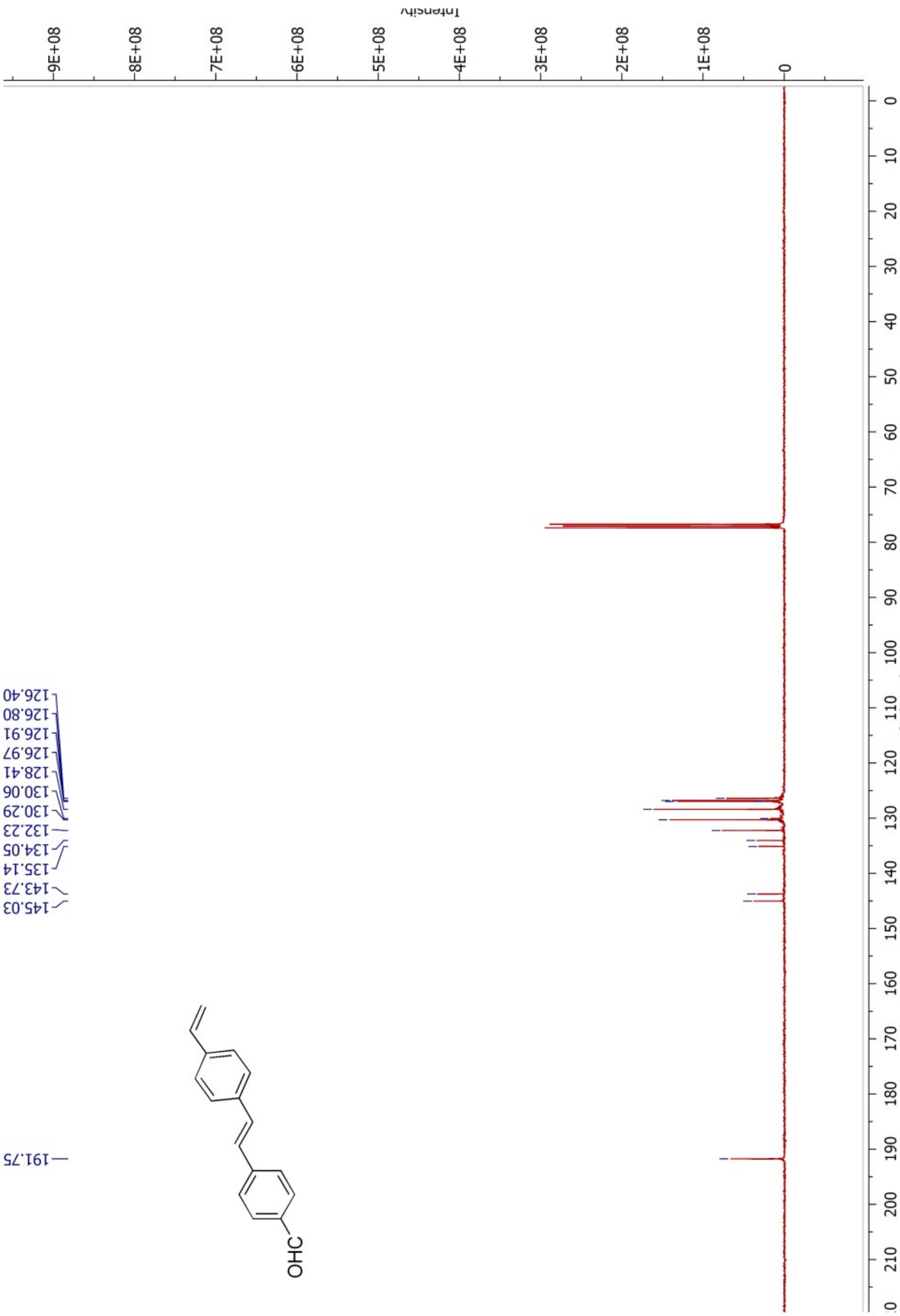

Figure 13S. ^{13}C NMR spectrum of **3b'** (100 MHz in $CDCl_3$)

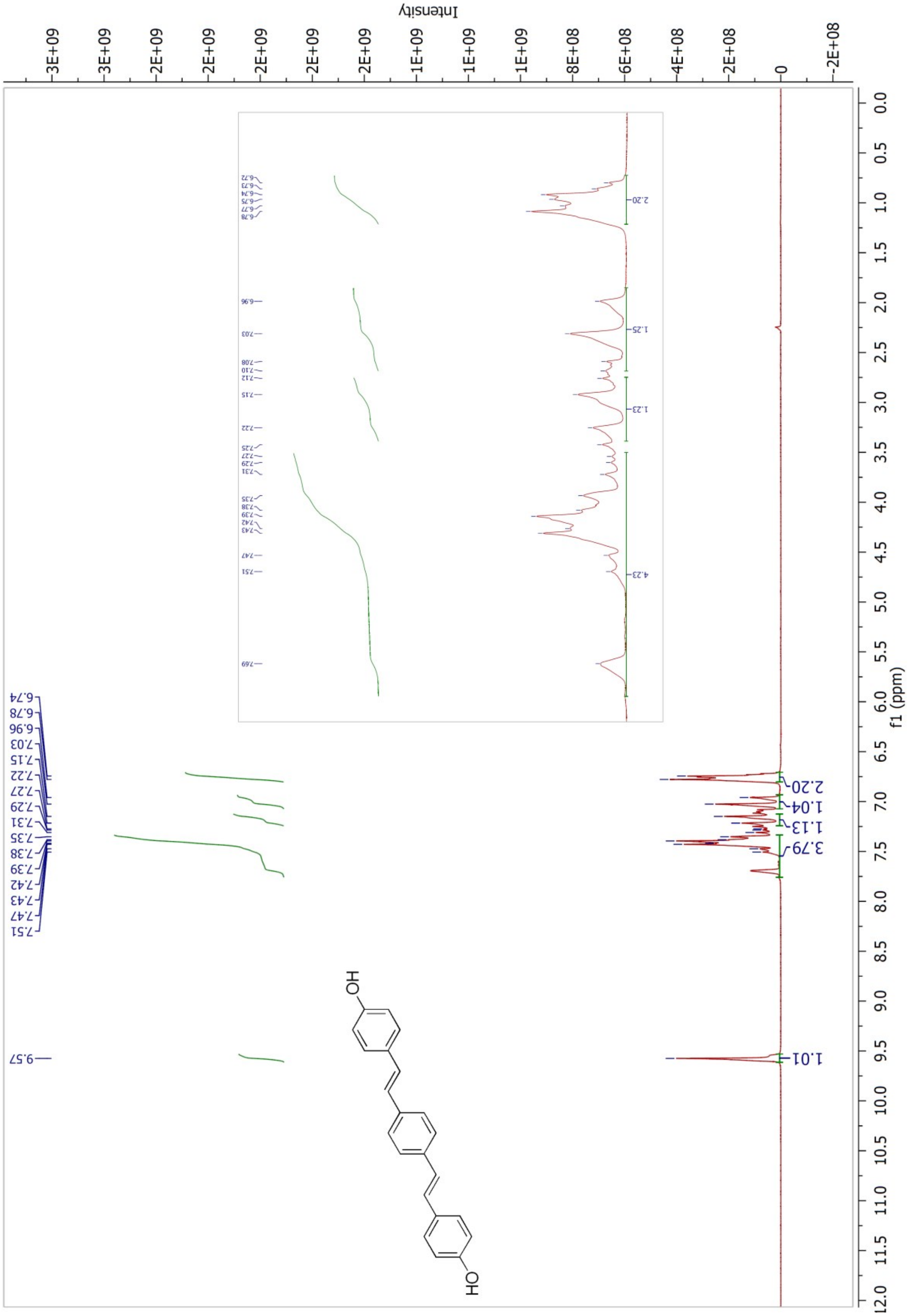

Figure 14S. ^{1}H NMR spectrum of **3d** (250 MHz in $CDCl_3$)

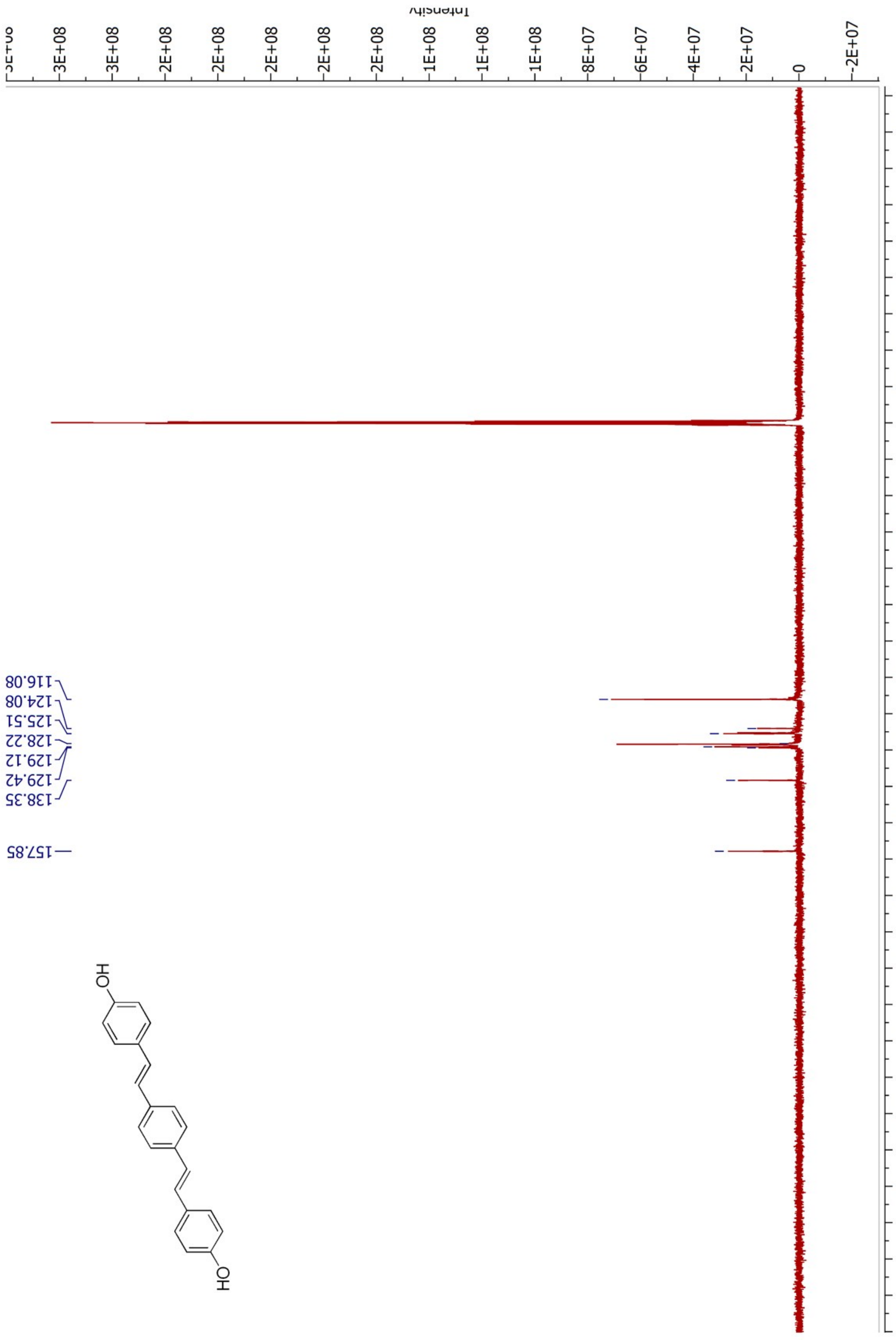

Figure 15S.^{13}C NMR spectrum of **3d** (100 MHz in Acetone-d_6)

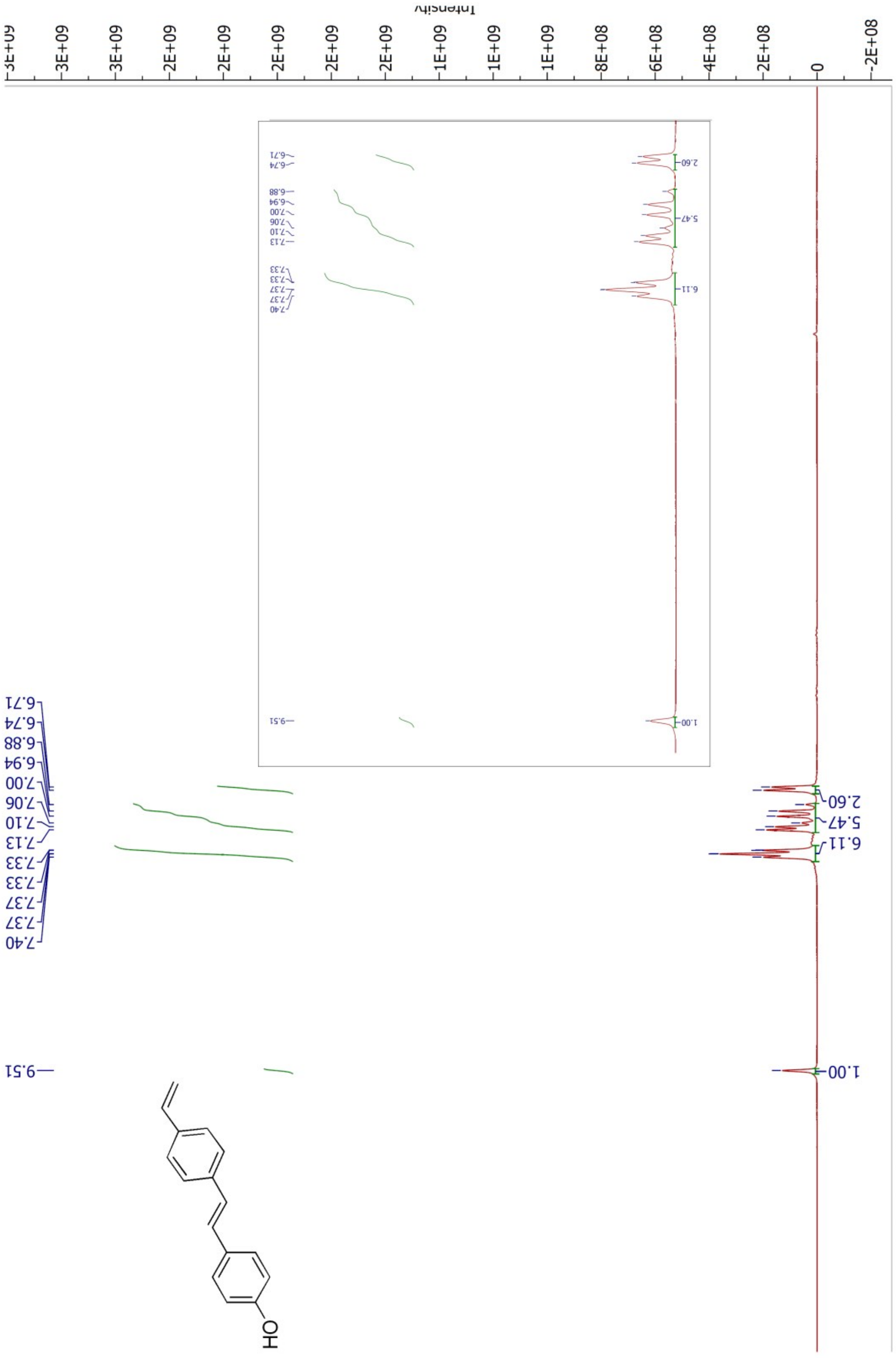

Figure 16S. ^{1}H NMR spectrum of **3d’** (250 MHz in $CDCl_3$)

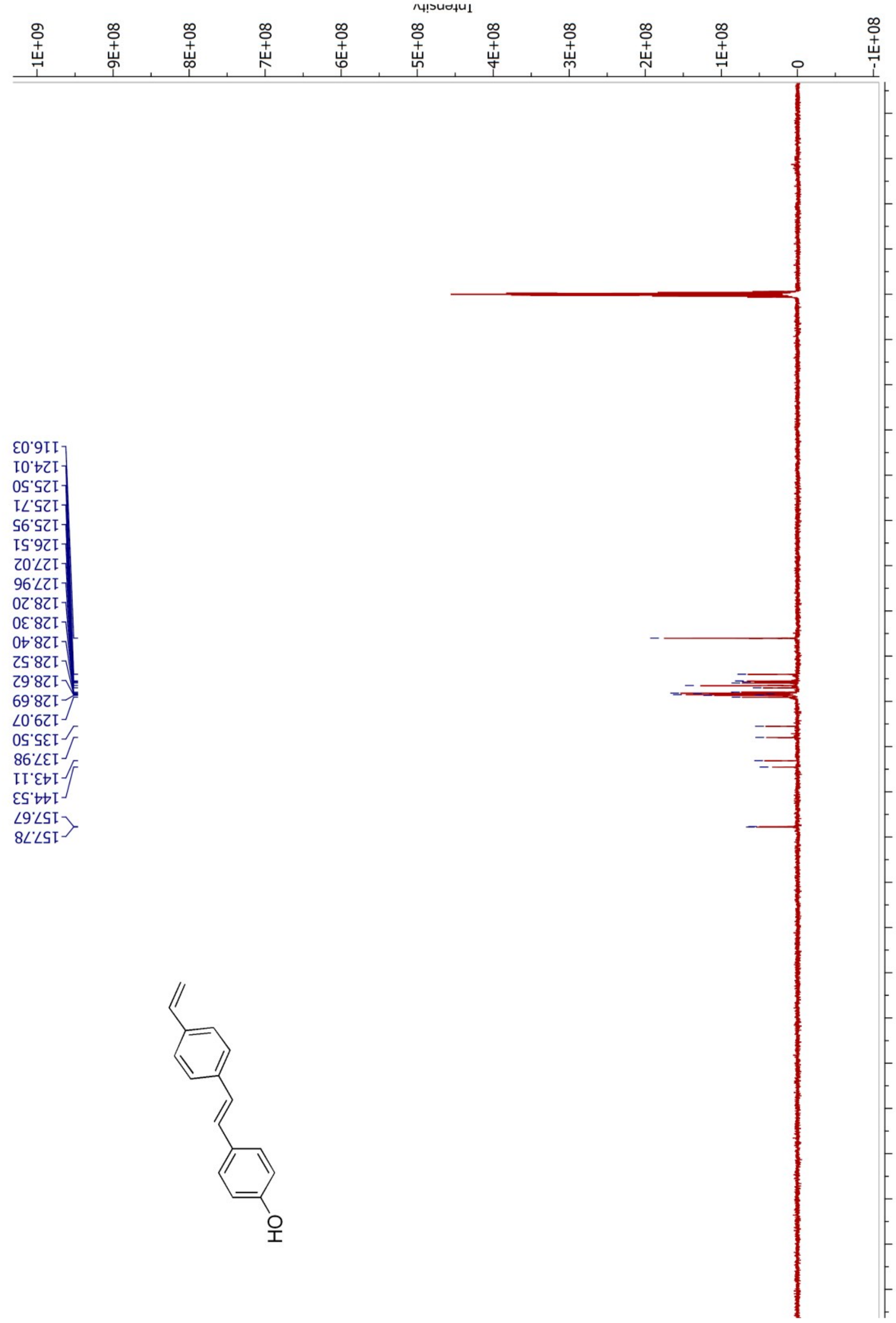

Figure 17S. ^{13}C NMR spectrum of **3d’** (100 MHz in $CDCl_3$)

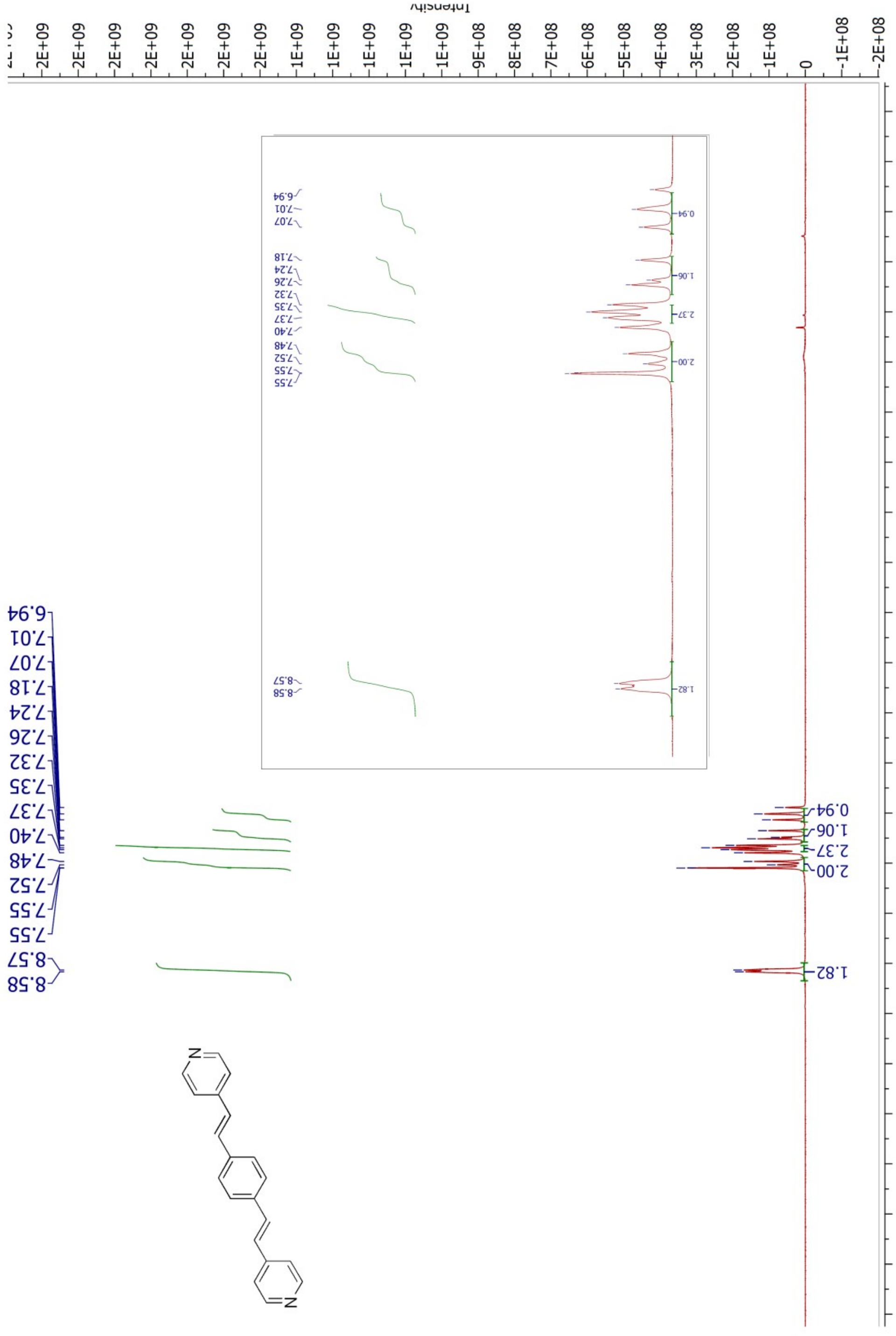

Figure 18S. ^{1}H NMR spectrum of **3e** (250 MHz in CDCl3)

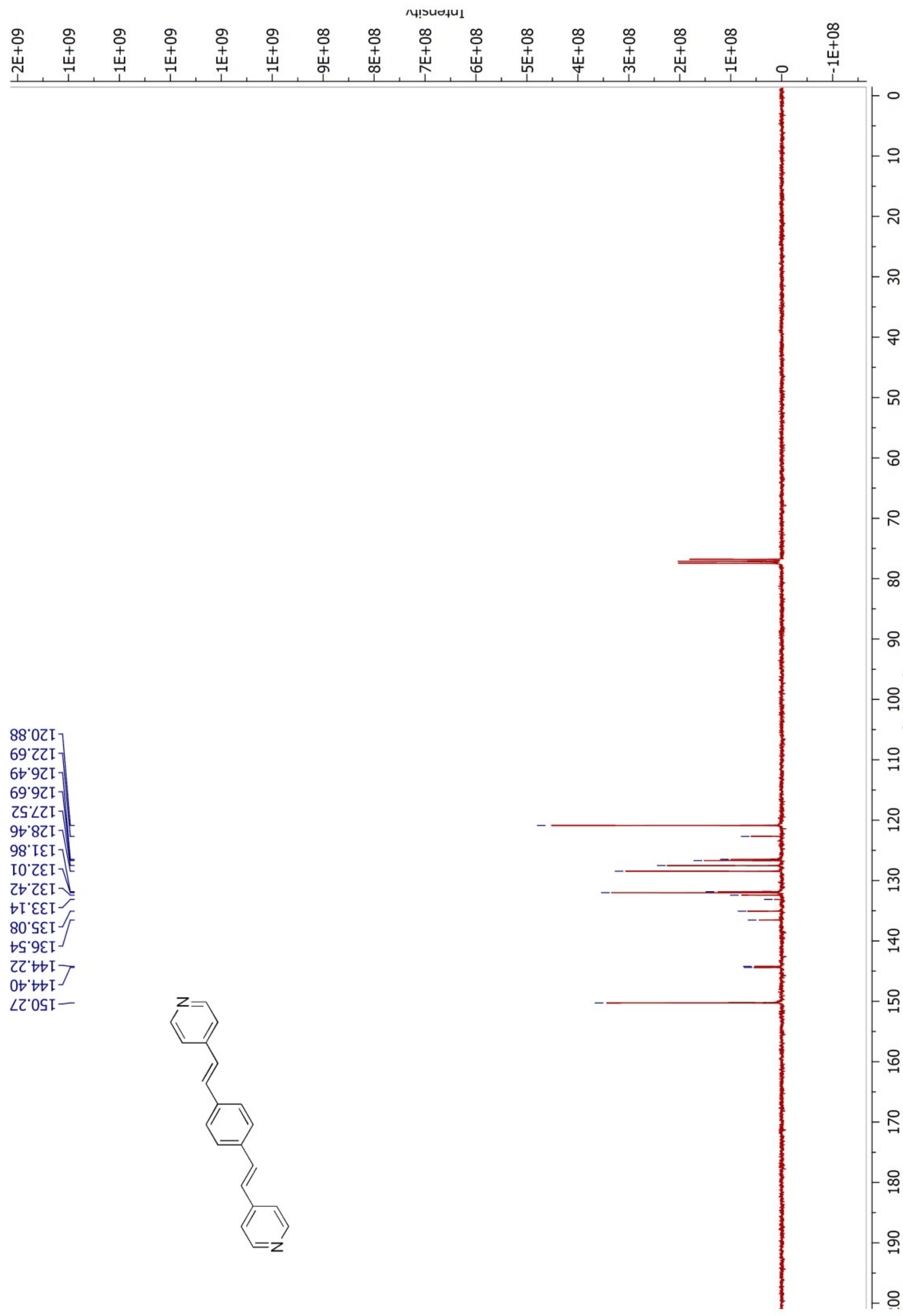

Figure 19S. ^{13}C NMR spectrum of **3e** (62.5 MHz in $CDCl_3$)

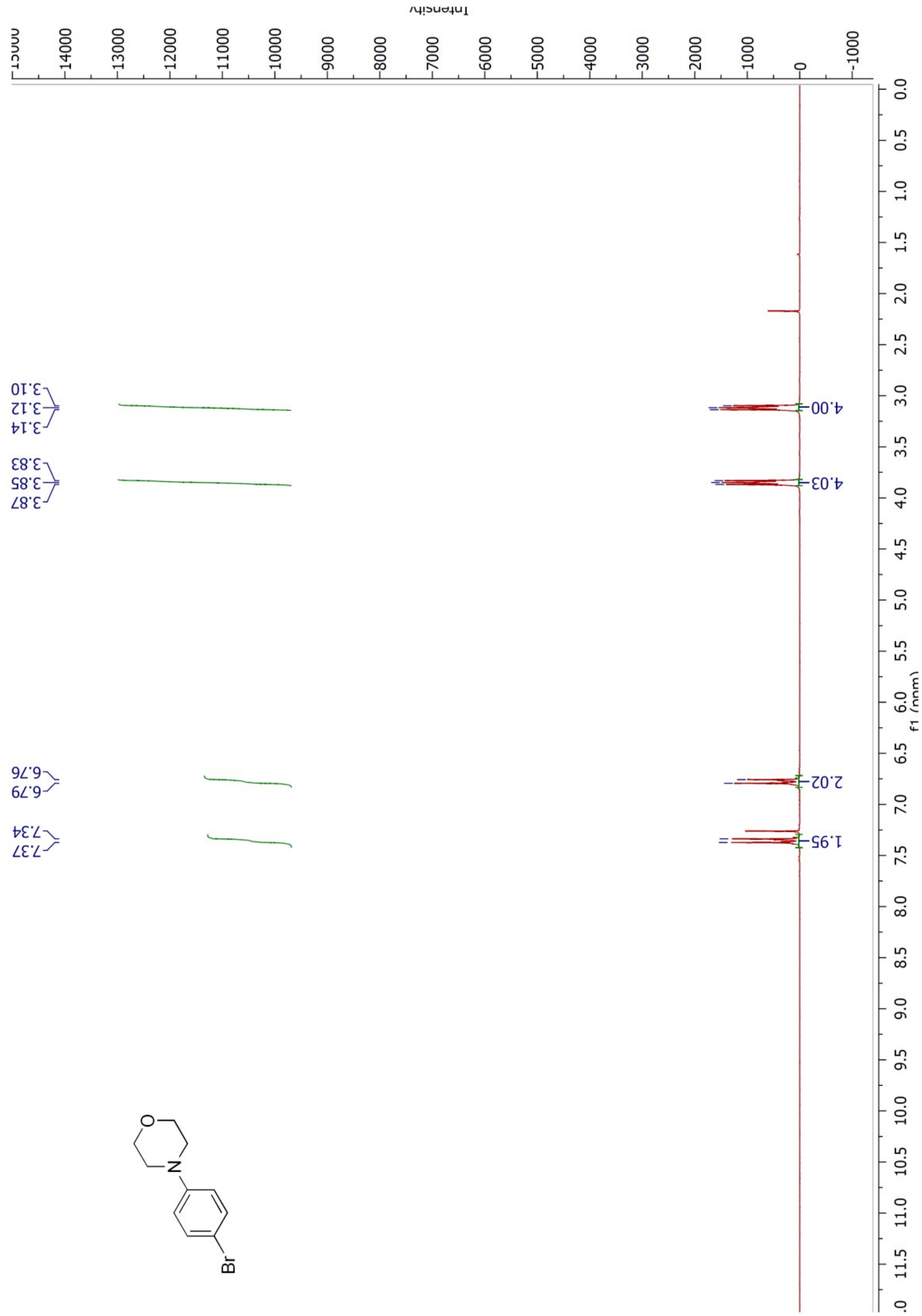

Figure 20S. ^{1}H NMR spectrum of **7a** (250 MHz in $CDCl_3$)

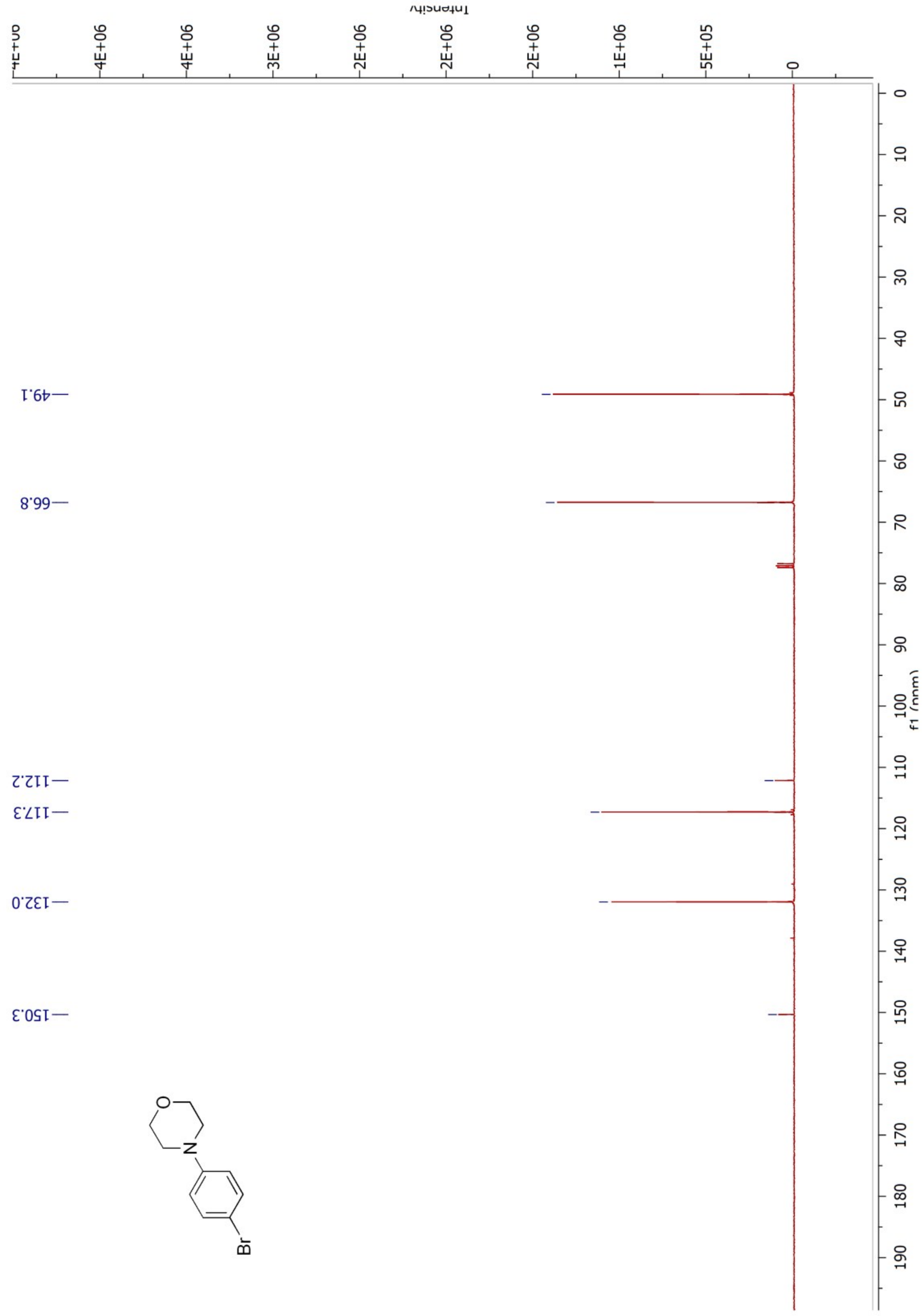

Figure 21S. ^{13}C NMR spectrum of **7a** (62.5 MHz in $CDCl_3$)

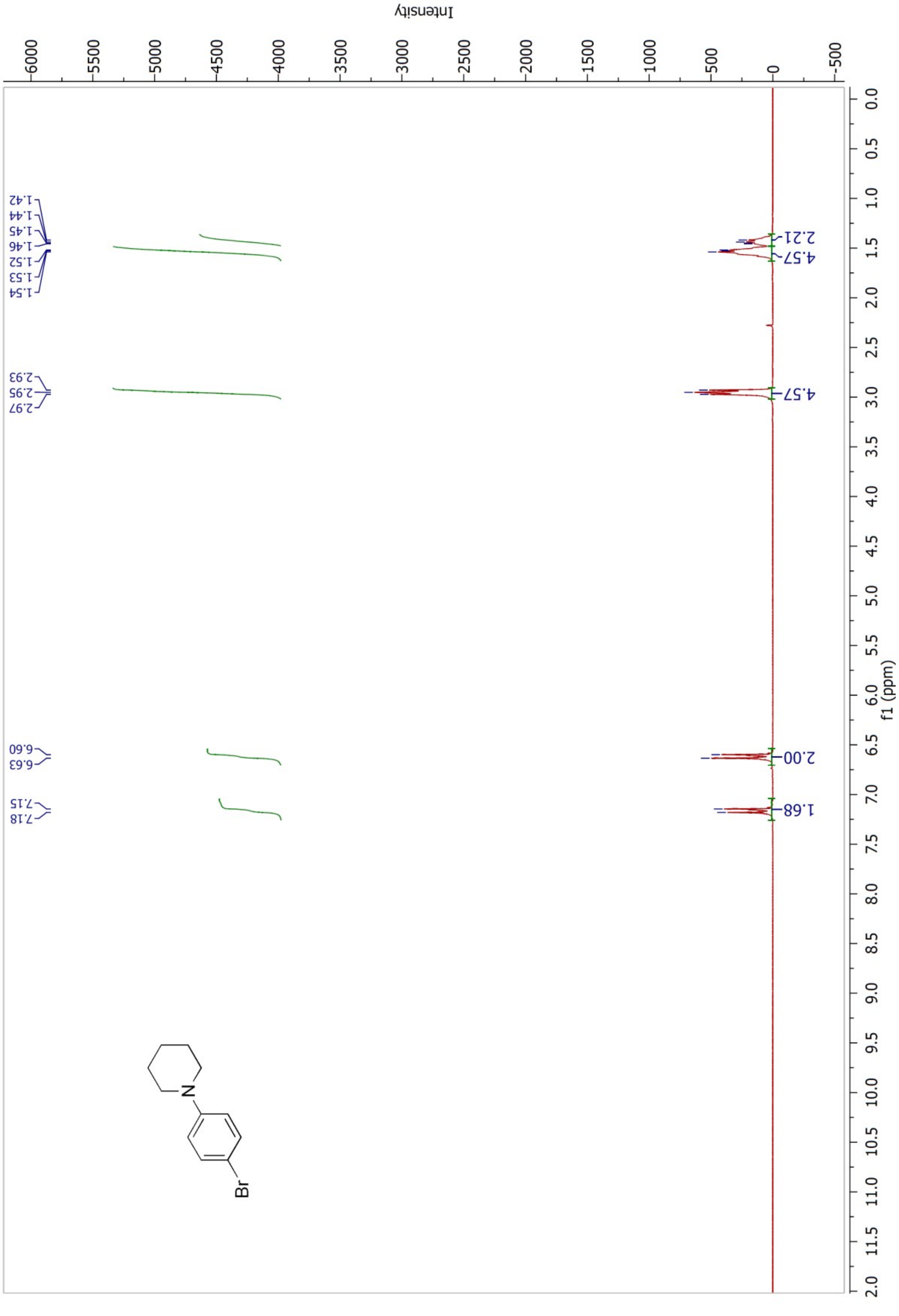

Figure 22S. ^{1}H NMR spectrum of **7b** (250 MHz in $CDCl_3$)

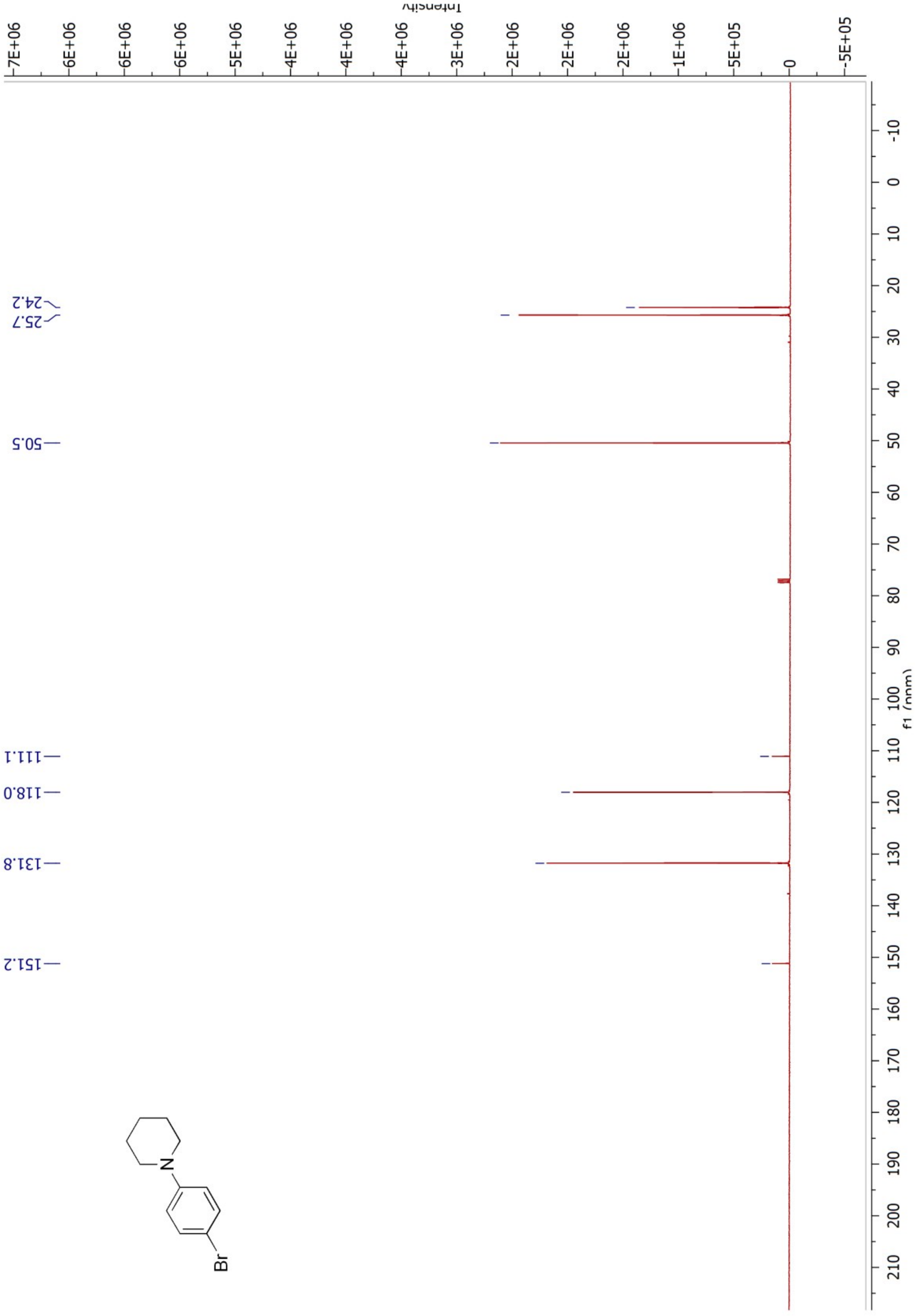

Figure 23S. ^{13}C NMR spectrum of **7b** (62.5 MHz in $CDCl_3$)

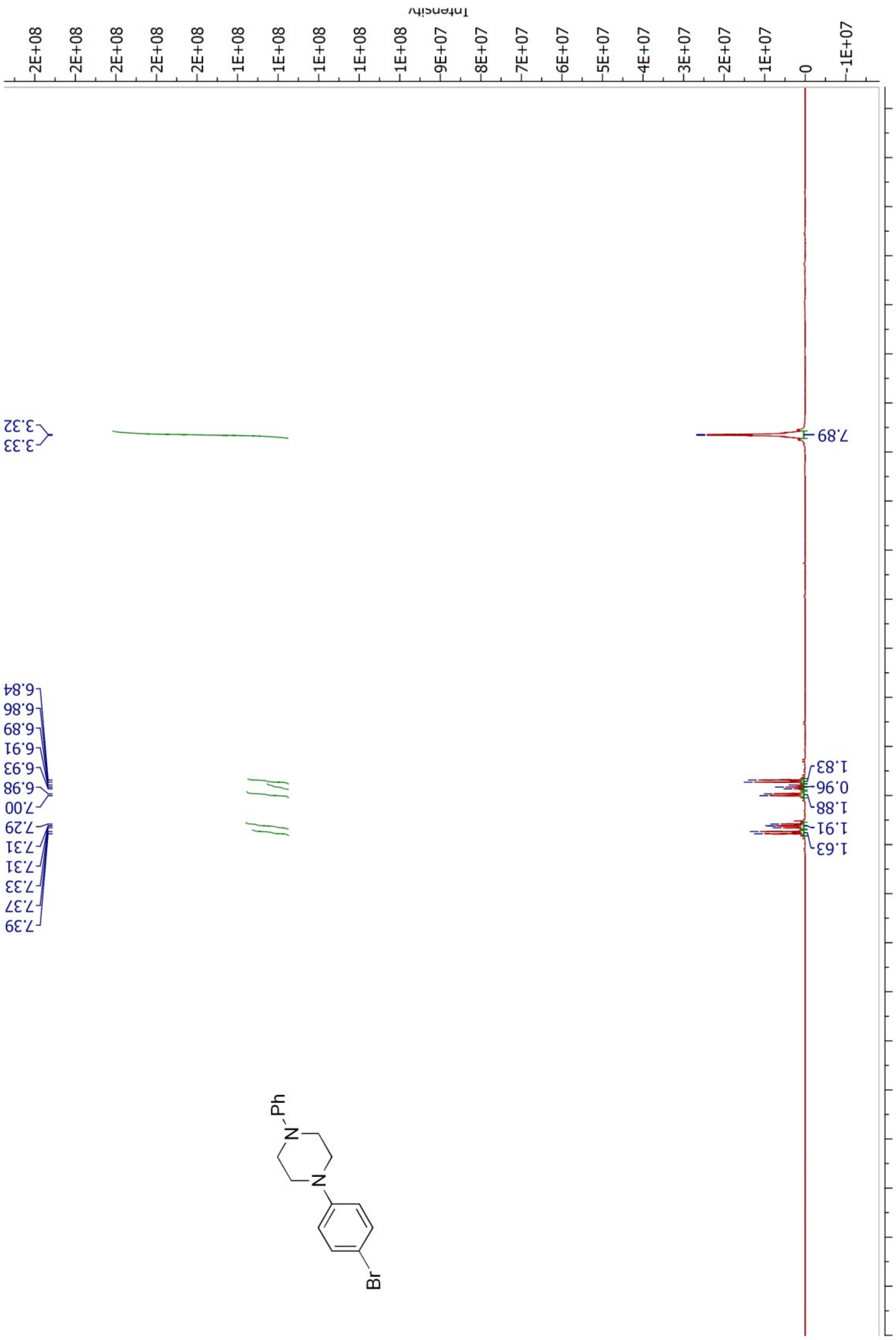

Figure 24S. ^{1}H NMR spectrum of **7c** (400 MHz in $CDCl_3$)

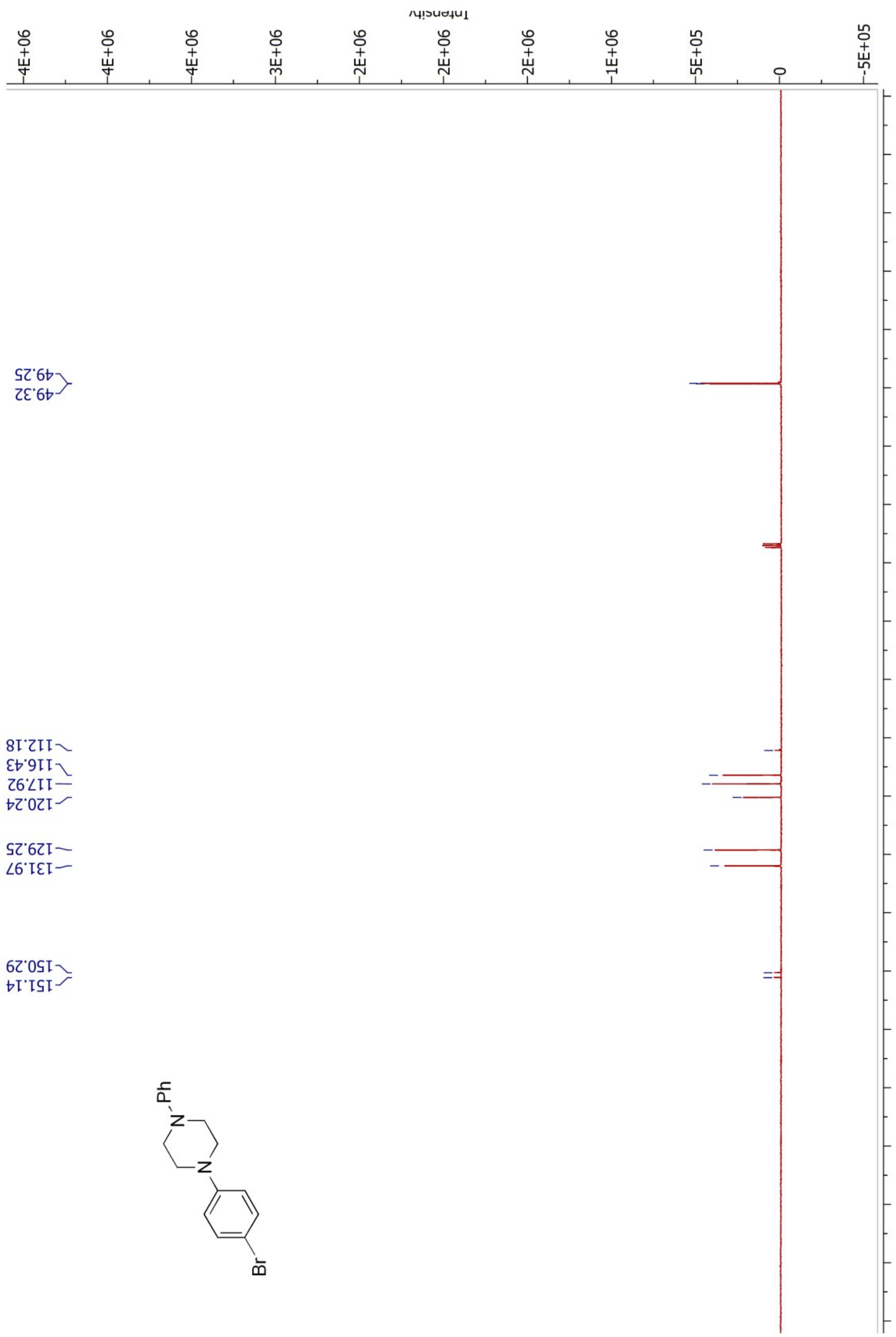

Figure 25S. ^{13}C NMR spectrum of **7c** (100 MHz in $CDCl_3$)

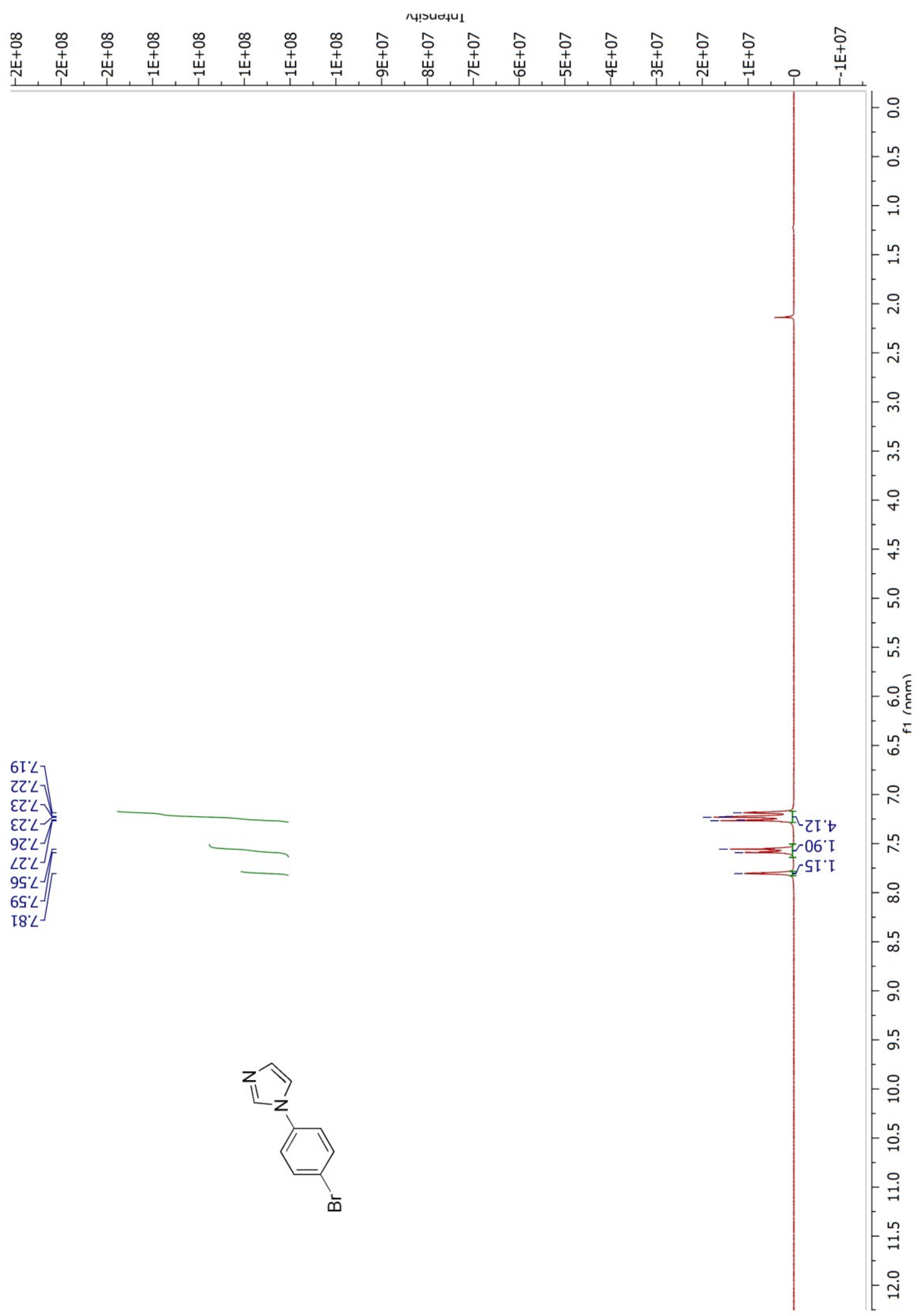

Figure 26S. ^{1}H NMR spectrum of **7d** (250 MHz in $CDCl_3$)

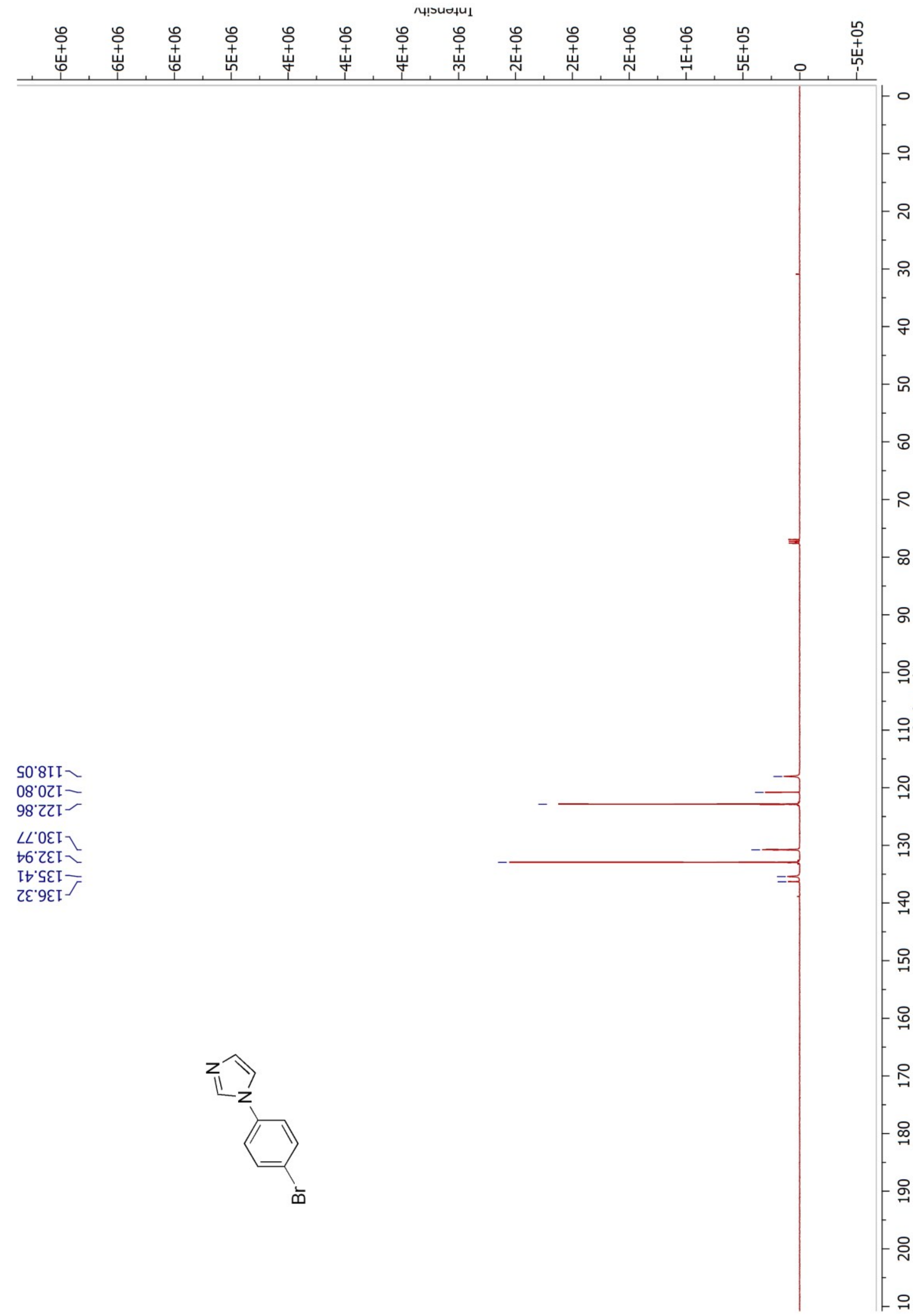

Figure 27S. ^{13}C NMR spectrum of **7d** (100 MHz in $CDCl_3$)

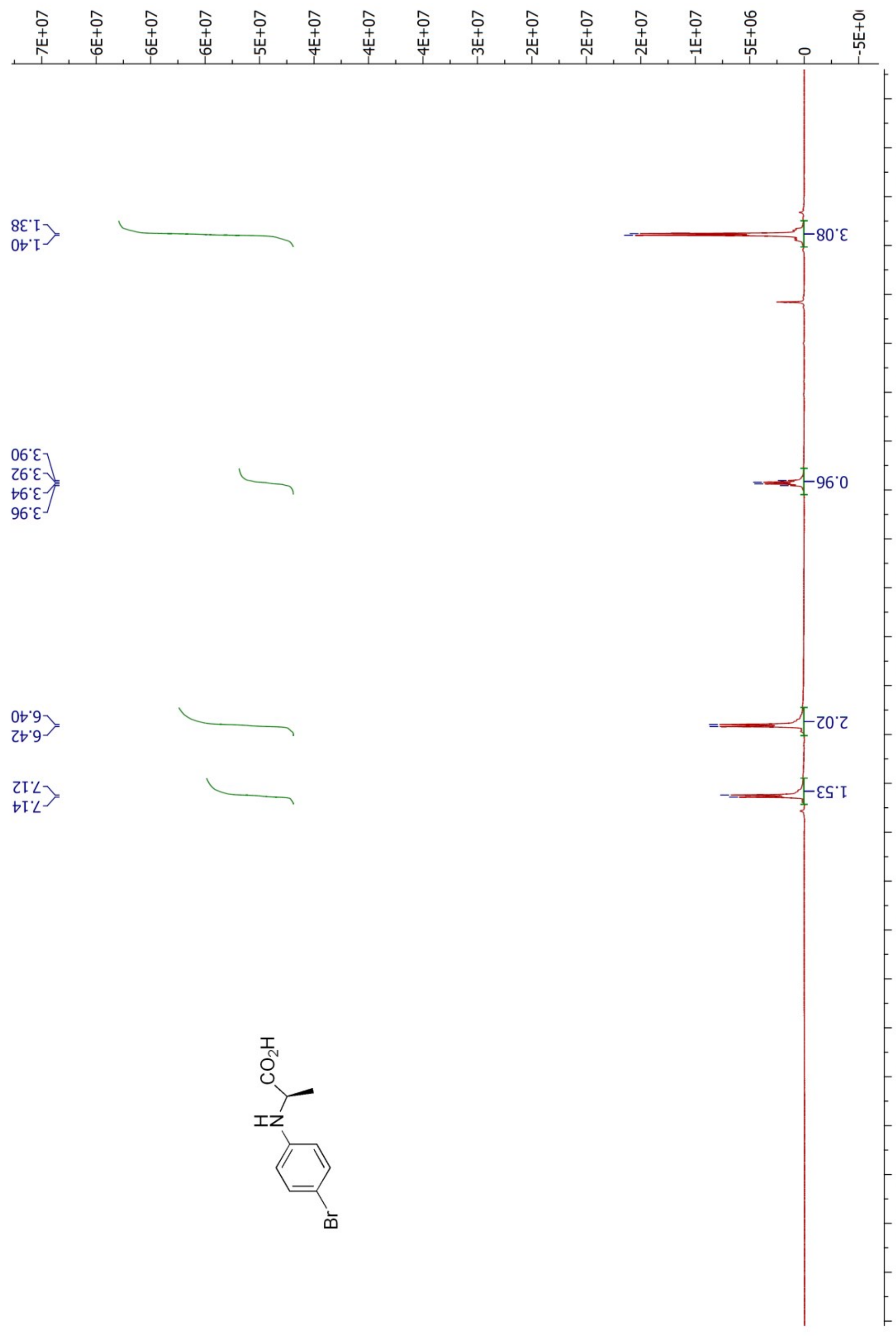

Figure 28S. ^{1}H NMR spectrum of **7e** (250 MHz in $CDCl_3$)

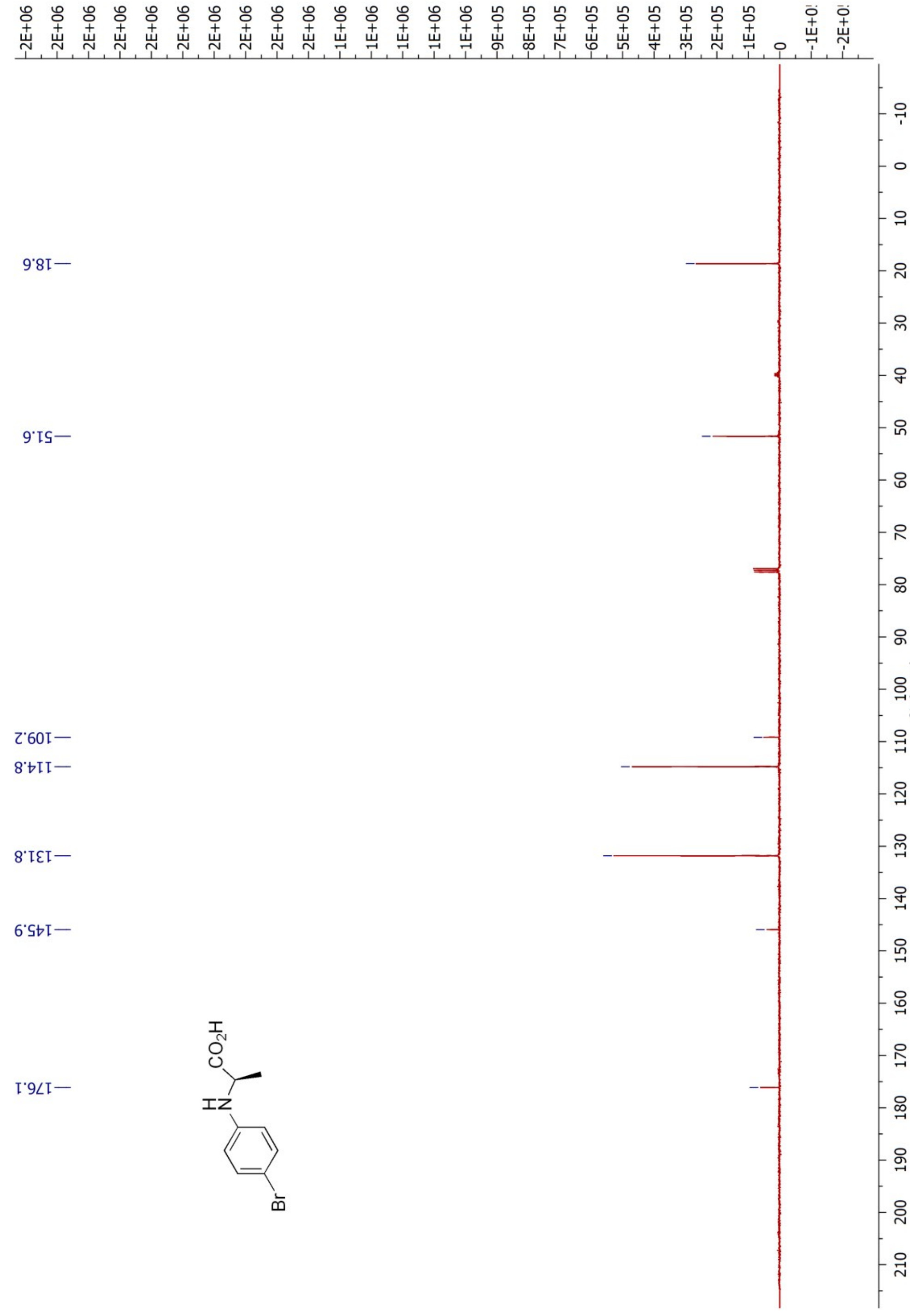

Figure 29S. ^{13}C NMR spectrum of **7e** (100 MHz in $CDCl_3$)

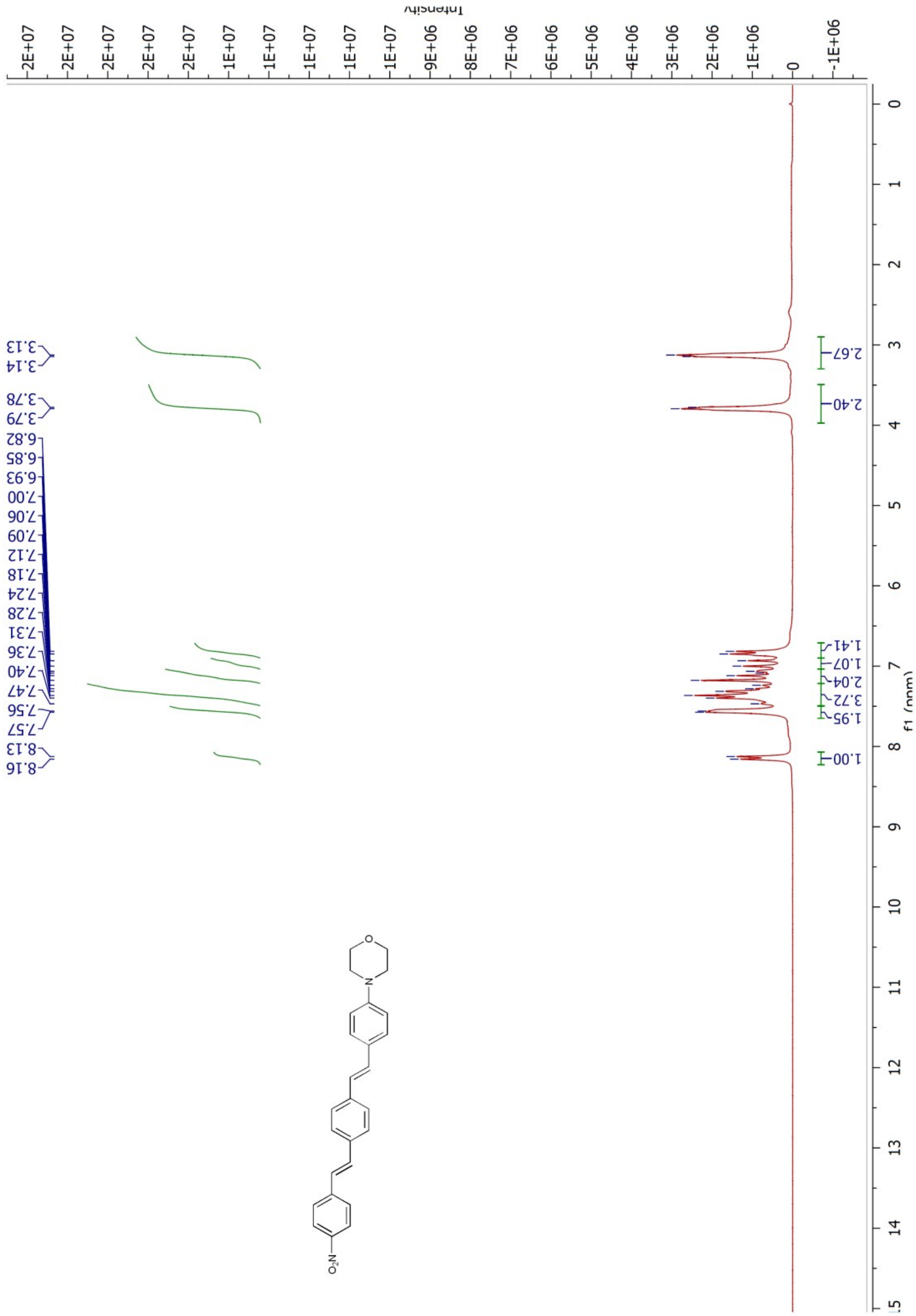

Figure 30S. ^{1}H NMR spectrum of **8a** (250 MHz in $CDCl_3$)

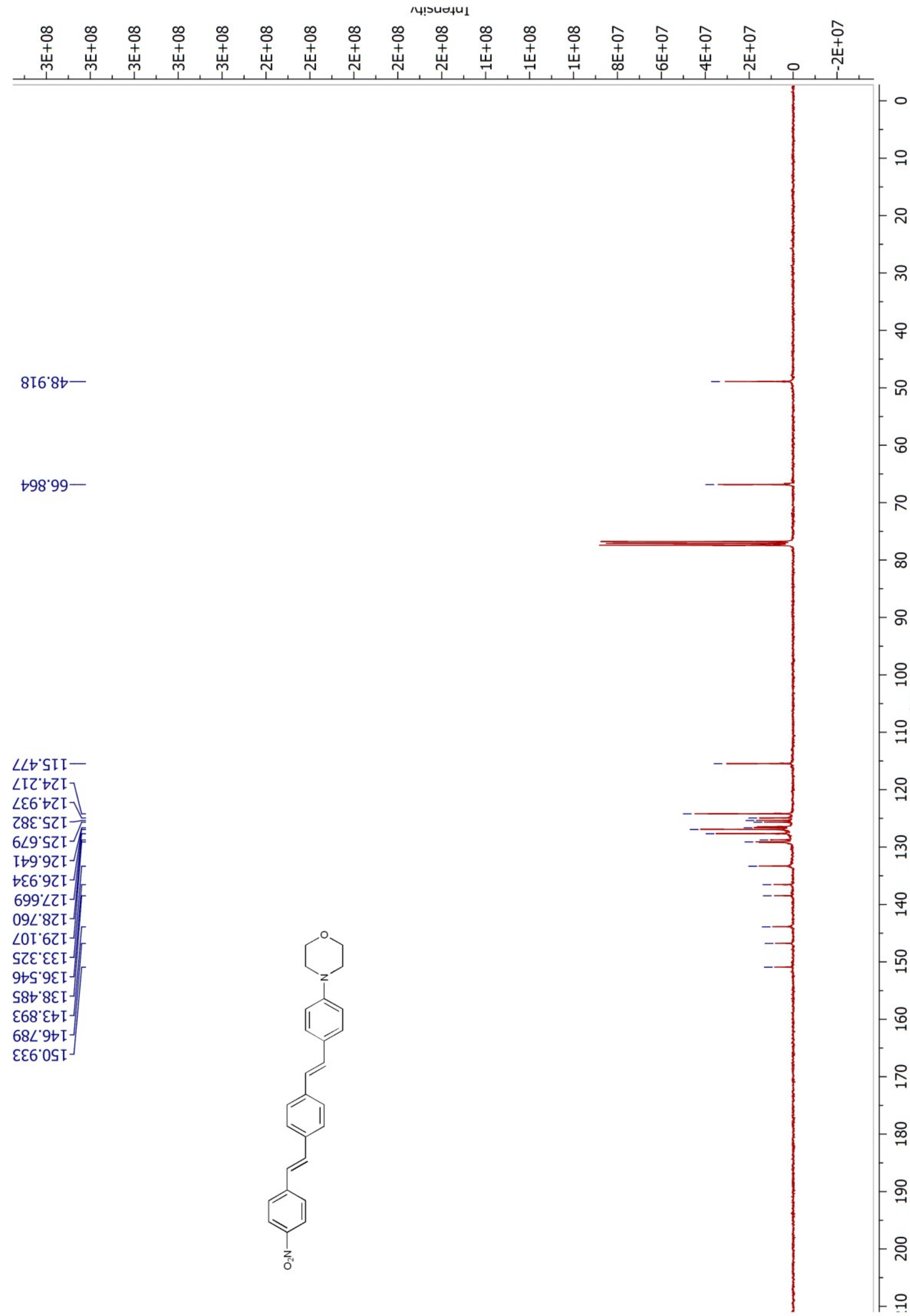

Figure 31S. ^{13}C NMR spectrum of **8a** (62.5 MHz in $CDCl_3$)

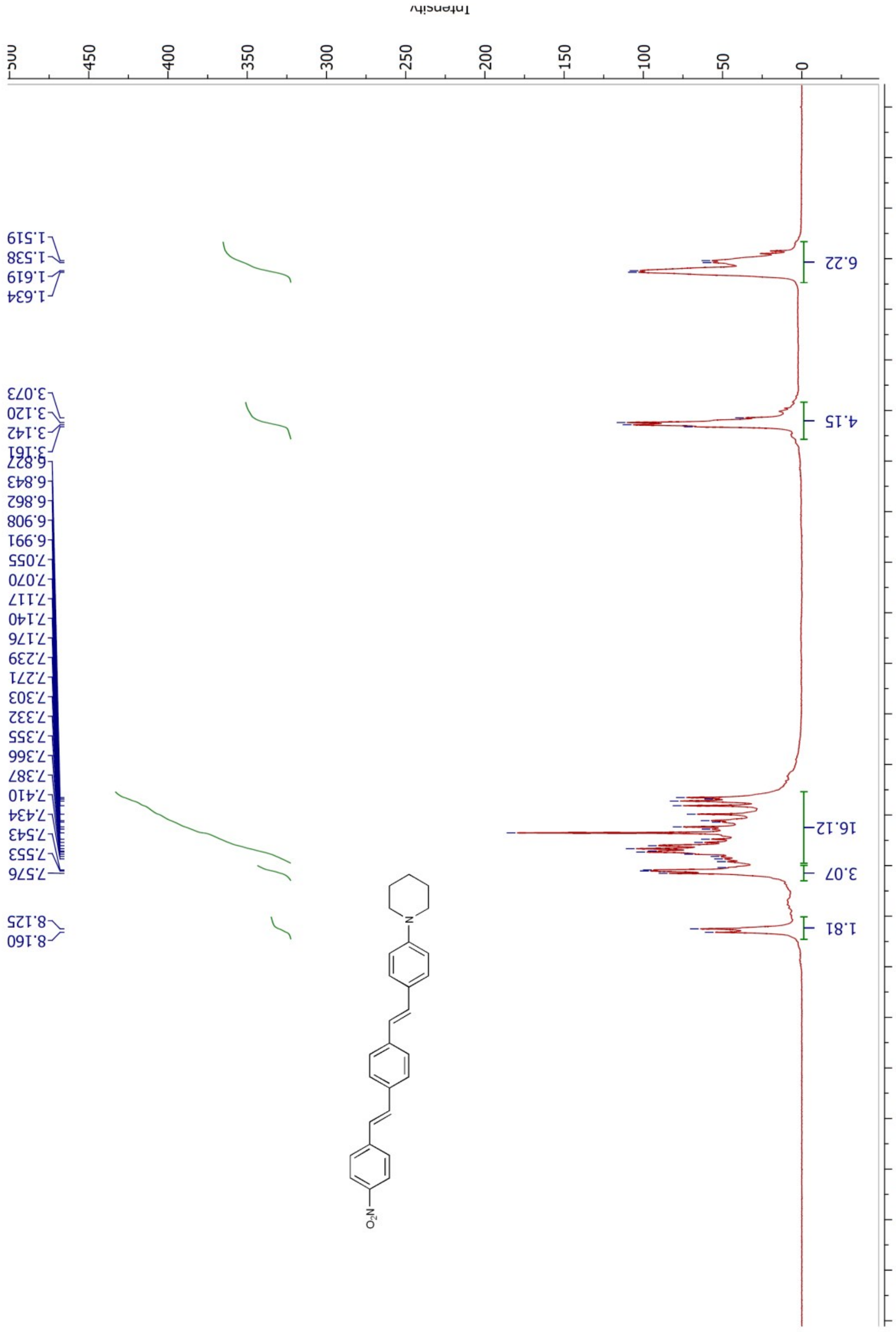

Figure 32S. ^{1}H NMR spectrum of **8b** (250 MHz in $CDCl_3$)

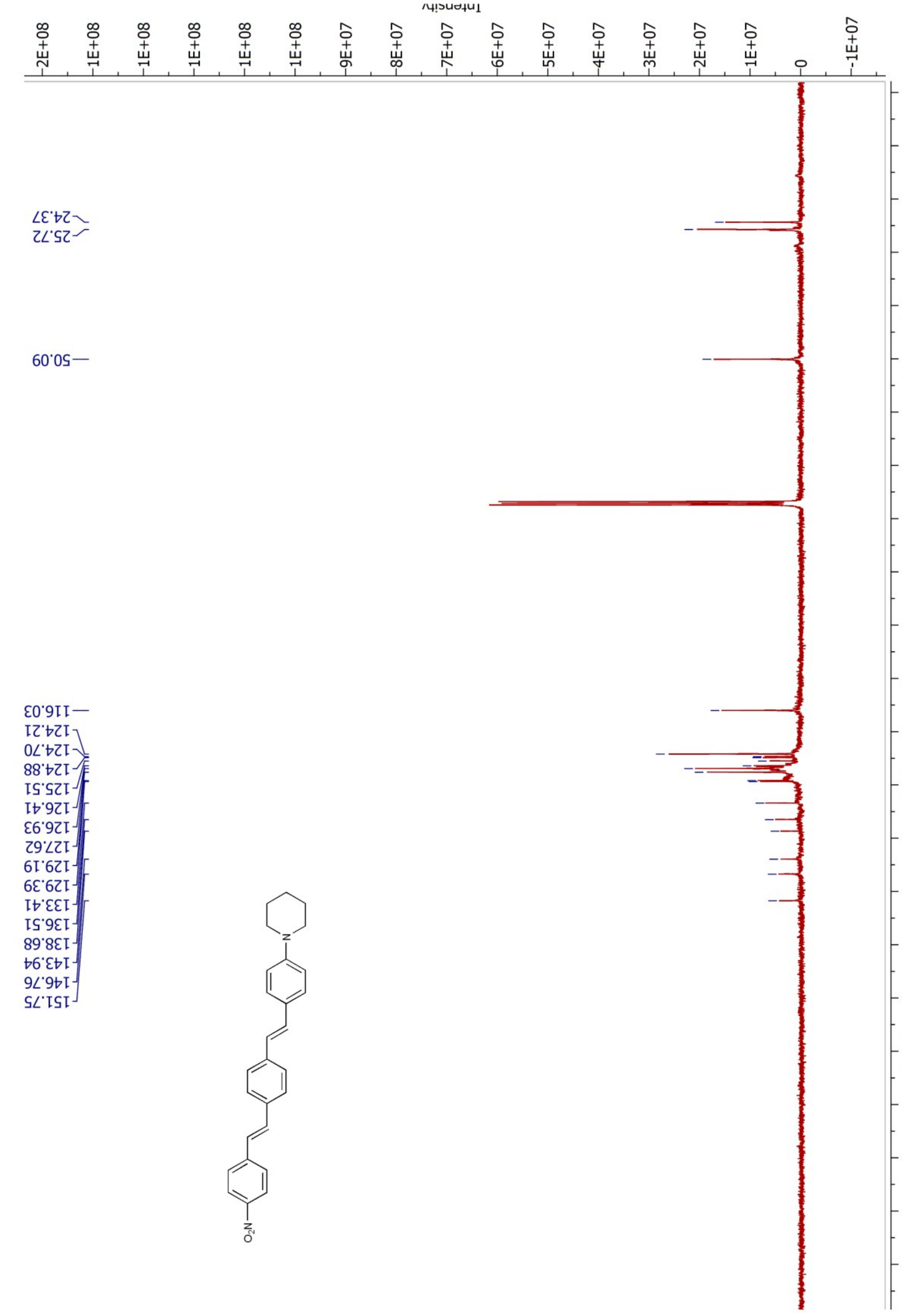

Figure 33S. ^{13}C NMR spectrum of **8b** (62.5 MHz in $CDCl_3$)

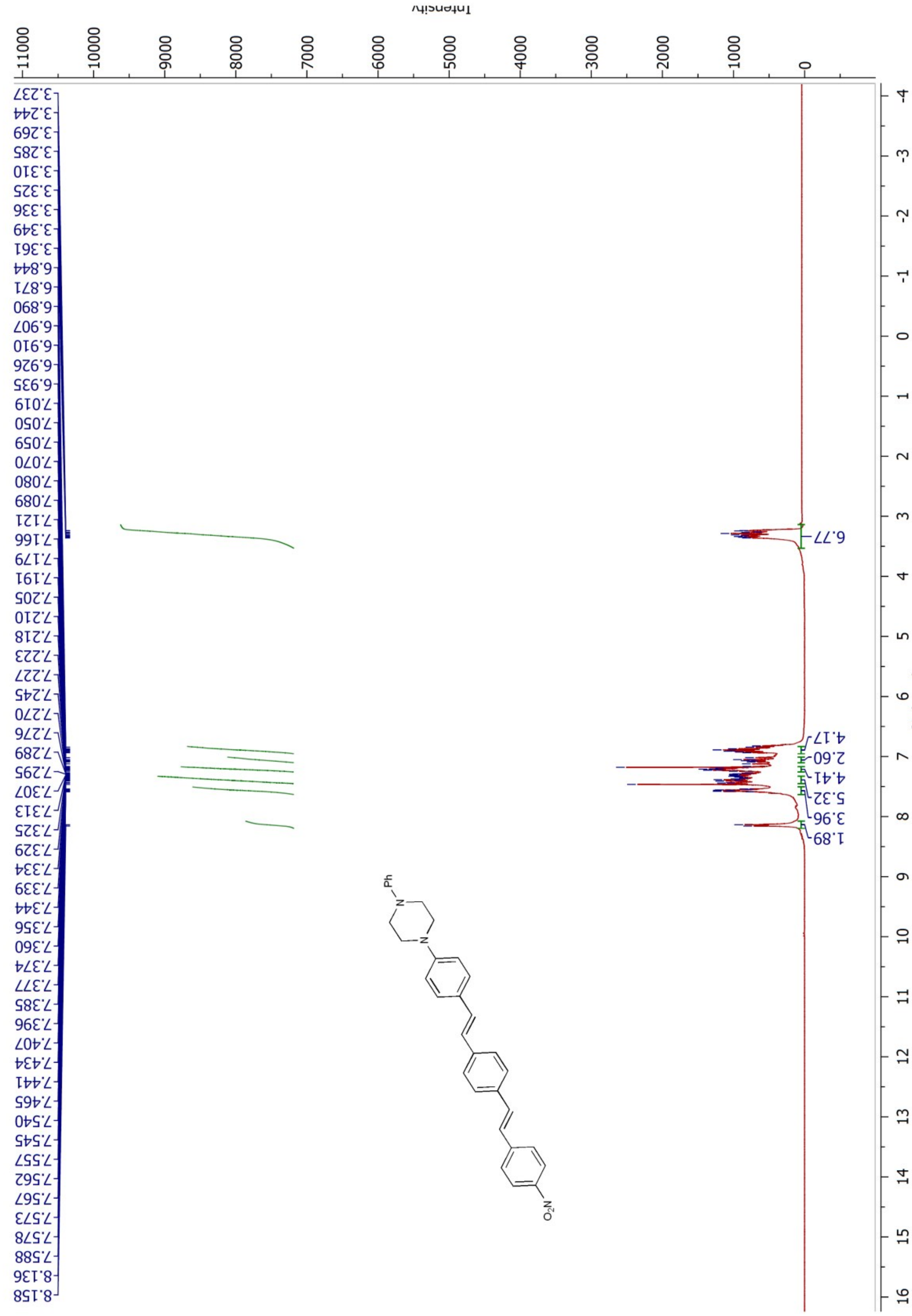

Figure 34S. ^{1}H NMR spectrum of **8c** (250 MHz in $CDCl_3$)

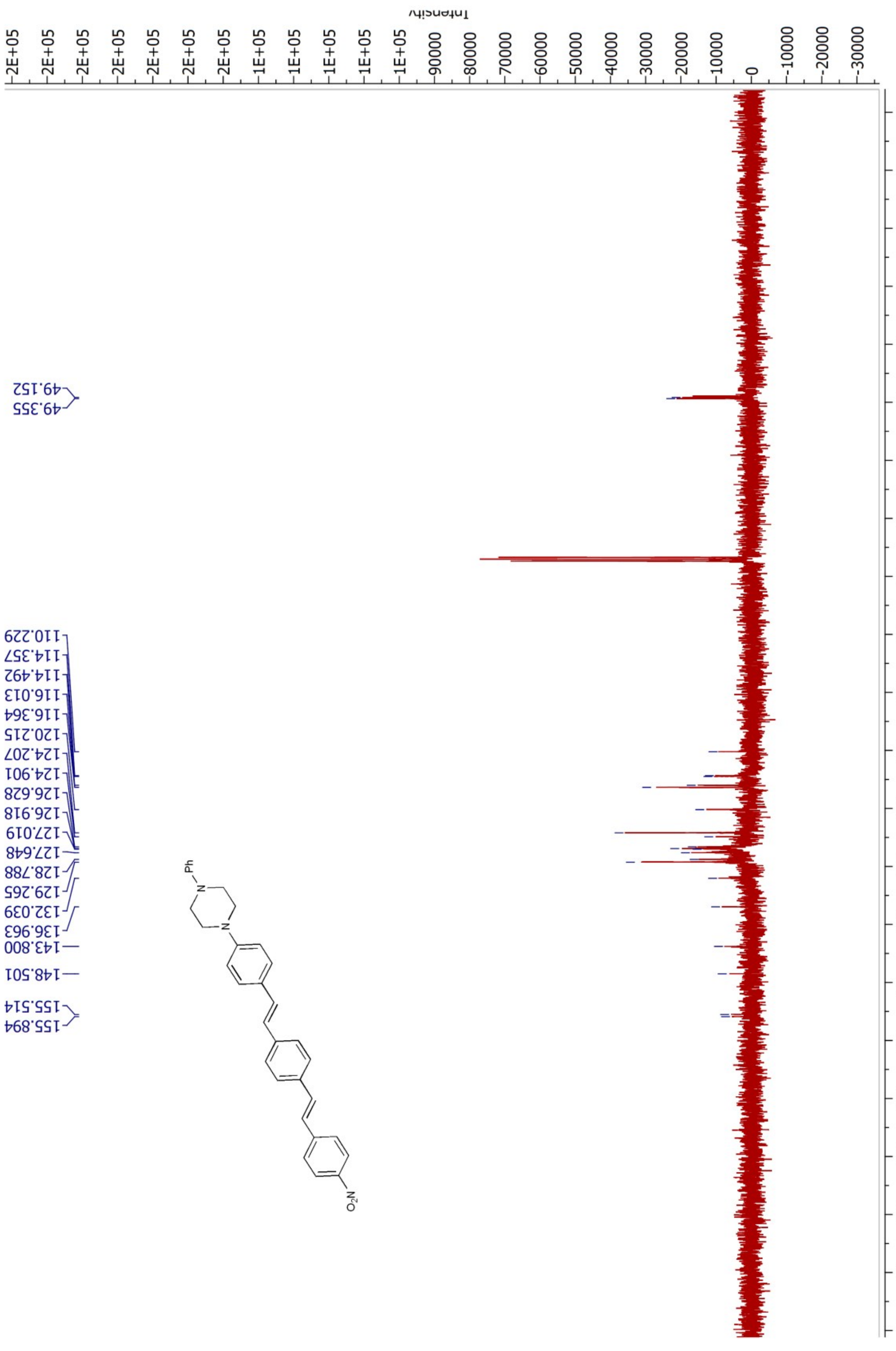

Figure 34S. ^{13}C NMR spectrum of **8c** (100 MHz in $CDCl_3$)

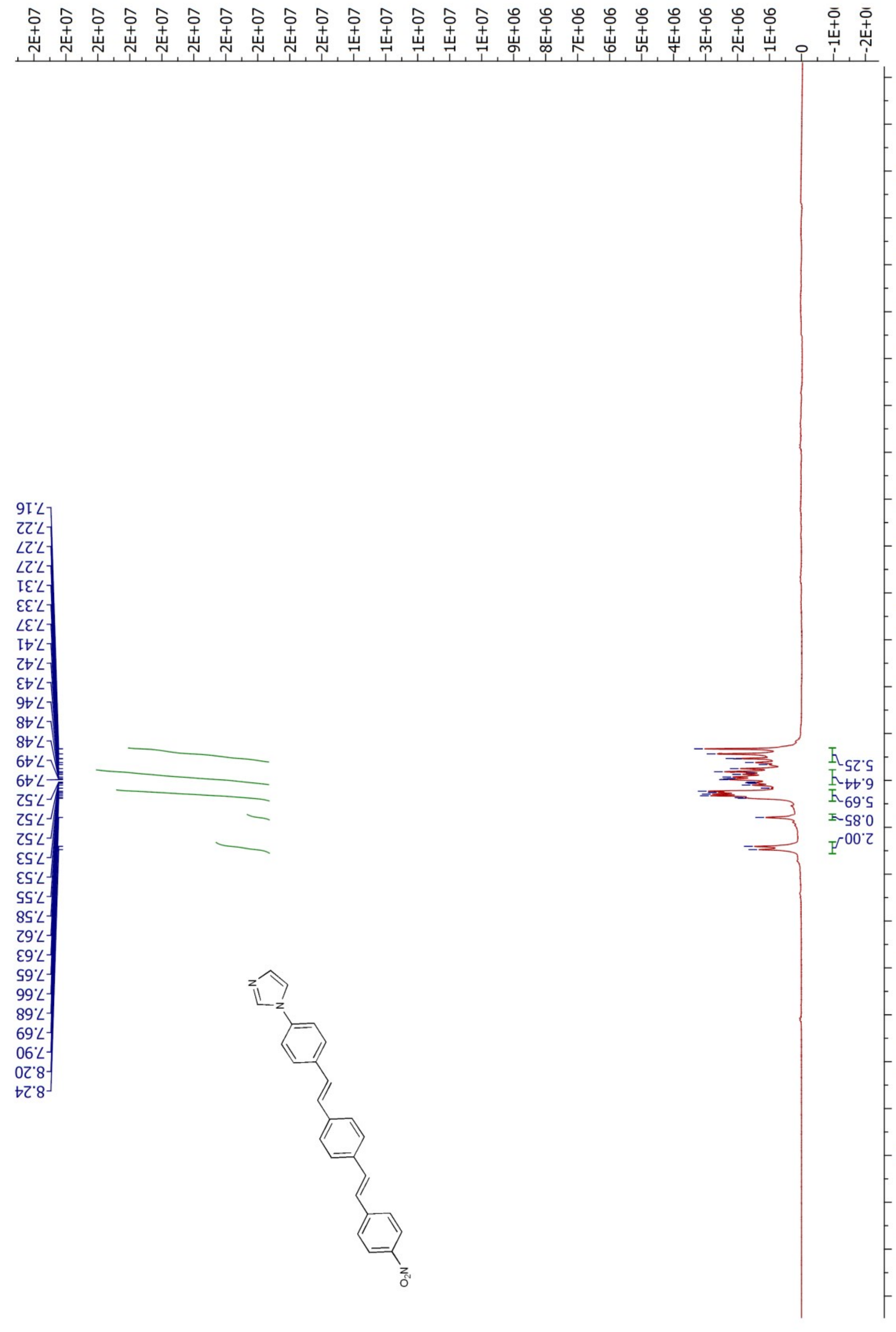

Figure 35S. ^{1}H NMR spectrum of **8d** (250 MHz in $CDCl_3$)

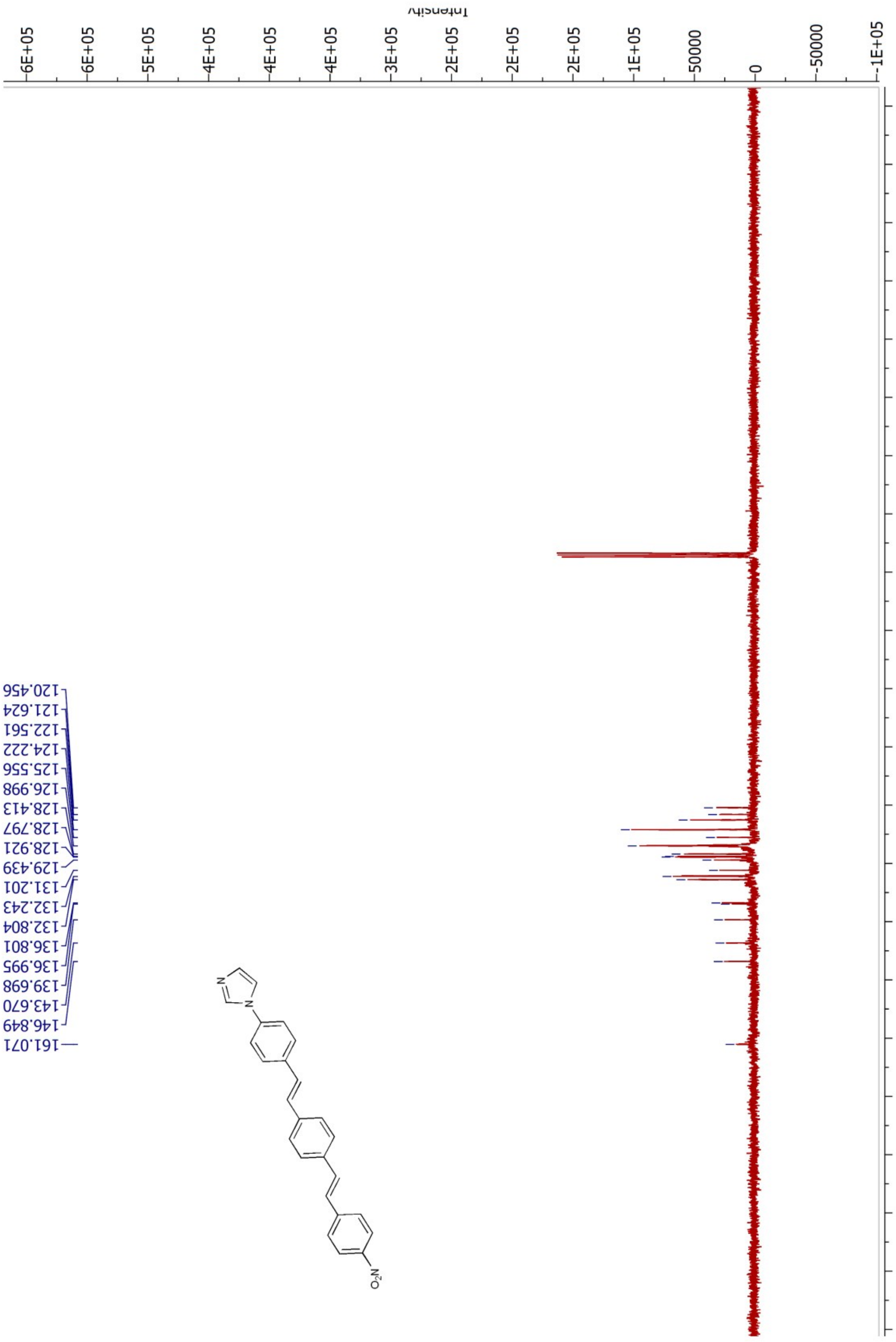

Figure 36S. ^{13}C NMR spectrum of **8d** (100 MHz in $CDCl_3$)

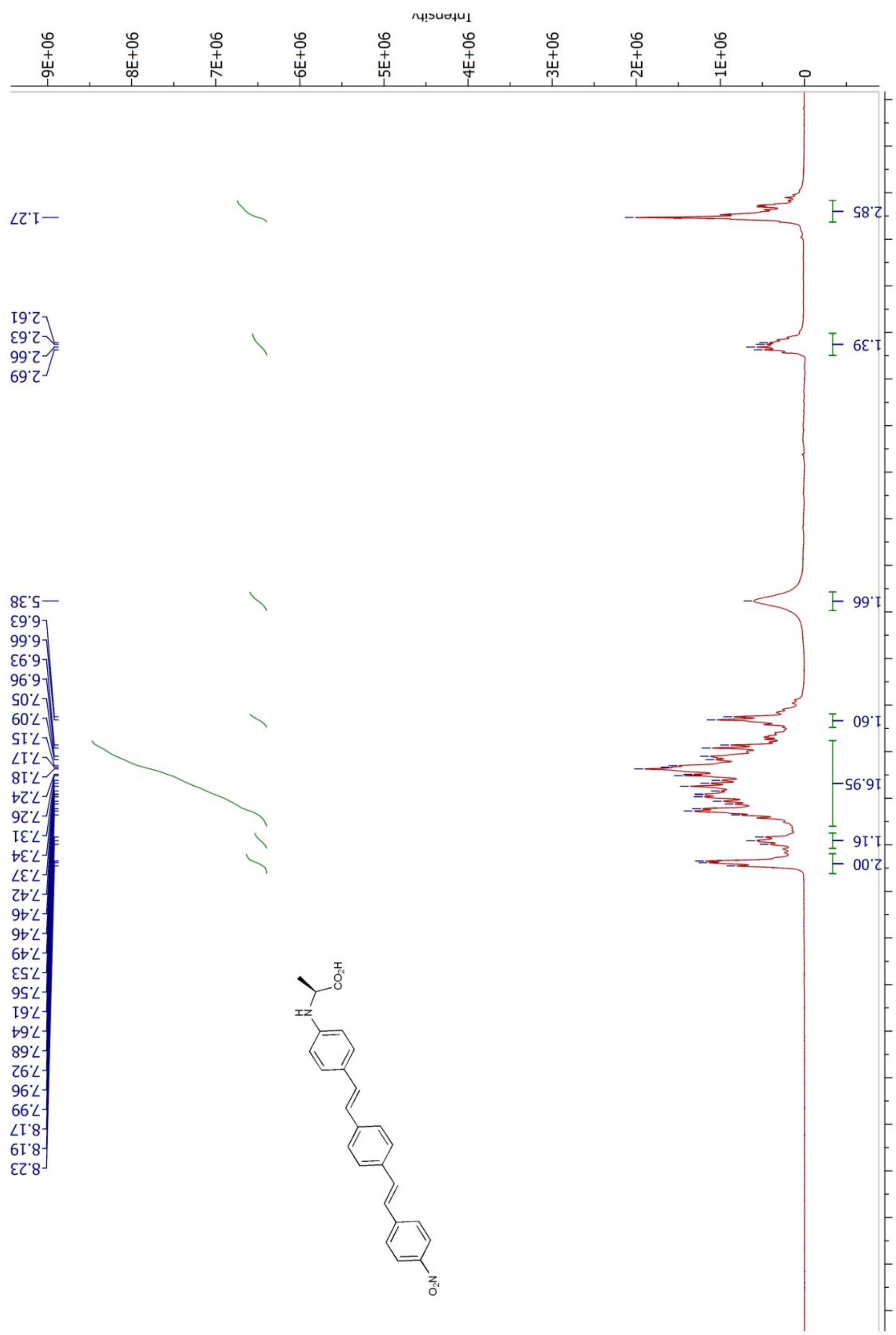

Figure 37S. ^{1}H NMR spectrum of **8e** (250 MHz in $CDCl_3$)

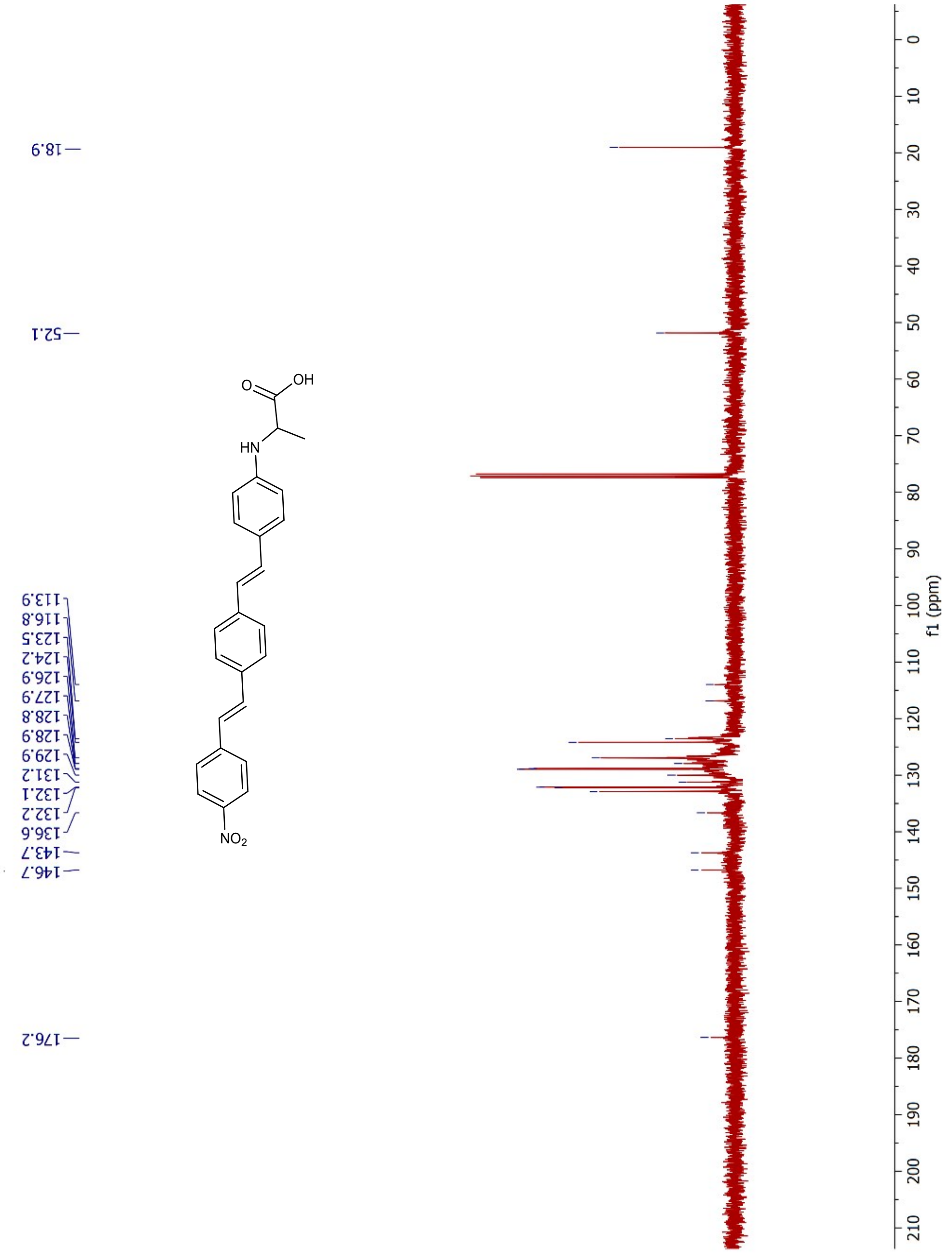

Figure 38S. ^{13}C NMR spectrum of **8e** (100 MHz in $CDCl_3$)
